# Dynamic covalent self-assembly and self-sorting processes in the formation of imine-based macrocycles and macrobicyclic cages†

**DOI:** 10.1039/d3sc01174g

**Published:** 2023-06-02

**Authors:** Zhaozheng Yang, Ferran Esteve, Cyril Antheaume, Jean-Marie Lehn

**Affiliations:** a Lehn Institute of Functional Materials (LIFM), Sun Yat-Sen University 510006 Guangzhou China; b Laboratoire de Chimie Supramoléculaire, Institut de Science et d'Ingénierie Supramoléculaires (ISIS), Université de Strasbourg 8 allée Gaspard Monge 67000 Strasbourg France lehn@unistra.fr

## Abstract

Investigating the self-assembly and self-sorting behaviour of dynamic covalent organic architectures makes possible the parallel generation of multiple discrete products in a single one pot procedure. We here report the self-assembly of covalent organic macrocycles and macrobicyclic cages from dialdehyde and polyamine components *via* multiple [2 + 2] and [3 + 2] polyimine condensations. Furthermore, component self-sorting processes have been monitored within the dynamic covalent libraries formed by these macrocycles and macrobicyclic cages. The progressive assembly of the final structures involves intermediates which undergo component selection and self-correction to generate the final thermodynamic constituents. The homo-self-sorting observed seems to involve entropic factors, as the homoleptic species present a higher symmetry than the competing heteroleptic ones. This study not only emphasizes the importance of an adequate design of the components of complex self-sorting systems, but also verifies the conjecture that systems of higher complexity may generate simpler outputs through the operation of competitive self-sorting.

## Abstract 2

The self-assembly of imine-based dynamic covalent architectures involves component selection and self-correction processes to generate homo-self-sorted outputs, even in dynamic constitutional networks of high complexity.

## Graphical abstract

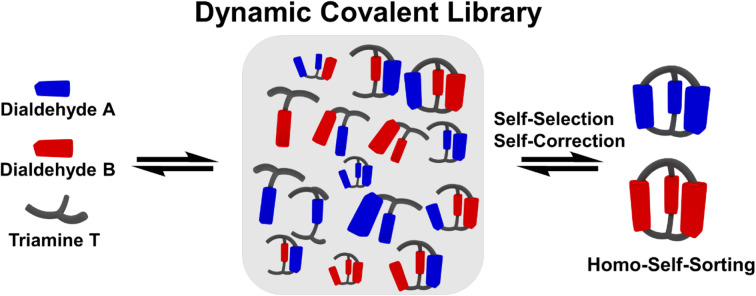

## Introduction

The active development of dynamic covalent chemistry (DCC) offers a powerful tool to chemists for generating well-defined discrete organic architectures from a mixture of components connected by reversible covalent bonds.^1^ In particular, it has provided novel perspectives relating to previous studies of dynamic macrocyclic and macrobicyclic architectures assembled through the formation of multiple reversible imine bonds between amines and carbonyl components, such as tetraimino-macrocycles and the hexaimino macrobicyclic cryptand-type molecular cages.^2^ As is the case for their non-dynamic macrobicyclic analogues, the intramolecular cavity of such well-defined structures may lead to applications such as sensing^3^ and catalysis.^4^ However, from the perspective of their synthetic methodology, the dynamically assembled structures offer new features such as self-sorting and adaptation.^5^ Self-sorting systems based on the dynamic covalent libraries (DC_ov_Ls) generated by DCC operate by incorporation, decorporation and exchange of components following the agonistic and antagonistic relationships between the constituents they build up.^6^

The selective formation of specific architectures along the array of many possible reactions occurring within the DC_ov_L *via* self-sorting of the components is of especially interest. The generation of such well-defined architectures by component self-sorting from multi-component DC_ov_Ls involves competition between the formation of: (i) discrete homoleptic self-sorted constituents (homo-self-sorting);^6^ (ii) mixed component heteroleptic self-sorted constituents (hetero-self-sorting);^7^ (iii) mixed constituents containing different components (scrambling).^8^ The challenge is to control the system in such a way, that the increase in molecular complexity (increase in the number of components and of reacting groups on a single component) will not result in totally random connections from scrambled condensations or polymer formation due to the similarity of bond energies. To meet this challenge, it is crucial to decipher the self-sorting mechanism and to take advantage of it. Thus, tracking the formation of products and understanding the conversion of intermediates are of particular interest and will be helpful for the prediction of self-sorting outcomes.

Recently, we reported the concurrent formation of two representative architectures of different cyclic order, tetraimino macrocycles and hexaimino macrobicyclic cages, from DC_ov_Ls of a diamine, a triamine and dialdehydes.^6*e*^ These studies revealed the occurrence of kinetic switching *via* component exchange in the course of the formation of macrocycles and macrobicyclic cages by self-sorting. The present work focuses on the multiple imine condensation processes and multi-component covalent self-sorting mechanisms taking place in such macrocycles and macrobicyclic cages. It examines correlations between the selection of specific components/building blocks of different shape and size and the structural features of the resulting species. A significant feature of these self-sorting processes resides in the self-correction behaviour that occurs based on reversible imine condensations. Finally, a [4 × 2] six-component DC_ov_L of higher order complexity was set up by an appropriate choice of DC_ov_L precursors, four different dialdehydes and two amines (a triamine and a diamine). In this case, only specific imine architectures, one macrobicyclic cage and three agonistic macrocycles, were produced *via* the self-sorting processes.

## Results and discussion

**Scheme 1:**
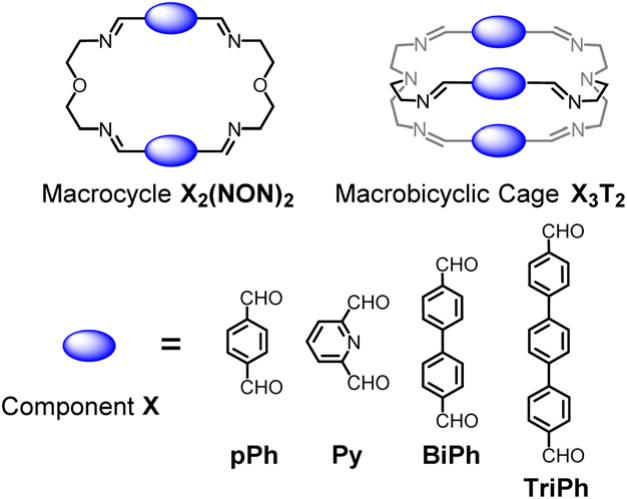
Molecular structures of the dialdehyde components (bottom) and of the homoleptic imine-based constituents: the [2 + 2] macrocycles and the [3 + 2] macrobicyclic cages (top) studied in the present work. The macrocycles TriPh_2_(NON)_2_ and the macrobicycle TriPh_3_T_2_ are new compounds. The macrocycles *p*Ph_2_(NON)_2_,^10^Py_2_(NON)_2_,^11^BiPh_2_(NON)_2_,^6*e*^ and the macrobicycles *p*Ph_3_T_2_,^12^Py_3_T_2_,^2*a*^BiPh_3_T_2_,^9*a*^ have been previously reported in the literature as indicated.

Previous work has described the synthesis and characterization of macrocyclic and macrobicyclic cryptand-type structures, as well as their self-sorting behaviour.^6*e*,9^ These studies have now been extended to the investigation of the formation mechanisms and component selection features of homoleptic macrocycles and macrobicyclic cages in imine-based dynamic covalent systems. To this end, the formation of the *D*_2h_-symmetric tetraimino macrocycles and *D*_3h_-symmetric hexaimino macrobicycles shown in Scheme 1 from their dialdehyde and diamine or triamine components were monitored by ^1^H NMR (in CDCl_3_ at 23–25 °C) and by HRMS (in 50/50 (v/v) CHCl_3_/MeOH at 25 °C).

**Fig. 1:**
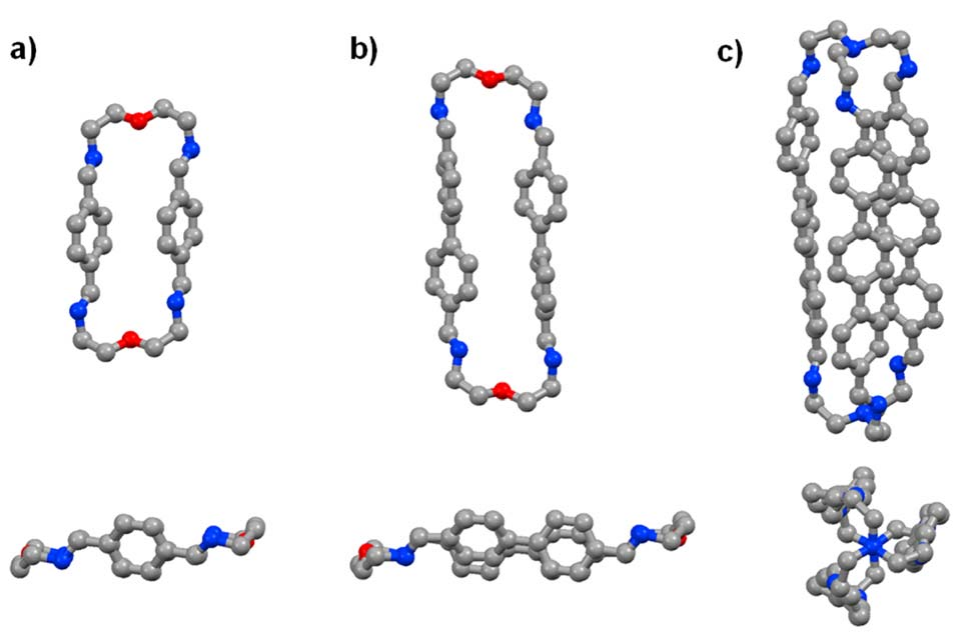
Single-crystal X-ray structures of the macrocycles *p*Ph_2_(NON)_2_ and BiPh_2_(NON)_2_ as well as of the macrobicyclic cage TriPh_3_T_2_ (side and front/axial views). Colour scheme: carbon (grey), nitrogen (blue), oxygen (red). Hydrogen, H_2_O and CHCl_3_ molecules are omitted for clarity, see details in ESI, Fig. S63–S65.†

The time evolution curves shown in the figures below depend on the experimental conditions (see ESI Page S5†). As also observed previously, due to the formation of a variety of intermediates, the consumption of aldehyde components is much faster than the appearance of the final products. Both the ^1^H NMR spectra and the HRMS spectra were recorded immediately after addition of 2 equiv. 2,2′-oxybis(ethylamine) (NON) into 2 equiv. dialdehyde solution or 2 equiv. tris(2-aminoethyl)amine (T) into 3 equiv. dialdehyde solution. The reaction rates can be estimated by the time of 50% dialdehyde consumption (*t*^C^_1/2_) and the time of 50% product formation (*t*^F^_1/2_). The results obtained are given in the ESI (see the NMR monitoring sections in ESI, Pages S7–S25†). The molecular structures of *p*Ph_2_(NON)_2_, BiPh_2_(NON)_2_ and TriPh_3_T_2_ were confirmed by single-crystal X-ray crystallography (Fig. 1; see details in ESI, Fig. S63–S65 and Table S9†).‡‡Crystals suitable for X-ray analyses were obtained for macrocycles *p*Ph_2_(NON)_2_ and BiPh_2_(NON)_2_ and for the cage TriPh_3_T_2_. Both macrocyclic structures revealed a *C*_2_-symmetry enforced by intramolecular π⋯π interactions, with the shortest C_ar_⋯C_ar_ distance of 3.840 Å and 3.585 Å, respectively. Despite the long distances between the oxygen atoms −*d*_O⋯O_ = 10.584 and 15.116 Å for *p*Ph_2_(NON)_2_ and BiPh_2_(NON)_2_ – no solvent molecules were observed within the cavity. A water molecule was found to be interacting with the lone pair of the N atoms (imino groups) in *p*Ph_2_(NON)_2_, with a N⋯O_water_ distance of 2.896 Å. The TriPh_3_T_2_·molecular structure showed a distorted *D*_3_ symmetry and the aromatic bridging units were also interacting through π⋯π forces (shortest C⋯C distance = 3.425 Å). These intramolecular interactions were likely compressing the 3D-conformation of the cyclic species and thus reducing the cavity void, hampering the encapsulation of guest molecules.

### Formation and characterization of the homoleptic macrocycles *p*Ph_2_(NON)_2_ and BiPh_2_(NON)_2_ from their components as a function of time in imine-based dynamic covalent systems

**Fig. 2:**
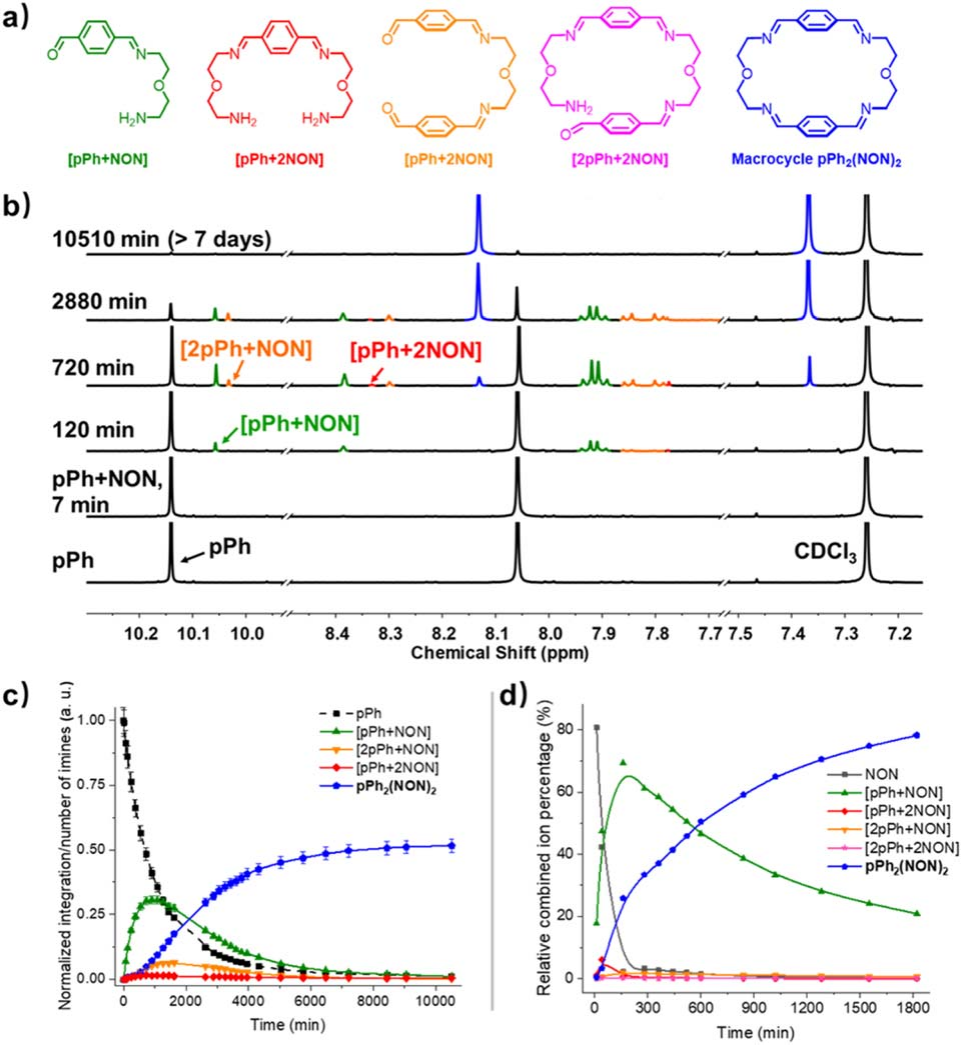
(a) Chemical structures of the different intermediates and of *p*Ph_2_(NON)_2_, (b) evolution of the ^1^H NMR spectra (500 MHz, CDCl_3_, 23 °C) of a 1 : 1 mixture of *p*Ph and NON ([*p*Ph]_0_ = [NON]_0_ = 3.6 mM) showing the formation of three intermediates and of the final macrocycle *p*Ph_2_(NON)_2_ after 7 min, 120 min, 720 min, 2880 min and 10 510 min. (c) ^1^H NMR monitoring of the evolution of the species generated during *p*Ph_2_(NON)_2_ formation from a 1 : 1 mixture of *p*Ph and NON as a function of time over 10 510 min. Error in ^1^H-NMR signal integration: ±5%. (d) HRMS-ESI monitoring of the species generated during the formation of the macrocycle *p*Ph_2_(NON)_2_ from a 1 : 1 mixture of *p*Ph and NON ([*p*Ph]_0_ = [NON]_0_ = 2 mM, 50/50 (v/v) CHCl_3_/MeOH, 25 °C) over 1820 min. The spectra and kinetic curves are coloured in accordance with the molecular structures. NB: the relative combined ion percentage is obtained by the ratio of combined ion count of each species to the sum of combined ion counts for all species at each time point. These data do not provide quantitative information about the relative amounts of each species identified by its mass, but, taken separately, they display the evolution of a given identified species during the course of the reaction. The curves are added to guide the eye.

The multiple condensation reactions involved in the formation of compounds containing several imine groups from their carbonyl and amine components lead to the generation of numerous transient intermediates which greatly complicates the ^1^H NMR investigation of the reaction course. In order to minimize the analysis difficulties, we first studied the macrocycle *p*Ph_2_(NON)_2_ formation from terephthalaldehyde (*p*Ph) and 2,2′-oxybis(ethylamine) (NON), as there is only a singlet ^1^H NMR signal in the aromatic area of the dialdehyde *p*Ph. Throughout the measurement, signals due to several metastable intermediates were observed. Four possible acyclic intermediates may be considered, with different dialdehyde : diamine compositions: [*p*Ph + NON], [*p*Ph + 2NON], [2*p*Ph + NON], [2*p*Ph + 2NON] (Fig. 2a), and could be identified from their ^1^H NMR signals according to their different structural features. For example, the [1 + 1] unsymmetrical acyclic [*p*Ph + NON] intermediate should form first and show one aldehyde signal, one imine signal, two peaks in the aromatic area and four peaks in aliphatic area. These spectroscopic characteristics coincide with the green peaks in Fig. 2b. The orange peaks are those of symmetrical [2*p*Ph + NON]. For the remaining red signals in Fig. 2b, two singlets at 8.33 ppm and 7.78 ppm can then be assigned to the symmetrical [*p*Ph + 2NON]. Ultimately, all these intermediates are converted into the macrocycle *p*Ph_2_(NON)_2_ (Fig. 2b, blue). The evolution of the reaction mixture is plotted in Fig. 2c. It can be seen that the intermediate [*p*Ph + NON] markedly increased with time from 7 to 900 min to reach 30% of the maximum possible amount and then smoothly decreased, while smaller quantities of [2*p*Ph + NON] and [*p*Ph + 2NON] showed a similar but somewhat slower time dependence.

The evolution of the reaction mixture was also monitored by HRMS, providing additional information on the identification of intermediates. All the possible intermediates were identified, including [2*p*Ph + 2NON], indiscernible in the ^1^H NMR spectra. The evolution of their *m*/*z* peak intensities over time is plotted in Fig. 2d. Both the ^1^H NMR and HRMS curves for the macrocycle product show an induction period, consistent with its formation involving passage through at least one intermediate.^6*e*^ A similar behaviour was found for the formation of BiPh_2_(NON)_2_ as indicated by the corresponding ^1^H NMR and HRMS data. [BiPh + NON] and [2BiPh + NON] compositions were detected and identified by ^1^H NMR spectroscopy (Fig. S9–S12†) and HMRS (Fig. S16 and S17†).

**Scheme 2:**
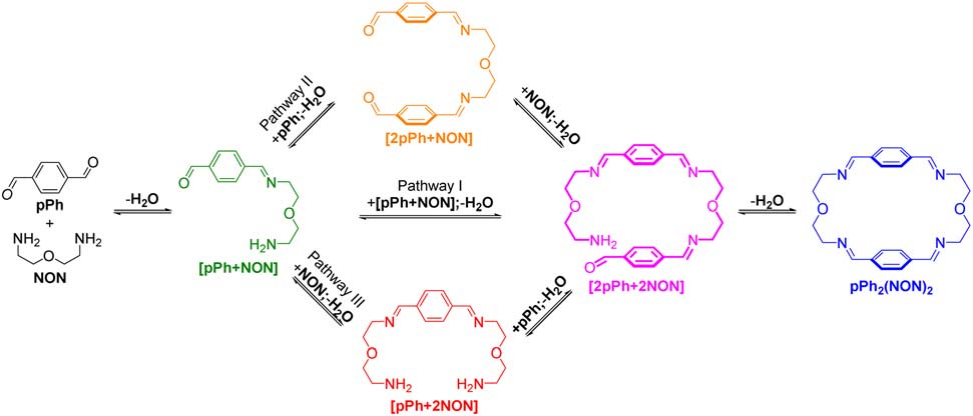
Stepwise [2 + 2] imine condensation processes showing the intermediates on the way to the formation of the imine-based dynamic covalent macrocycle *p*Ph_2_(NON)_2_.

A possible self-assembly mechanism of such [2 + 2] covalent organic macrocycles can be proposed as shown in Scheme 2. Thus, in the case of *p*Ph_2_(NON)_2_ for example, initial formation of the [1 + 1] intermediate [*p*Ph + NON] is followed by generation of [2*p*Ph + 2NON] along three pathways, (i) (main process): one-step dimerization of two [*p*Ph + NON] intermediates; (ii) and (iii): condensation with an additional component (*p*Ph or NON) to form intermediates [1 + 2] and [2 + 1]; finally, intramolecular macrocyclization yielding macrocycle *p*Ph_2_(NON)_2_. At the beginning, with only *p*Ph and NON present, only [*p*Ph + NON] can form. If then it reacts further with itself or with *p*Ph or NON separately at a rate similar to that of its formation, then it should start to generate [2*p*Ph + 2NON] (which undergoes a rapid intramolecular cyclization) along with [2*p*Ph + NON] and [*p*Ph + 2NON]. The latter two then have to react with free NON and *p*Ph respectively to give [2*p*Ph + 2NON] and thus the macrocycle by cyclization but at relatively slow rates because the concentrations of free *p*Ph and NON are now quite low compared to the initial values controlling the formation of [*p*Ph + NON].

### Formation and characterization of the homoleptic macrobicyclic cages *p*Ph_3_T_2_ and BiPh_3_T_2_ from their components as a function of time in imine-based dynamic covalent systems

The assembly of cage *p*Ph_3_T_2_ was monitored by ^1^H NMR spectroscopy and HRMS. In the ^1^H NMR spectra, signals from several intermediates were observed. As there may be nine possible intermediates for the generation of a cage, it is not surprising that the spectra are more complicated than in the case of the macrocycles.

**Fig. 3:**
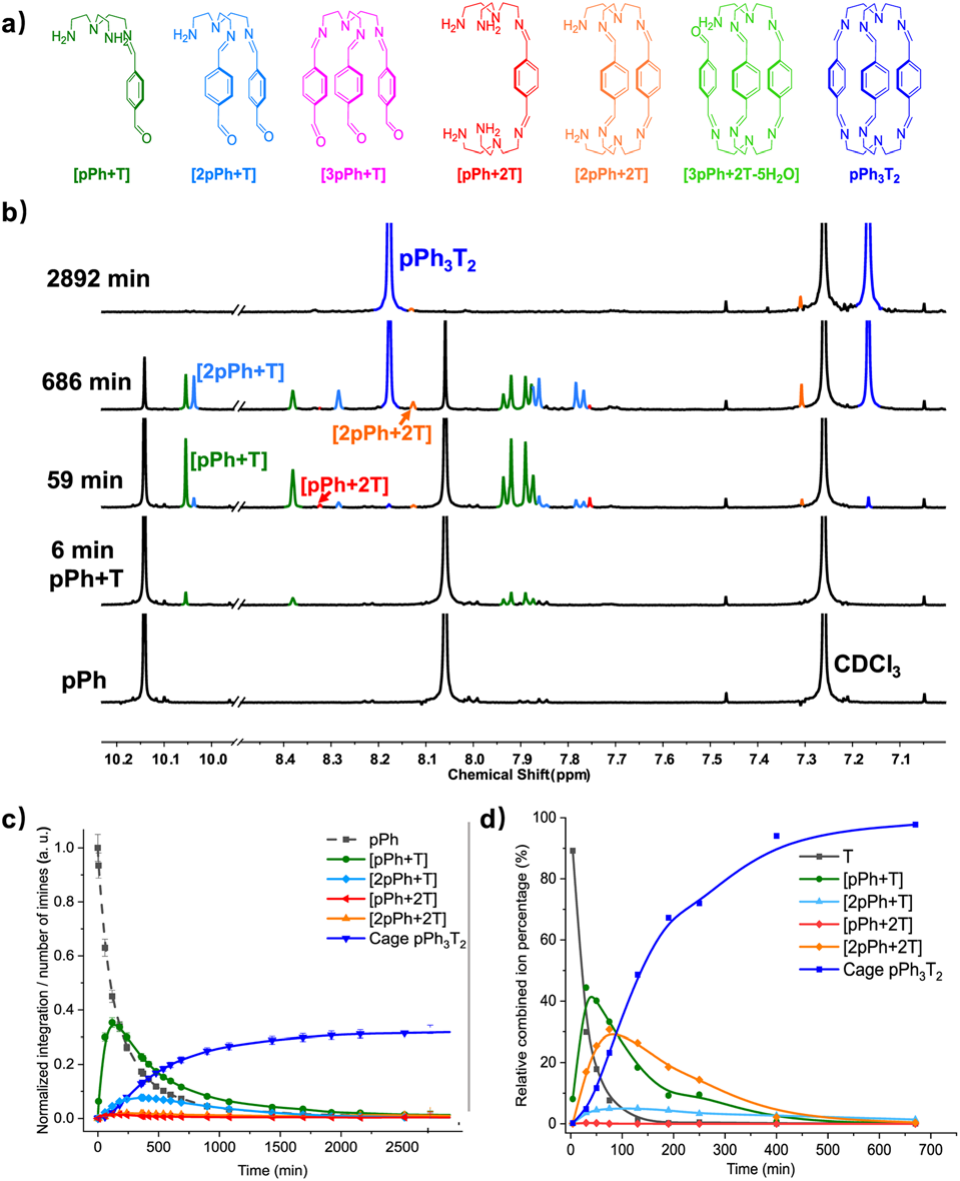
(a) Chemical structures of the different intermediates and of *p*Ph_3_T_2_. (b) Evolution of the ^1^H NMR spectra (500 MHz, CDCl_3_, 23 °C) of a 3 : 2 mixture of *p*Ph (3.6 mM) and T showing the formation of intermediates [*p*Ph + T], [2*p*Ph + T], [*p*Ph + 2T], and [2*p*Ph + 2T] together with that of the final macrobicyclic cage *p*Ph_3_T_2_. (c) ^1^H NMR monitoring of the evolution of the species generated during cage *p*Ph_3_T_2_ formation as a function of time over 2892 min. Error in ^1^H-NMR signal integration: ±5%. (d) HRMS-ESI kinetic evolution of the species generated during the formation of the macrocycle *p*Ph_3_T_2_ from *p*Ph (2 mM) and T (50/50 CHCl_3_/MeOH, r. t). The spectra and kinetic curves are coloured in accordance with the molecular structures. NB: the relative combined ion percentage is obtained by the ratio of combined ion count of each species to the sum of combined ion counts for all species at each time point. These data do not provide quantitative information about the relative amounts of each species identified by its mass, but, taken separately, they display the evolution of a given identified species during the course of the reaction. The curves are added to guide the eye.

**Scheme 3:**
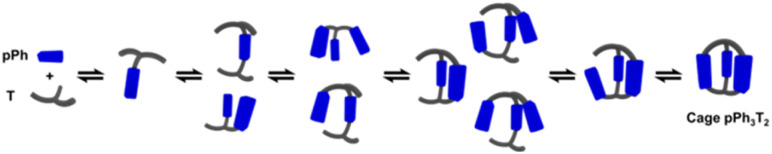
Stepwise [3 + 2] imine condensation processes showing the intermediates corresponding to an increase in number of imine connections on the way to the formation of a dynamic covalent macrocyclic cage.

Throughout the ^1^H NMR, several metastable intermediates, for instance [*p*Ph + T], [2*p*Ph + T], [*p*Ph + 2T], and [2*p*Ph + 2T] could be identified (Fig. 3a, for assignment of ^1^H NMR see ESI Fig. S20, S23 and S24†). The ^1^H NMR and HRMS kinetic monitoring (Fig. 3b and c) showed that all the intermediates were first found to increase and then decrease together with the formation of the final macrobicyclic product. The intermediates [2*p*Ph + T] and [2*p*Ph + 2T] reached their maximum amount later than [*p*Ph + T], indicating a sequential formation of intermediates with increased stoichiometry along the reaction toward the final macrobicyclic cage *p*Ph_3_T_2_. Scheme 3 illustrates all the imine species possibly involved in the dynamic self-assembling process (nine intermediates as well as two reagents and a cage product), although some of them were not detected by HRMS-ESI.

A similar behaviour was found for the formation of cage BiPh_3_T_2_ (at a slower rate) involving also a range of intermediates of different stoichiometries (see ESI Fig. S25–S31, S38 and S39†).

Among all these products, the macrobicyclic cages form faster than their corresponding macrocycles with diamine NON despite the fact that the macrobicycles require six imine condensations and macrocycles only four, a feature possibly related to the higher probability of intramolecular reaction in the first case.^6*e*^

### Self-correction in the self-sorting processes leading to the formation of homoleptic macrocycles

**Fig. 4:**
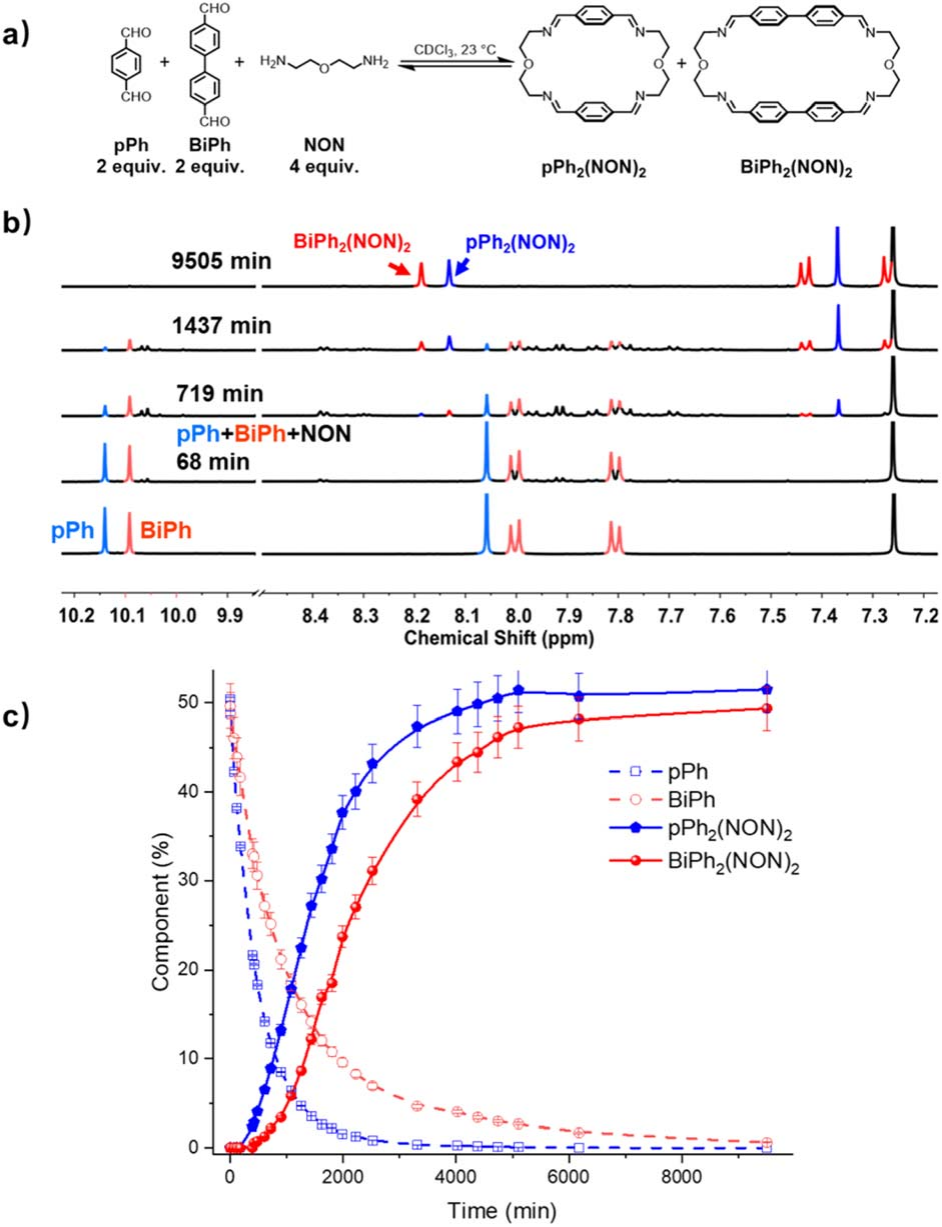
(a) Component self-sorting in the competitive formation of two macrocycles *p*Ph_2_(NON)_2_ and BiPh_2_(NON)_2_. (b) Evolution of the partial ^1^H NMR spectra (500 MHz, CDCl_3_, 23 °C) of the reaction 2*p*Ph + 2BiPh + 4NON ([*p*Ph]_0_ = [BiPh]_0_ = 3.6 mM; [NON]_0_ = 7.2 mM) over 9505 min. The bottom trace corresponds to the dialdehyde components, *p*Ph and BiPh. (c) ^1^H NMR monitoring of dialdehydes *p*Ph, BiPh and macrocycles *p*Ph_2_(NON)_2_ and BiPh_2_(NON)_2_ over 9505 min. The distribution (%) have been obtained by the integration of the of the aromatic regions and aldehyde CHO proton signals in the 500 MHz ^1^H NMR spectra. Error in ^1^H-NMR signal integration: ±5%. The corresponding *t*_1/2_ values are given in the ESI.†

The homo-self-sorting behaviour was investigated for a three-component mixture of two dialdehydes *p*Ph, BiPh of different lengths and NON in a 2 : 2 : 4 ratio. Fig. 4a represents the corresponding ^1^H NMR spectra showing a progressive formation of new molecules which were assigned to different intermediates depending on the formation sequence. The consumption of *p*Ph and BiPh as well as the formation of the two macrocycles, are plotted *versus* reaction time in Fig. 4b. The formation of macrocycle *p*Ph_2_(NON)_2_ was faster than that of BiPh_2_(NON)_2_, which was also related to the faster consumption of *p*Ph than of BiPh. The formation of *p*Ph_2_(NON)_2_, BiPh_2_(NON)_2_ in the self-sorting *p*Ph : BiPh : NON system is faster than that observed for their separate formations (see ESI, Fig. S40†). This may be due to the doubled initial concentration of NON in the self-sorting system. The composition of the system was 51% of *p*Ph_2_(NON)_2_, 49% of BiPh_2_(NON)_2_ and less than 1% of unreacted BiPh at equilibrium (after 160 h; see Fig. 4).§§The composition (component %) of constituents in the self-sorting experiments is presented on the basis of the percentages of the dialdehyde components. The final spectrum indicated the high-fidelity homo-self-sorting and confirmed the absence of heteroleptic macrocycles. During the dynamic self-assembly, one observes several signals belonging to neither the identified intermediates of *p*Ph/NON reaction nor those of BiPh/NON reaction. They may be due to several heteroleptic intermediates (for instance, [*p*Ph + BiPh + NON], [*p*Ph + BiPh + 2NON]) which then disappeared and no heteroleptic macrocycles could be detected, indicating correction and dissociation processes were taking place (see Fig. S41†).

**Scheme 4:**
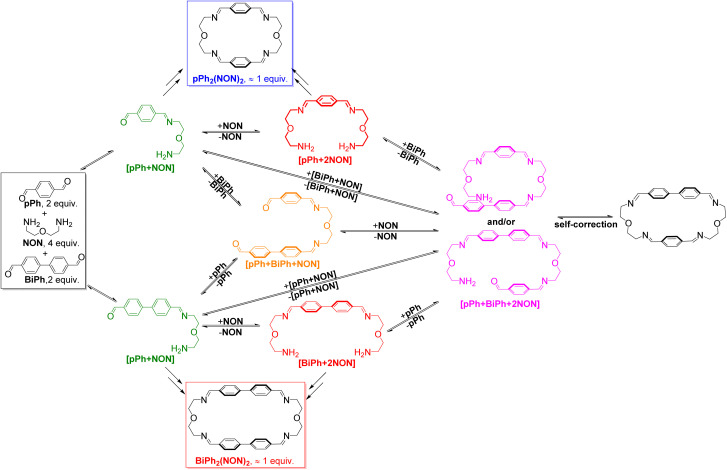
Self-correction and dissociation processes showing the conversion of heteroleptic intermediates on the way to self-sorting of the imine-based dynamic covalent macrocycles *p*Ph_2_(NON)_2_ and BiPh_2_(NON)_2_.

HRMS was also used to identify some intermediates and to follow their evolution as a function of time (see in ESI, Fig. S44 and S45†). The intermediates [*p*Ph + NON], [BiPh + NON] and [*p*Ph + 2NON] were very rapidly formed after mixing the solutions of the three components. They reached their highest abundance after 180 min, and then were smoothly transformed into the desired homoleptic macrocycles. The intermediates [*p*Ph + BiPh + NON] and [*p*Ph + BiPh + 2NON] as well as macrocycle (*p*Ph)(BiPh)(NON)_2_ were detected in the course of the reaction and disappeared latter on. No signals corresponding to a heteroleptic macrocycle could be observed at the end of the reaction. A plausible self-sorting process may be suggested: in an initial step, the diamine NON reacted reacts with the aldehydes *p*Ph or BiPh to form [1 + 1] intermediates; the [1 + 1] intermediates then convert into their homoleptic macrocycles, *p*Ph_2_(NON)_2_ and BiPh_2_(NON)_2_, *via* both homoleptic cyclization following three possible pathways (Scheme 2) and self-correction pathways (Scheme 4). Due to the competition between *p*Ph and BiPh containing species, two heteroleptic intermediates, namely [*p*Ph + BiPh + NON] and [*p*Ph + BiPh + 2NON], may be formed and lead to the final homoleptic products by both dissociation and self-correction processes (Scheme 4).^13^

### Self-correction in the self-sorting processes leading to the formation of homoleptic macrobicyclic cages

**Fig. 5:**
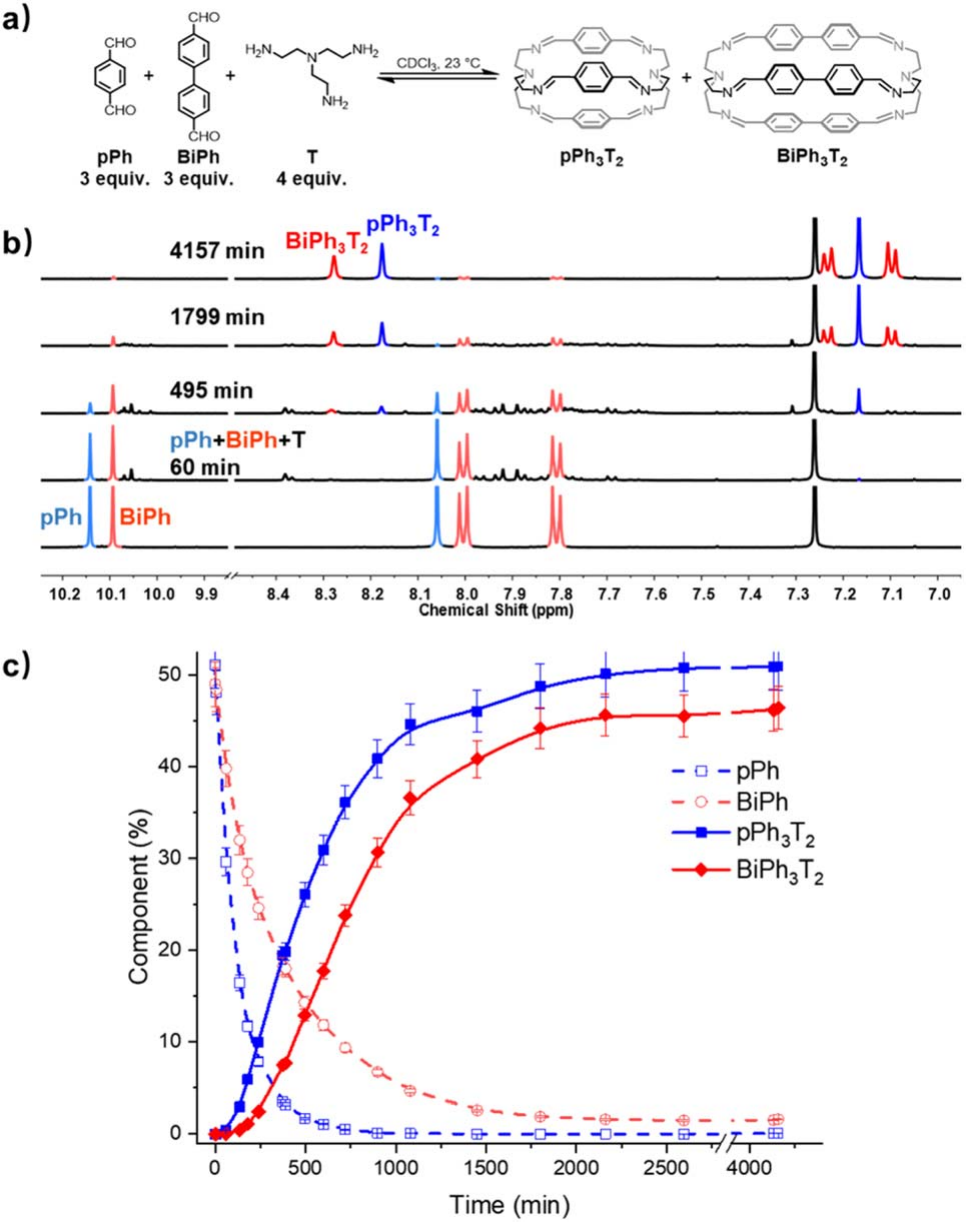
(a) Component self-sorting in the competitive formation of two macrobicyclic cages *p*Ph_3_T_2_, BiPh_3_T_2_. (b) Evolution of the partial ^1^H NMR spectra (500 MHz, CDCl_3_, 23 °C) of the reaction 3*p*Ph + 3Biph + 4T ([*p*Ph]_0_ = [BiPh]_0_ = 3.6 mM, [T]_0_ = 4.8 mM) over 4157 min. The bottom trace corresponds to the dialdehyde components, *p*Ph and BiPh. (c) ^1^H NMR monitoring of dialdehydes *p*Ph, BiPh and cages *p*Ph_3_T_2_, BiPh_3_T_2_ over 4157 min. The distribution (%) have been obtained by the integration of the of the aromatic regions and aldehyde CHO proton signals in the 500 MHz ^1^H NMR spectra. Error in ^1^H-NMR signal integration: ±5%. The corresponding *t*_1/2_ values are given in the ESI.†

A three-component homo-self-sorting experiment resulting in the parallel formation of two cages was set up using two different bridging components *p*Ph : BiPh : T in a 3 : 3 : 4 ratio. Fig. 5 shows that after mixing the components, the concentration of *p*Ph rapidly decreased. Simultaneously broad new signals appeared which progressively disappeared as the reaction proceeded, indicating that they were due to intermediate species. After about 60 min, new small peaks appeared, the signals of the intermediates previously formed were attenuated and an intense imine peak corresponding to the small cage *p*Ph_3_T_2_ arose at 8.17 ppm. Shortly after, the imine signal of the larger cage BiPh_3_T_2_ appeared, while both *p*Ph and BiPh were progressively consumed. The aldehyde *p*Ph was nearly fully consumed after 720 min, whereas 9% of BiPh remained unreacted. The consumption sequence was in accordance with their corresponding cage formation rates. Full conversion into 51% of *p*Ph_3_T_2_ and 46% of BiPh_3_T_2_ as well as 2% of unreacted BiPh was attained after about 4157 min (69 h).§ The final spectrum indicated the high-fidelity of self-sorting and confirmed the absence of any heteroleptic structures. Comparing these observations to the results obtained for separate formation, both the consumption of BiPh and formation of BiPh_3_T_2_ were accelerated in the self-sorting experiment with respect to their separate formation, presumably because of the higher initial concentration of T, analogous to the observations in the macrocycle self-sorting experiment. In contrast the rate of formation of *p*Ph_3_T_2_ remained the same compared as in the case of separate formation, indicating the probable involvement of correction and dissociation processes of heteroleptic intermediates (for more kinetic data see ESI Fig. S46†).

**Fig. 6:**
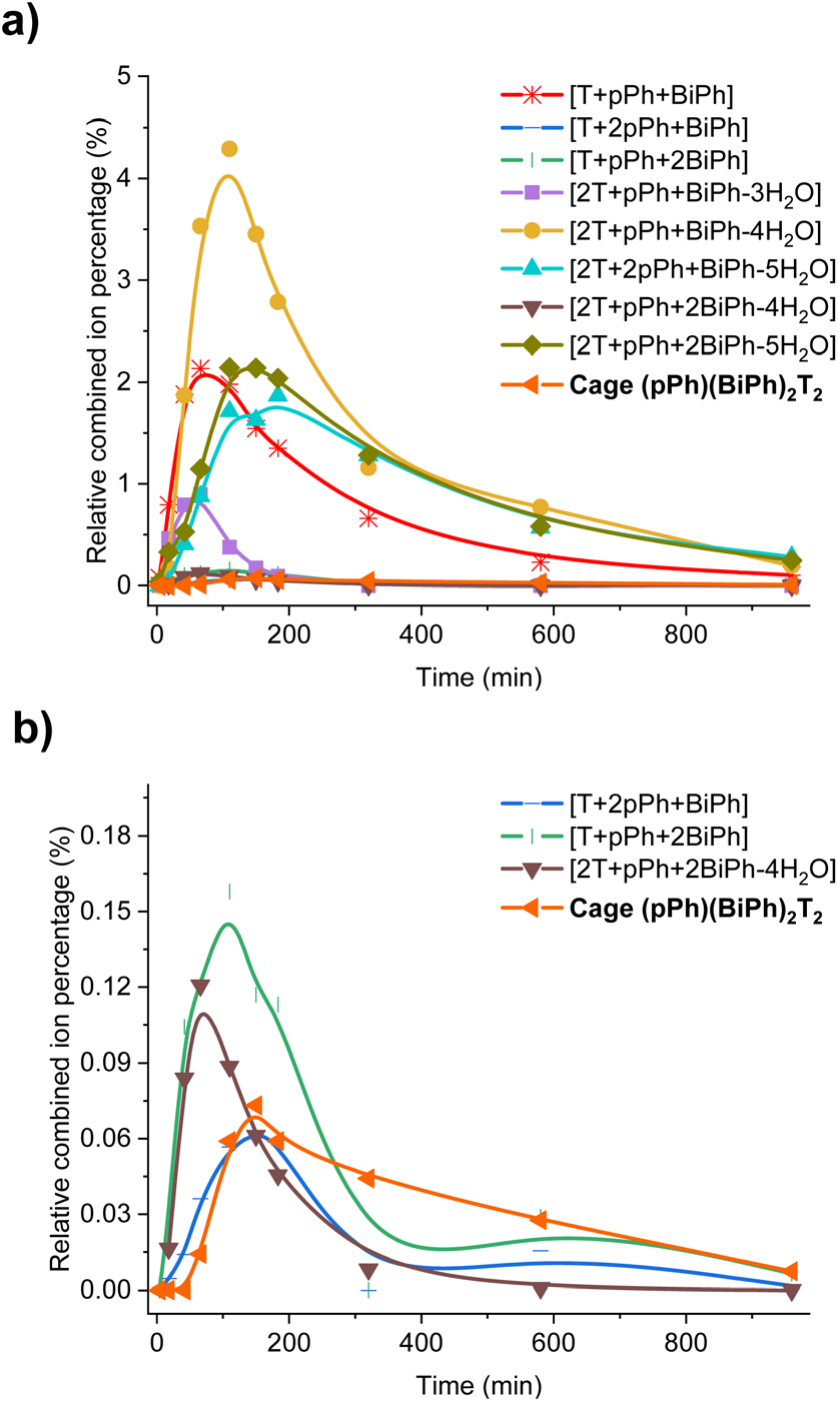
Evolution of the HRMS spectra displaying the self-correction process in the course of the self-assembly. (a) HRMS-ESI monitoring of the evolution of the heteroleptic intermediates generated from the 3*p*Ph + 3BiPh + 4T ([*p*Ph]_0_ = [BiPh]_0_ = 2 mM, T = 2.7 mM) library in the course of the homo-self-sorting process leading to the homoleptic structures *p*Ph_3_T_2_, BiPh_3_T_2_. (b) Amplified view along the *y* axis. NB: the relative combined ion percentage is obtained by the ratio of combined ion count of each species to the sum of combined ion counts for all species at each time point. These data do not provide quantitative information about the relative amounts of each species identified by its mass, but, taken separately, they display the evolution of a given identified species during the course of the reaction. See Fig. S50† for the evolution of all species generated during the reaction course.

In order to gain some more information about the mechanism of the macrobicycle self-sorting processes, the key intermediates formed during the reaction were identified by their mass from the full HRMS spectra (Fig. S49 and S50†). Table S6† lists all identified intermediates and cages observed from time dependent HRMS experiments. The full evolution of the spectra over time suggests qualitative trends for these species. The reaction of *p*Ph : BiPh with T started by the rapid appearance of [*p*Ph + T] and [BiPh + T] intermediates. Thereafter several heteroleptic intermediates formed. All these intermediates progressively disappeared resulting in the simultaneous formation of the homoleptic cages *p*Ph_3_T_2_ and BiPh_3_T_2_. Close inspection of the HRMS plot (under high amplification, Fig. 6b) revealed a decrease in concentration of the heteroleptic cage (*p*Ph)(Biph)_2_T_2_ after its formation, indicating the operation of a self-correction process. Support for the general occurrence of such self-correction was obtained by investigation of a sequential cage formation experiment.

A ^1^H-NMR study indicated that, in a solution (with hexamethyldisilane as internal standard) prepared by mixing 3 equiv. BiPh and 4 equiv. T, 36% of the initial T was converted into the cage BiPh_3_T_2_ after 48 h. As no CHO signal of free BiPh remained, the rest of BiPh appeared to have formed [BiPh + 2T] (10% with respect to T) and [2BiPh + 2T] (15% with respect to T), accompanied by the remaining free T (40%). This composition did not change over 3 more days. Then, on addition of 3 equiv. *p*Ph to this solution, all intermediates and free T were converted into >47% (with respect to the internal reference) of each of the two macrobicycles *p*Ph_3_T_2_ and BiPh_3_T_2_ (see ESI, Fig. S51†). These results indicated that the reaction between (i) the added *p*Ph, (ii) the [BiPh + 2T] and [2BiPh + 2T] intermediates, and (iii) the remaining free T gave the *p*Ph_3_T_2_ cage together with the additional BiPh_3_T_2_ cage. Taken together, they confirm that self-correction did indeed take place by component recombination driven by the formation of the *p*Ph_3_T_2_ cage.

**Scheme 5:**
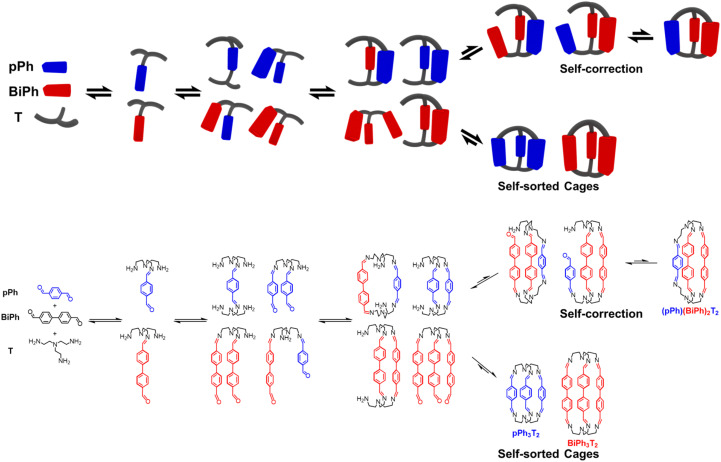
Schematic (top) and molecular (bottom) representations of the stepwise self-sorting process showing the most abundant intermediates on the way to the formation of the imine-based dynamic covalent homoleptic macrobicyclic structures *p*Ph_3_T_2_ and BiPh_3_T_2_.

These data define a progressive evolution towards the final homoleptic structures along a sequence of steps (Scheme 5) which may involve up to 38 intermediates (see ESI Fig. S52†): the [1 + 1] intermediate forms initially in high abundance, followed by the three-component intermediates [1 + 2] and [2 + 1] which first increase and then slowly decrease to a plateau; thereafter, the four-component [2 + 2], [1 + 3] and the five-component [2 + 3] larger intermediates increase in abundance before declining to give the final homoleptic cage structures. The eventual formation of transient oligomeric intermediates has not been taken into account. The results indicate that self-correction of the heteroleptic species must take place at the different steps along the reaction sequence up to the final processing into the pure homoleptic macrobicyclic structures *p*Ph_3_T_2_ and BiPh_3_T_2_.

### DFT calculations elucidating the homo-self-sorting driving forces in macrocyclic and macrobicyclic systems

**Fig. 7:**
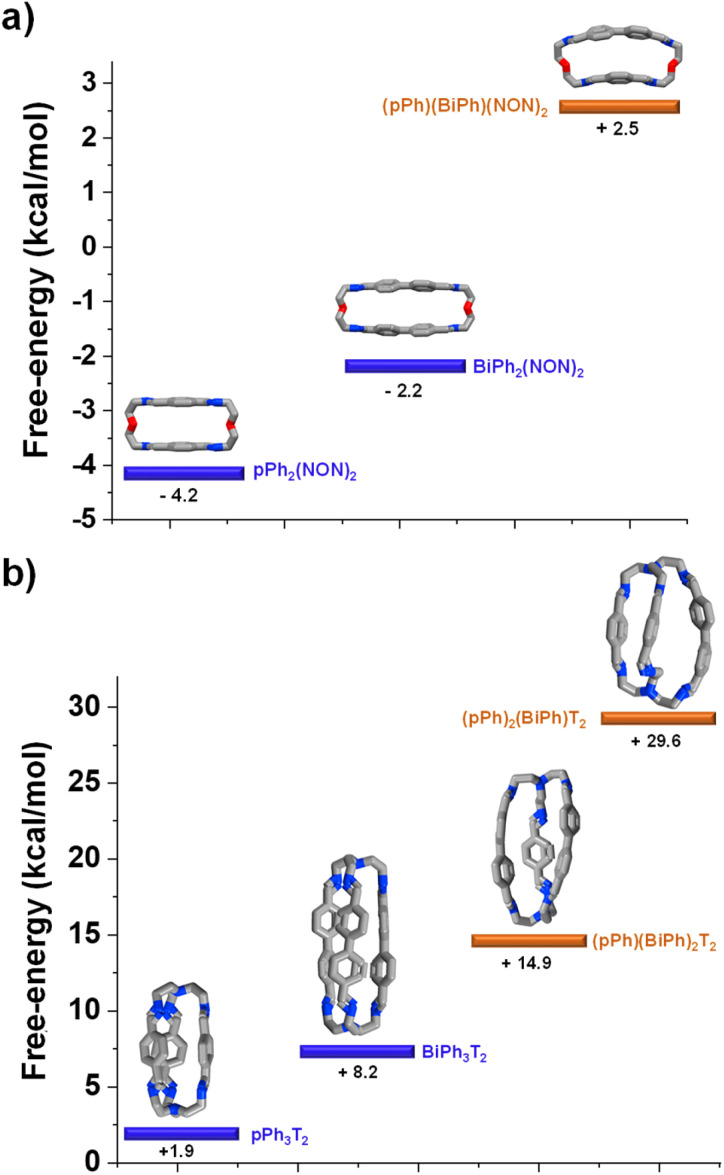
Most stable conformation (DFT, B3LYP, 6-311+G(d,p), Gaussian 09) for the homoleptic and heteroleptic (a) macrocycles and (b) cryptands. Free energies (kcal mol^−1^) have been calculated from the formation energies of the reaction components. See DFT calculations section (Section 7.4) in ESI† for more details.

DFT calculations for the homoleptic and heteroleptic species of the two-cyclic order systems were performed to shed light on the contributions of the forces leading to the observed homo-self-sorting. Firstly, the macrocyclic structures were considered. The most stable conformations calculated corroborate the expected *D*_2h_ symmetry for both *p*Ph_2_(NON)_2_ and BiPh(NON)_2_ (Fig. 7, see also Fig. S53†). On the other hand, the heteroleptic macrocycle (*p*Ph)(BiPh)(NON)_2_ presents a *C*_2_-symmetric structure. This lower symmetry (number of microstates: 2 for *C*_2_*vs.* 4 for *D*_2h_; Fig. S53†) seems to be decreasing the thermodynamic stability of (*p*Ph)(BiPh)(NON)_2_ due to entropic implications.^14^ Moreover, the cyclic structure of the heteroleptic species adopts a quite arched conformation that could further destabilize this constituent, as evidenced by the higher energy calculated in comparison with the ones for the homoleptic macrocycles (+2.5 kcal mol^−1^ for (*p*Ph)(BiPh)(NON)_2_, Fig. 7a). A similar scenario was found for the macrobicyclic system. Interestingly, in this case, the difference in symmetry numbers between the homoleptic and heteroleptic species is even more pronounced, as *p*Ph_3_T_2_ and BiPh_3_T_2_ present a *D*_3h_ symmetry (number of microstates: 12) but the heteroleptic cages (*p*Ph)(BiPh)_2_T_2_ and (*p*Ph)_2_(BiPh)T_2_ are *C*_2_-symmetric species (number of microstates: 2) (Fig. S54†). One might thus consider that the macrobicyclic system should undergo a more efficient homo-self-sorting considering the energy difference (heteroleptic–homoleptic) calculated for the two systems (Fig. 7 and S55†), with values increasing following the order: (*p*Ph)(BiPh)(NON)_2_ < (*p*Ph)(BiPh)_2_T_2_ ≪ (*p*Ph)_2_(BiPh)T_2_. This hypothesis was corroborated by HRMS analyses. Whilst the heteroleptic macrocycle (*p*Ph)(BiPh)(NON)_2_ was formed in *ca.* 1% relative abundance after 500 min, only minor traces of the heteroleptic molecular cage (*p*Ph)(BiPh)_2_T_2_ were observed (max. abundance of *ca.* 0.1% after 180 min), with cage (*p*Ph)_2_(BiPh)T_2_ not being detected during the analyses (Fig. 6b and S50 in ESI†).¶¶We have also calculated the relative intensities of the metastable heteroleptic macrocycles and macrobicycles in view of the analogous homoleptic compounds, as the HRMS comparison will be more representative due to similar response for each set of constituents. In this case, the maximum relative abundance for (*p*Ph)(BiPh)(NON)_2_ was of *ca.* 7% and for (*p*Ph)(BiPh)_2_T_2_ was of *ca.* 0.3% (Tables S6 and S8 in ESI†).

### Homo-self sorting from [3 × 1] four-component DC_ov_Ls

**Fig. 8:**
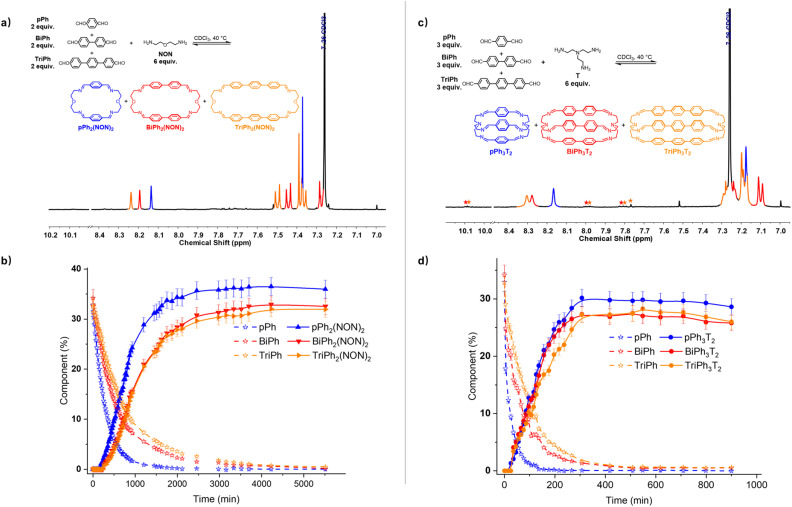
(a) Partial ^1^H NMR spectrum (400 MHz, CDCl_3_) of the parallel formation of a mixture of macrocycles *p*Ph_2_(NON)_2_, BiPh_2_(NON)_2_ and TriPh_2_(NON)_2_ through self-sorting of 2*p*Ph + 2BiPh + 2TriPh + 6NON after 5510 min at 40 °C. (b) ^1^H NMR monitoring of the formation of the macrocycles *p*Ph_2_(NON)_2_, BiPh_2_(NON)_2_ and TriPh_2_(NON)_2_ over 5510 min. The initial concentration of each dialdehyde was 1.0 mM. (c) Partial ^1^H NMR spectrum (400 MHz, CDCl_3_) of the parallel formation of macrobicyclic cages *p*Ph_3_T_2_, BiPh_3_T_2_ and TriPh_3_T_2_ though self-sorting of 3*p*Ph + 3BiPh + 3TriPh + 6T after 900 min at 40 °C. (d) ^1^H NMR monitoring of the formation of the cages *p*Ph_3_T_2_, BiPh_3_T_2_ and TriPh_3_T_2_ over 900 min. The initial concentration of each dialdehyde was 1.0 mM. Error in ^1^H-NMR signal integration: ±5%. The spectra and kinetic curves are coloured in accordance with the macrocycles and cages. The signals of free dialdehydes BiPh and TriPh are indicated by red and orange stars, respectively.

A four-component DC_ov_L composed of *p*Ph, BiPh, TriPh and NON in 2 : 2 : 2 : 6 ratio was investigated in CDCl_3_ (Fig. 8a and b). The ^1^H NMR monitoring indicated that the reaction equilibrium was reached after 5510 min of heating at 40 °C. After the addition of NON to the mixture of dialdehydes, the concentration of the four components declined as that of the macrocycles rose to be the only species present at equilibrium. The generation of macrocycles followed the rate sequence *p*Ph_2_(NON)_2_ > BiPh_2_(NON)_2_ ≥ TriPh_2_(NON)_2_. The composition of the final equilibrium solution was 36% *p*Ph_2_(NON)_2_, 32% BiPh_2_(NON)_2_ and 32% TriPh_2_(NON)_2_.§ The ultimate formation of the three macrocycles was also confirmed by HRMS (ESI, Fig. S57†). The sum of all the constituents present in the equilibrium state agreed well with a high-fidelity self-sorting of the three competing macrocycles.

In another case, a four-component DC_ov_L composed of *p*Ph, BiPh, TriPh and T in a 3 : 3 : 3 : 6 ratio was investigated in CDCl_3_ (Fig. 8c and d). The evolution was monitored by ^1^H NMR at 40 °C. Again, the reactant concentrations decreased uniformly with time as signals belonging to cages *p*Ph_3_T_2_, BiPh_3_T_2_ and TriPh_3_T_2_ progressively rose following the sequence of relative rates *p*Ph_3_T_2_ ≥ BiPh_3_T_2_ ≥ TriPh_3_T_2_. After 900 min, signals corresponding to macrobicyclic cages *p*Ph_3_T_2_, BiPh_3_T_2_ and TriPh_3_T_2_ were clearly apparent (as also confirmed by HRMS, ESI, Fig. S59†) and the distribution was 29% *p*Ph_3_T_2_, 26% BiPh_3_T_2_, 26% TriPh_3_T_2_ as well as <1% unreacted BiPh and <1% unreacted TriPh.§ Despite the poor solubility of cage TriPh_3_T_2_, the system was still able to self-sort towards the homoleptic macrocycles, but the final molar balance was of *ca.* 83%, suggesting that very small amounts of insoluble species might have been formed.

These results, taken together, indicate that self-sorting systems of higher diversity can be set up by appropriate choice of precursor components.

### Homo-self sorting from [3 × 2] five-component and [4 × 2] six-component DC_ov_Ls and networks

**Fig. 9:**
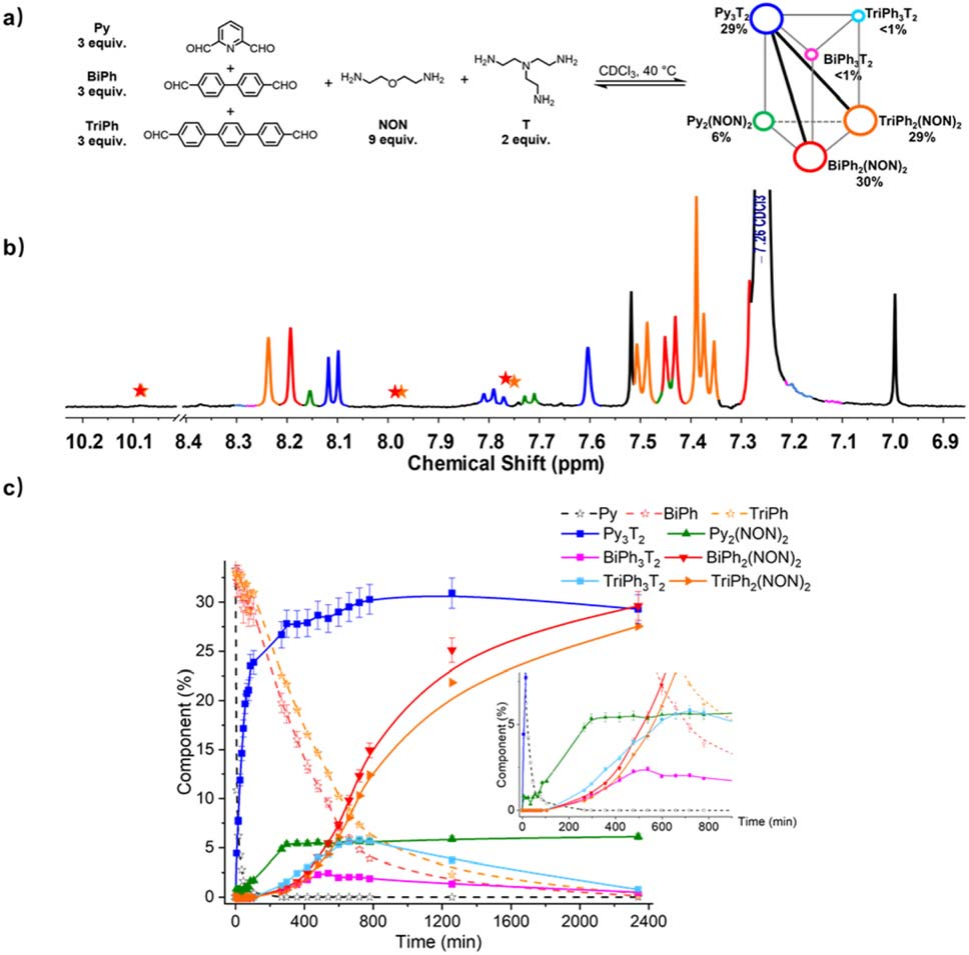
(a) [3 × 2] trigonal prismatic DC_ov_L generated from 3Py + 3BiPh + 3TriPh + 2T + 6NON; (b) partial ^1^H NMR spectrum (400 MHz, CDCl_3_) of the equilibrated reaction mixture; (c) ^1^H NMR monitoring of macrocycles and macrocyclic cages over 2340 min. The initial concentration of each dialdehyde was 1.0 mM. Error in ^1^H-NMR signal integration: ±5%. The signals of free dialdehydes BiPh and TriPh are indicated by red and orange stars, respectively. The spectra and kinetic curves are coloured in accordance with the molecular structures.

To evaluate the self-sorting behaviour on increasing the complexity and diversity two systems containing a larger number of components were investigated. A first system [3 × 2] involved five components (three dialdehydes of increasing lengths, Py, BiPh and TriPh) together with the two polyamines of linear NON and branched T types with stoichiometries 3 : 3 : 3 : 6 : 2 for the [3 × 2] (Fig. 9). The resulting mixture was heated at 40 °C for up to 2340 min and the evolution of its composition was followed by ^1^H NMR spectroscopy. The affinity of Py for T was high enough to allow for selective formation of cage Py_3_T_2_. Indeed, after 90 min, all of the Py component had transformed into 23% of the anticipated cage Py_3_T_2_ and 2% of macrocycle Py_2_(NON)_2_. Meanwhile, approximately 33% of BiPh and 31% of TriPh (or a little less as some intermediates also contain –CHO groups whose signals might overlap with –CHO signals in BiPh and TriPh) remained unreacted, and no cages and macrocycles belonging to BiPh and TriPh were detected. After 720 min, no Py could be detected and the composition of the mixture was, BiPh (<5%), TriPh (<7%), Py_3_T_2_ (30%), Py_2_(NON)_2_ (6%), BiPh_3_T_2_ (2%), BiPh_2_(NON)_2_ (12%), TriPh_3_T_2_ (<6%), and TriPh_2_(NON)_2_ (10%), the remainder being in the form of unassigned intermediates. After 2340 min, the distribution was BiPh (<1%), TriPh (<1%), Py_3_T_2_ (29%), Py_2_(NON)_2_ (6%), BiPh_3_T_2_ (<1%), BiPh_2_(NON)_2_ (30%), TriPh_3_T_2_ (<1%), TriPh_2_(NON)_2_ (28%).§ The final distribution showed an almost completely sorted outcome (molar sum of constituents: 96%) with the cage Py_3_T_2_ and its agonistic macrocycles BiPh_2_(NON)_2_ and TriPh_2_(NON)_2_ as predominant compounds in the mixture.

**Fig. 10:**
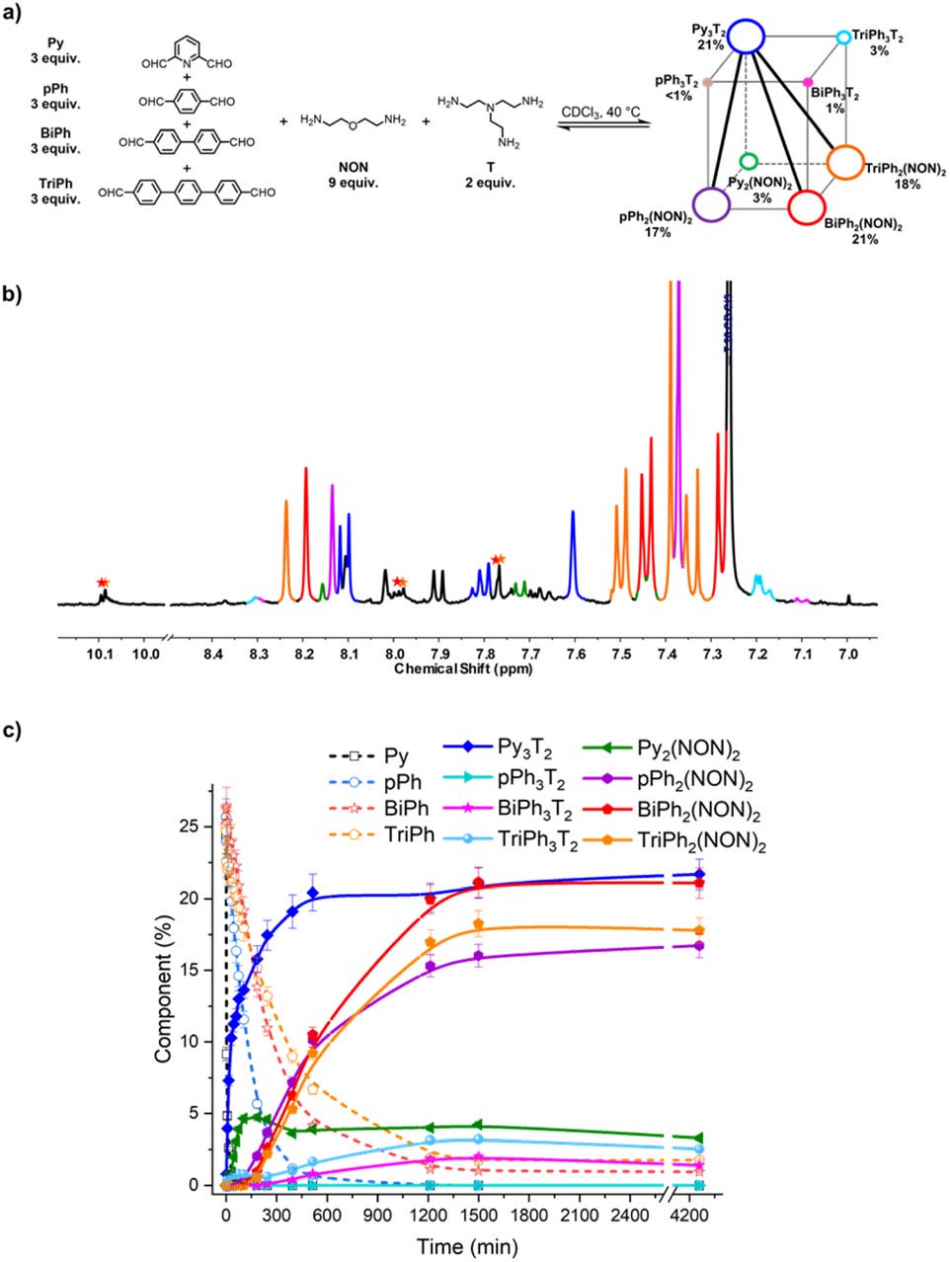
(a) [4 × 2] square prismatic DC_ov_L generated from 3Py + 3*p*Ph + 3BiPh + 3TriPh + 2T + 9NON; (b) partial ^1^H NMR spectrum (400 MHz, CDCl_3_, 40 °C) of the equilibrated reaction mixture; (c) ^1^H NMR monitoring of macrocycles and macrocyclic cages over 4260 min. The initial concentration of each dialdehyde was 2.0 mM. The signals of free dialdehydes BiPh and TriPh are indicated by red and orange stars, respectively. The larger size and the bold diagonal lines indicate the selective cogeneration of agonistic constituents. The spectra and kinetic curves are coloured in accordance with the molecular structures.

The second system concerned the self-sorting behaviour of [4 × 2] six-component DC_ov_L built up from four dialdehydes Py, *p*Ph, BiPh and TriPh and two diamines NON and T in 3 : 3 : 3 : 3 : 9 : 2 ratio. It was studied by NMR and HRMS at 40 °C for 3 days (Fig. 9). The equilibrated solution gave BiPh (1%), TriPh (2%), Py_3_T_2_ (21%), Py_2_(NON)_2_ (3%), *p*Ph_3_T_2_ (<1%), *p*Ph_2_(NON)_2_ (17%), BiPh_3_T_2_ (1%), BiPh_2_(NON)_2_ (21%), TriPh_3_T_2_ (3%), and TriPh_2_(NON)_2_ (18%).§ These products contained approximately 88% of the initial reactants. The final NMR spectrum inferred a less efficient self-sorting in this more complex DC_ov_L, as evidenced by the unassigned signals in Fig. 10b. Such undesired products could be acting as kinetic traps that preclude an entirely sorted outcome. One might note, however, starting with six components generated only four dominant self-sorted architectures out of the eight possible homoleptic products. These results further corroborated the proposed important role of structural features towards achieving self-sorting of DC_ov_Ls. Nonetheless, it should be noted that the system is biased by the fast and dominant formation of the Py_3_T_2_ cage which precludes the formation of the other two cages as there is no T left (see Fig. S14 and S33† for kinetic profiles of formation of Py_2_NON_2_ and Py_3_T_2_ alone, and Fig. S35† for the competition between these three components). It again verifies the conclusion that, especially on introduction of a prevalent competitor, an increased compositional complexity may lead to a simplified output through a process of competitive self-sorting.^15^

Finally, the two DC_ov_Ls [3 × 2] and [4 × 2] can be represented by constitutional dynamic networks (CDNs)^16^ forming respectively a trigonal prism (Fig. 9a) and a square prism (Fig. 10a). The edges link antagonistic constituents that share a component and the diagonals share agonistic constituents not sharing a component.

## Conclusions

Delineating the reaction pathways followed in multiple component DC_ov_Ls is crucial for the rational design of self-sorting systems of high diversity and complexity capable of generating several outputs side-by-side. The present study of the temporal evolution of imine-based dynamic covalent libraries and of the corresponding self-sorting processes involved both the separate and the concomitant formation of two types of representative structures of different cyclic order,^1^ macrocycles and macrobicyclic cages. The results suggest a rather complicated, multistep and multibranched pathway through consecutive reactions and numerous intermediates. Upon mixing the components, kinetically determined assembly first occurs to form a wide range of intermediates, some of which are then transformed into metastable heteroleptic structures. These metastable species then undergo dissociation and reassociation through component exchange to give homoleptic intermediates. The latter slowly converted to the final macrocycles and macrocyclic cages through pathways involving self-correction processes, while a small number of intermediates remained in the final product. The results highlight the important role played by structural features in controlling both the kinetic and thermodynamic parameters to balance a sorted *versus* a scrambled output. Besides, the DFT and HRMS outcomes infer the role of entropy in the homo-self-sorting attained. One may anticipate that they will contribute to the rational design of higher order self-sorting systems of increased complexity and diversity as well as inspire chemists towards the generation of novel dynamic covalent architectures and the investigation of the dynamic networks underlying self-sorting processes.

## Data availability

The ESI contains experimental details, characterization, spectral data, computational data and cartesian coordinates. CCDC 2006765, 2006766 and 2018494 contain the supplementary crystallographic data for this paper.†

## Author contributions

Z. Y. and J.-M. L. conceptualized the project; Z. Y., and C. A. performed the NMR and HRMS experiments; Z. Y. analysed the data; F. E. performed the DFT calculations; Z. Y. and F. E. wrote the original draft; all authors revised the manuscript; J.-M. L. supervised the project.

## Conflicts of interest

There are no conflicts to declare.

## Supplementary Material

SC-014-D3SC01174G-s001

SC-014-D3SC01174G-s002

## References

[cit1] (f) Constitutional Dynamic Chemistry, Topics in Current Chemistry, ed. M. Barboiu, Springer Berlin, Heidelberg, 2012, vol. 322

[cit2] Jazwinski J., Lehn J.-M., Lilienbaum D., Ziessel R., Guilhem J., Pascard C. (1987). J. Chem. Soc., Chem. Commun..

[cit3] Lehn J.-M. (1985). Science.

[cit4] Hosseini M. W., Lehn J.-M., Mertes M. P. (1983). Helv. Chim. Acta.

[cit5] (e) Ghosh S. and Isaacs L., Dynamic Combinatorial Chemistry: In Drug Discovery, Bioorganic Chemistry, and Materials Science, ed. B. L. Miller, Wiley-VCH, Hoboken, 2010, pp. 118–154

[cit6] Acharyya K., Mukherjee S., Mukherjee P. S. (2013). J. Am. Chem. Soc..

[cit7] Klotzbach S., Beuerle F. (2015). Angew. Chem., Int. Ed..

[cit8] Jiang S., Jones J. T. A., Hasell T., Blythe C. E., Adams D. J., Trewin A., Cooper A. I. (2011). Nat. Commun..

[cit9] Kołodziejski M., Stefankiewicz A. R., Lehn J.-M. (2019). Chem. Sci..

[cit10] Krässig V. H., Greber G. (1955). Makromol. Chem..

[cit11] Drew M. G. B., Yates P. C., Murphy B. P., Nelson J., Nelson S. M. (1986). Inorg. Chim. Acta.

[cit12] Drew M. G. B., McDowell D., Nelson J. (1988). Polyhedron.

[cit13] Jiang W., Schäfer A., Mohr P. C., Schalley C. A. (2010). J. Am. Chem. Soc..

[cit14] Lin S.-K. (1996). J. Chem. Inf. Comput. Sci..

[cit15] Dhers S., Holub J., Lehn J.-M. (2017). Chem. Sci..

[cit16] Lehn J.-M. (2013). Angew. Chem., Int. Ed..

