# Supplementary material for: Dynamic covalent self-assembly and self-sorting processes in the formation of imine-based macrocycles and macrobicyclic cages
Source: Chem Sci. 2023 Jun 2;14(24):6631–42. doi: 10.1039/d3sc01174g (PMC10284075; doi:10.1039/d3sc01174g)
Supplement: SC-014-D3SC01174G-s001 [file SC-014-D3SC01174G-s001.pdf]

Electronic Supplementary Material (ESI) for Chemical Science.
This journal is © The Royal Society of Chemistry 2023

SUPPORTING INFORMATION

Dynamic Covalent Self-Assembly and Self-Sorting Processes in the Formation of Imine-based Macrocycles and Macrobicyclic Cages

Zhaozheng Yang, [a,b] Ferran Esteve [b], Cyril Antheaume [b] and Jean-Marie Lehn *[a,b]

[a.] Lehn Institute of Functional Materials (LIFM), Sun Yat-Sen University, 510006 Guangzhou, China.

[b.] Laboratoire de Chimie Supramoléculaire, Institut de Science et d'Ingénierie Supramoléculaires (ISIS), Université de Strasbourg, 8 allée Gaspard Monge, 67000 Strasbourg, France. E-mail: lehn@unistra.fr

Table of Contents

1. Instrumentation and Measurement 2
2. List of the selected dialdehydes, polyamines, macrocycles and macrobicyclic cages 3
3. Experimental Method 3
3.1 Experimental methods for time-dependent ^{1}H NMR monitoring studies 3
3.2 Experimental methods for time-dependent HRMS monitoring studies 5
3.3 NOTE 5
4. Synthesis and Characterization 5
4.1 Synthesis of [1,1':4',1''-Terphenyl]-4,4''-dicarboxaldehyde (TriPh) 5
4.2 Synthesis of macrocycle $TriPh_2(NON)_2$ 6
4.3. Synthesis of Macrobicyclic Cage $TriPh_3T_2$ 7
5. Formation of isolated macrocycles 9
5.1 Formation of macrocycle $pPh_2(NON)_2$ 9
5.2 Formation of macrocycle $BiPh_2(NON)_2$ 11
5.3 Formation of macrocycle $Py_2(NON)_2$ 13
5.4 Time-dependent HRMS of macrocycle $pPh_2(NON)_2$ 14
5.5 Time-dependent HRMS of macrocycle $BiPh_2(NON)_2$ 15
6. Formation of isolated macrobicyclic cages 17
6.1 Time-dependent ^{1}H NMR of macrobicyclic cage pPh_3T_2 17
6.2 Time-dependent ^{1}H NMR of macrobicyclic cage $BiPh_3T_2$ 20
6.3 Time-dependent ^{1}H NMR of macrobicyclic cage Py_3T_2 24
6.4 Competitive experiment of 3Py + 3NON + 2T 25
6.5 Time-dependent HRMS of macrobicyclic cage pPh_3T_2 26
6.6 Time-dependent HRMS of macrobicyclic cage $BiPh_3T_2$ 28
7. Self-sorting experiments 30
7.1 Self-sorting experiment 2pPh + 2BiPh + 4NON 30
7.2 Self-sorting experiment 3pPh + 3BiPh + 4T 35
7.3 Stepwise self-sorting experiment with BiPh, T and pPh. 41
7.4 DFT calculations 42
7.5 Self-sorting experiment 2pPh + 2BiPh + 2TriPh + 6NON 45
7.6 Self-sorting experiment 3pPh + 3BiPh + 3TriPh + 6T 46
7.7 Self-sorting experiment 3Py + 3BiPh + 3TriPh + 2T + 6NON 47
7.8 Self-sorting experiment 3Py + 3pPh + 3BiPh + 3TriPh + 2T + 9NON 48
8. X-ray crystallography 49
9. References 53
10. Cartesian coordinates and energies 55

1. Instrumentation and Measurement

NMR spectra were recorded on Bruker Avance 400 (400 MHz for ^{1}H and 100 MHz for ^{13}C), Bruker Avance III plus 400 (400 MHz for ^{1}H) and Bruker Ascend Spectroscope Avance Neo-500 MHz (500 MHz for ^{1}H and 125 MHz for ^{13}C). MestReNova 10 software was used for the treatment of the NMR spectra. Chemical shifts are given in ppm. Residual solvent peaks were taken as reference ($CDCl_3$:7.26 ppm for ^{1}H and 77.16 ppm for ^{13}C). The quantitative ^{1}H NMR was measured by using hexamethyldisilane as internal standard. The error in ^{1}H-NMR integration amounts to about 5%. Peaks are described as singlet (s), doublet (d). Unless otherwise noted, spectra were recorded at 23 °C.

HRMS-ESI (High-Resolution Mass Spectrometry-Electro-Spray Ionisation) mass spectra were recorded by direct injection into a ThermoFisher Exactive Plus EMR Orbitrap mass spectrometer.

X-Ray diffraction data was obtained on a PHOTON-III CPAD (Bruker) equipped with a CCD detector. The structures were solved with the SHELXT 2014/5 structure solution program and refined with the SHELXL-2014/7 refinement package.[1] Artwork representations were processed using MERCURY software.[2]

X-Ray diffraction data collection was carried out on a Bruker PHOTON-III DUO Kappa CPAD diffractometer.

Commercially available chemicals were generally purchased from Sigma-Aldrich, Alfa Aesar, Fluorochem, TCI and were used without further purification. Solvents and reagent of pharmaceutical grade quality were purchased from Carlo Erba, and solvents of spectroscopic grade were purchased from Sigma-Aldrich and Fisher Chemical. $CDCl_3$ was purchased from Euriso-TOP and filtered through basic alumina to remove traces of acidity before use.

2. List of the selected dialdehydes, polyamines, macrocycles and macrobicyclic cages

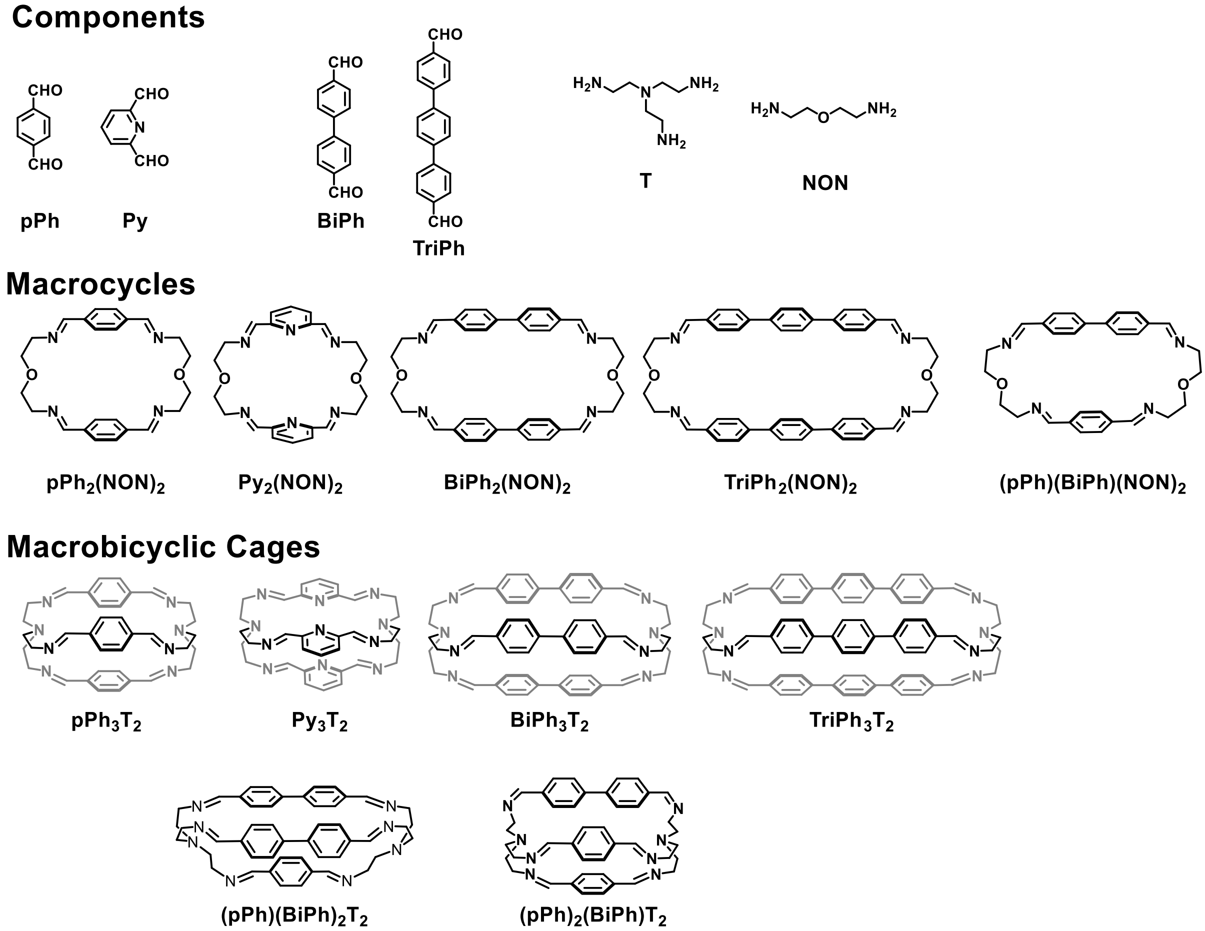

Scheme S1. All of aldehydes, amines and macrocycles, macrobicyclic cages that have been used in the text.

3. Experimental Method

3.1 Experimental methods for time-dependent ^{1}H NMR monitoring studies

a) Preparation of stock solutions

$CDCl_3$ was filtered through basic alumina oxide to remove traces of acidity before use. A stock solution of internal standard was made by adding 1.5 μL hexamethyldisilane into 2 mL $CDCl_3$. The stock solutions of amines (**NON**, **T**) and dialdehydes were freshly prepared in $CDCl_3$ and quantified referencing to the internal standard (hexamethyldisilane). For all the cases, dialdehyde and polyamine were mixed in a proper stoichiometric ratio in $CDCl_3$ containing hexamethyldisilane as internal standard. The operation details were described as following:

b) formation of a single macrocycle

In an NMR tube, 30 mM solution of **NON** 72 μL (2 equiv.) was added into 528 μL $CDCl_3$ solution containing 2 equiv. dialdehyde and 40 μL internal standard solution. The final concentration of dialdehyde and **NON** was

3.6 mM. The reaction solution was monitored over time at 23-25 °C, until the equilibrium was reached. The composition of species was calculated according to the internal standard and presented on basis of imine integration data.

c) formation of a single macrobicyclic cage

In an NMR tube, 30 mM solution of **T** 48 µL (2 equiv.) was added into 552 µL $CDCl_3$ solution containing 3 equiv. dialdehyde (for instance **pPh**, **Py**, **BiPh**) and 40 µL internal standard solution. The final concentration of dialdehyde and **T** was 3.6 mM and 2.4 mM, respectively. The reaction solution was monitored over time at 23-25 °C, until the equilibrium was reached. The composition of species was calculated according to the internal standard and presented on basis of imine integration data.

d) self-sorting of 2pPh+2BiPh+4NON

In an NMR tube, 30 mM solution of **NON** 156 µL (4 equiv.) was added into 494 µL $CDCl_3$ solution containing 2 equiv. dialdehyde **pPh**, 2 equiv. dialdehyde **BiPh** and 40 µL internal standard solution. The final concentration of each dialdehydes and **NON** were 3.6 mM and 7.2 mM, respectively. The reaction solution was monitored over time at 23-25 °C, until the equilibrium was reached. The composition of species was calculated according to the internal standard and presented on basis of imine integration data.

e) self-sorting of 3pPh+3BiPh+4T

In an NMR tube, 30 mM solution of **T** 96 µL (4 equiv.) was added into 504 µL $CDCl_3$ solution containing 3 equiv. dialdehyde **pPh**, 3 equiv. dialdehyde **BiPh** and 40 µL internal standard solution. The final concentration of each dialdehydes and **T** were 3.6 mM and 4.8 mM, respectively. The reaction solution was monitored over time at 23-25 °C, until the equilibrium was reached. The composition of species was calculated according to the internal standard and presented on basis of imine integration data.

f) self-sorting of 2pPh+2BiPh+2TriPh+6NON

In an NMR tube, 30 mM solution of **NON** 65 µL (6 equiv.) was added into 585 µL $CDCl_3$ solution containing dialdehyde **pPh**, **BiPh**, **TriPh** (2 equiv. of each) and 40 µL internal standard solution. The final concentration of each dialdehydes were 1.0 mM and **NON** was 3.0 mM. The reaction solution was monitored over time at 40 °C, until the equilibrium was reached. The composition of species was calculated according to the internal standard and presented on basis of imine integration data.

h) self-sorting of 3pPh+3BiPh+3TriPh+6T

In an NMR tube, 30 mM solution of **T** 43 µL (6 equiv.) was added into 607 µL $CDCl_3$ solution containing dialdehyde **pPh**, **BiPh**, **TriPh** (3 equiv. of each) and 40 µL internal standard solution. The final concentration of each dialdehydes and **T** were 1.0 mM and 2.0 mM, respectively. The reaction solution was monitored over time at 40 °C, until the equilibrium was reached. The composition of species was calculated according to the internal standard and presented on basis of imine integration data.

i) self-sorting of 3pPh+3BiPh+3TriPh+2T+6NON

In an NMR tube, 20 mM solution of **T** 22 µL (2 equiv.) and 20 mM solution of **NON** 43 µL (6 equiv.) were added into 585 µL $CDCl_3$ solution containing dialdehyde **pPh**, **BiPh**, **TriPh** (3 equiv. of each) and 40 µL internal standard solution. The final concentration of each dialdehydes were 1.0 mM. The reaction solution was monitored over time at 40 °C, until the equilibrium was reached. The composition of species was calculated according to the internal standard and presented on basis of imine integration data.

j) self-sorting of 3Py+3pPh+3BiPh+3TriPh+2T+9NON

In an NMR tube, 20 mM solution of **T** 44 μL (2 equiv.) and 100 mM solution of **NON** 40 μL (9 equiv.) were added into 570 μL $CDCl_3$ solution containing dialdehyde **Py**, **pPh**, **BiPh**, **TriPh** (3 equiv. of each) and 40 μL internal standard solution. The final concentration of each dialdehydes were 2.0 mM. The reaction solution was monitored over time at 40 °C, until the equilibrium was reached. The composition of species was calculated according to the internal standard and presented on basis of imine integration data.

3.2 Experimental methods for time-dependent HRMS monitoring studies

The 30 mM stock solutions of amines (**NON**, **T**) and dialdehydes (**pPh**, **BiPh**) were freshly prepared in 50%-50% $CHCl_3$/MeOH. For all the cases, HRMS kinetic experiments were carried out under dialdehyde concentration of 2 mM and were monitored as a function of time.

3.3 NOTE

The time dependent course of the reactions was sensitive to the experimental conditions. The data are only qualitative as significant variations in rates have been obtained, possibly due to sensitivity to solvent acidity despite the filtration of the solvent over an alumina column. The reaction times $t_{1/2}$ were markedly affected by the aluminium oxide used to treat the $CDCl_3$ (acidity and water) used as the solvent. To verify how could different qualities of alumina can affect the reaction courses, three different kinds of experiments were carried out: 1) experiments with fresh opened aluminum treated $CDCl_3$; 2) experiments with a dated aluminum treated $CDCl_3$. We verified that with different qualities of alumina giving these different time dependence curves, the sequence of reaction processes remained the same. For example, in the case of **pPh/NON** system, the half-consumption $t_{1/2}^C$ of **pPh** could vary from 270 min (for dated alumina treated $CDCl_3$) to 720 min (for fresh alumina treated $CDCl_3$) and the half formation time $t_{1/2}^F$ of the **pPh$_2$(NON)$_2$** macrocycle from 660 min to 2200 min in the same conditions. We verified that with different qualities of alumina giving these different time dependence curves, the sequence of reaction processes remained the same, with the sequence of three intermediates, and finally, yielded nearly 99% of **pPh$_2$(NON)$_2$** macrocycle as illustrated in Figs. S6 and S8 for more detailed information. Unless otherwise noted, the $CDCl_3$ is treated with fresh opened aluminum before using for ^{1}H NMR kinetic experiments.

4. Synthesis and Characterization

4.1 Synthesis of [1,1':4',1''-Terphenyl]-4,4''-dicarboxaldehyde (TriPh)

[1,1':4',1''-Terphenyl]-4,4''-dicarboxaldehyde was prepared via Suzuki–Miyaura Cross-coupling according to previously reported procedures.[3] The characterizations were consistent with the literature values.

^{1}H NMR (400 MHz, $CDCl_3$): δ. δ 10.08 (s, 2H), 8.00-7.98 (d, 8Hz, 4H), 7.83-7.81 (d, 8Hz, 4H), 7.77 (s, 4H)

4.2 Synthesis of macrocycle TriPh$_2$(NON)$_2$

[1,1':4':1"]Terphenyl-4,4"-dicarbaldehyde (**TriPh**) (36.41 mg; 0.13 mmol) was dissolved in 8 mL $CHCl_3$. Then, the solution was further diluted with 5 mL MeCN. Thereafter, a MeCN (3 mL) solution of 2,2'-oxybis(ethylamine) (**NON**) (12.90 mg; 0.12 mmol) was added in five portions during 20 minutes. The reaction mixture was stirred at room temperature for 7 days. After removal of the solvent by centrifugation, the crude product was washed with MeCN and dried in vacuum to afford macrocycle **TriPh$_2$(NON)$_2$** as ivory solids (38.97 mg, 88 %).

^{1}H NMR (500 MHz, $CDCl_3$, 25 °C): δ = 8.23 (s, 4H), 7.48 (t, J = 7.5 Hz, 8H), 7.38–7.35 (m, 16H), 3.80–3.70 (m, 16H)
^{13}C NMR (125 MHz, $CDCl_3$, 23 °C): δ = 162.62, 142.06, 139.38,135.18, 128.58, 127.39,126.79, 69.28, 60.47
HRMS (ESI+): m/z calcd for $C_{36}H_{36}N_4O_2$ $[M+Na]^+$ 731.3356, found 731.3339

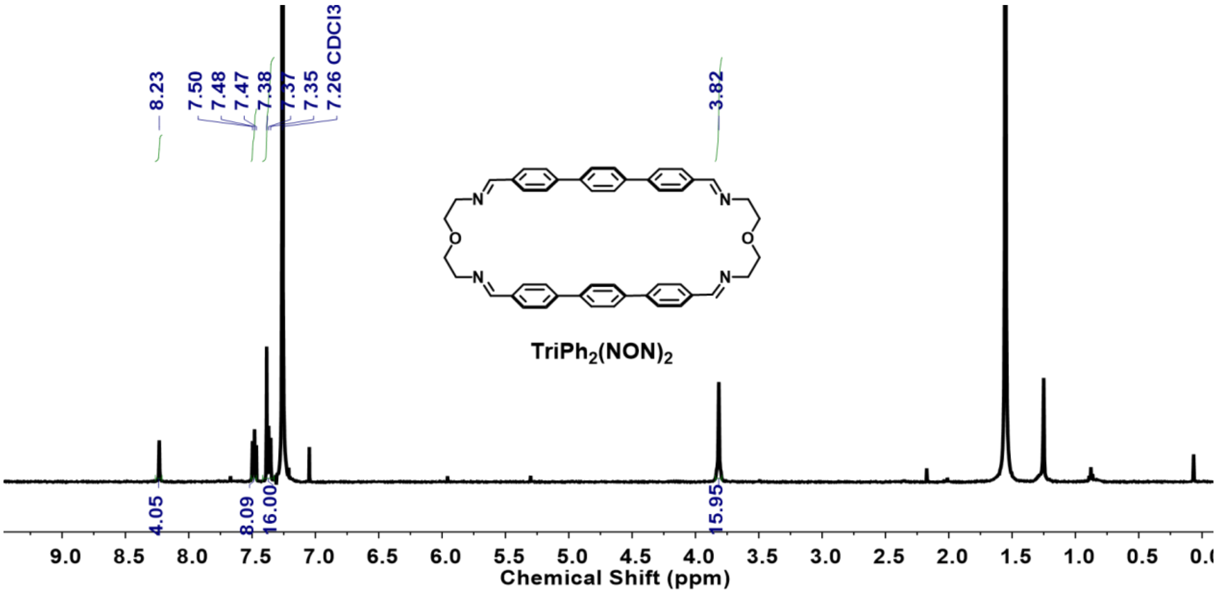

Fig. S1 ^{1}H NMR spectra (500 MHz, $CDCl_3$, 25 °C) of Macrocycle **TriPh$_2$(NON)$_2$**.

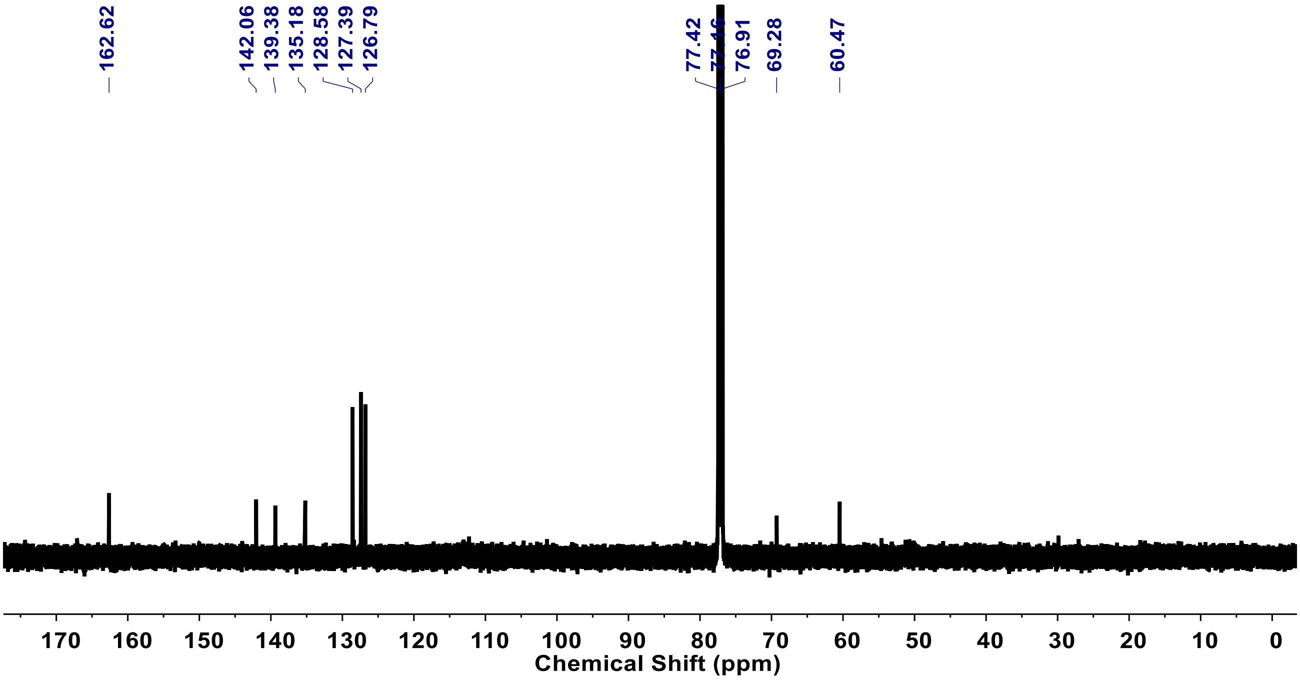

Fig. S2 ^{13}C NMR spectra (125 MHz, $CDCl_3$, 25 °C) of Macrocycle **TriPh$_2$(NON)$_2$**.

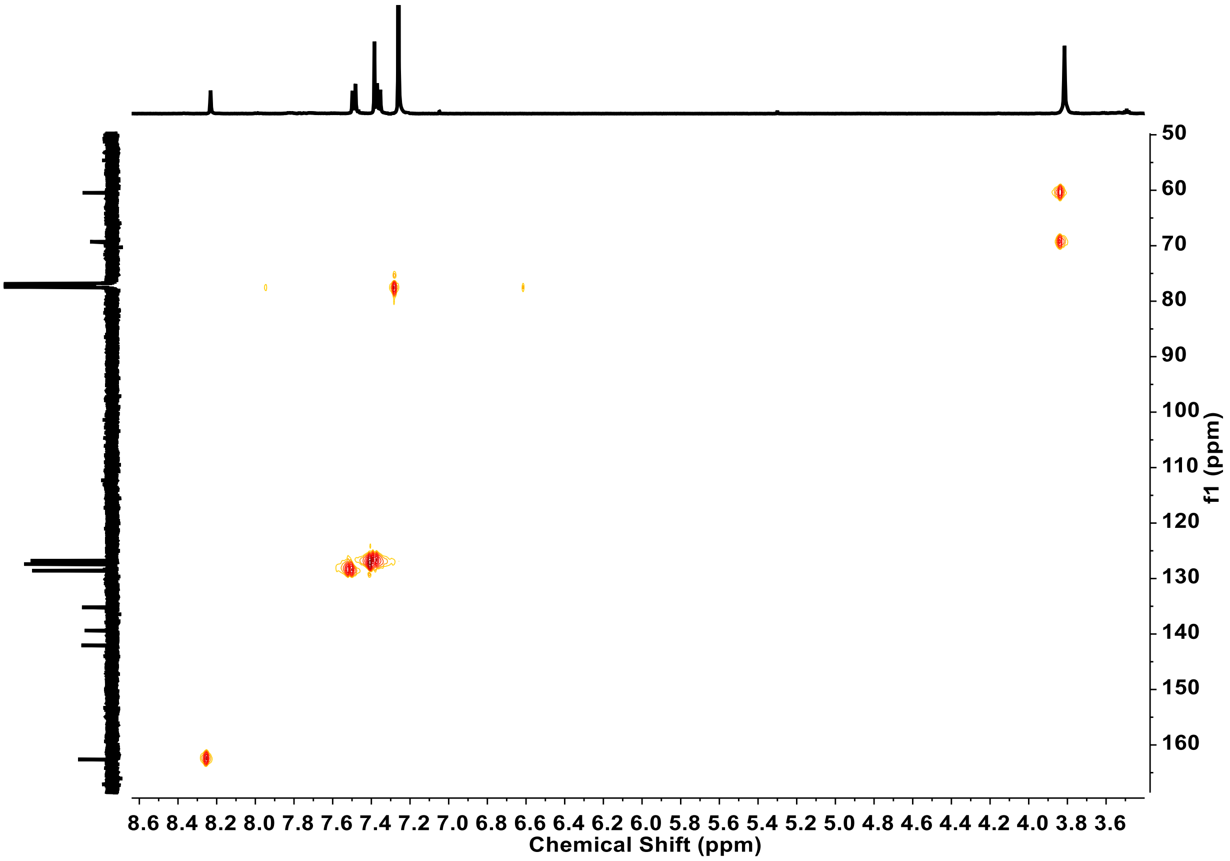

Fig. S3 HSQC spectra (500 MHz of ^1H, 125 MHz of ^{13}C, $CDCl_3$, 23 °C) of Macrocycle **TriPh$_2$(NON)$_2$**.

4.3. Synthesis of Macrobicyclic Cage TriPh$_3$T$_2$

[1,1':4':1"]Terphenyl-4,4"-dicarbaldehyde (**TriPh**) (33.15 mg; 0.12 mmol) was dissolved in 8 mL $CHCl_3$. Then, the solution was further diluted with 3 mL MeOH. Thereafter, a MeOH (2 mL) solution of Tris(2-aminoethyl)amine (**T**) (11.07 mg; 0.076 mmol) was added in five portions during 20 minutes. The reaction mixture was stirred at room temperature for 3 days. After removal of the solvent by centrifugation, the crude product was washed with 1 mL $CHCl_3$ and MeOH, then dried in vacuum to afford macrobicyclic cage **TriPh$_3$T$_2$** as yellow solids (30.21 mg, 76 %).

^1H NMR (400 MHz, $CDCl_3$, 25 °C): δ = 8.30 (s, 6H), 7.31-7.22 (m, $CDCl_3$ included), 7.19-7.16 (m, 24H), 3.85 (m, 12H), 2.84 (m, 12H)
HRMS (ESI+): m/z calcd for $[M+H]^+$ 1043.5483, found 1043.5463.

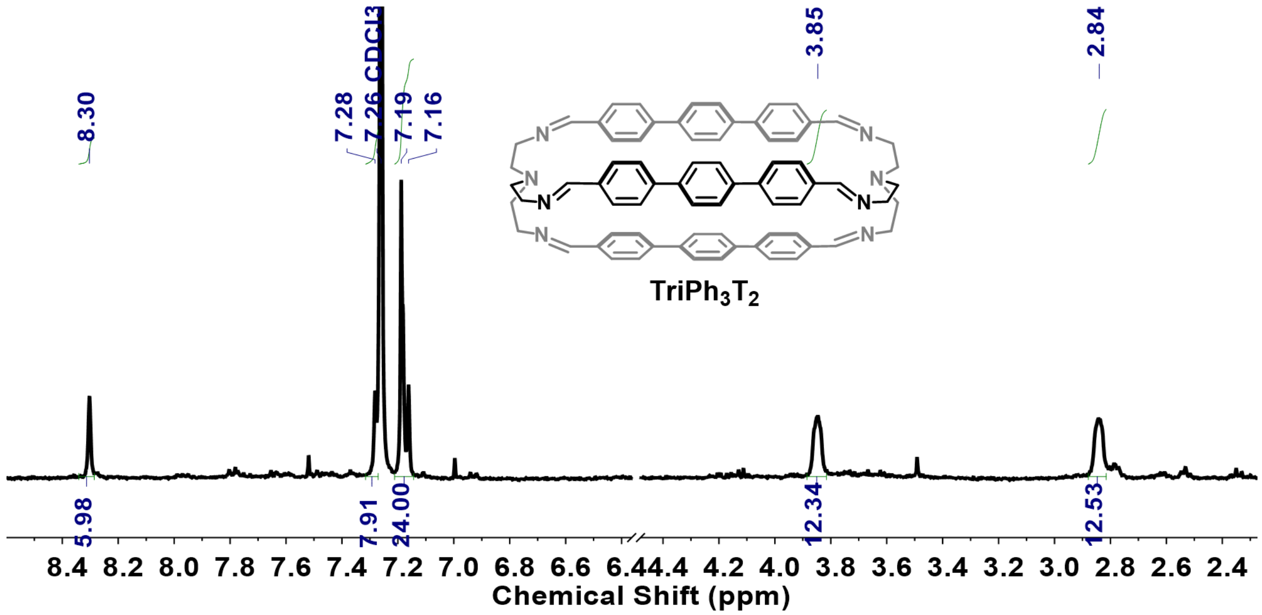

Fig. S4 ^{1}H NMR spectra (500 MHz, $CDCl_3$, 25 °C) of macrobicyclic cage **$TriPh_3T_2$**

The synthesis procedure of the other macrocycles and macrobicyclic cages are reported in our previous papers.[4,5]

5. Formation of isolated macrocycles

5.1 Formation of macrocycle $pPh_2(NON)_2$

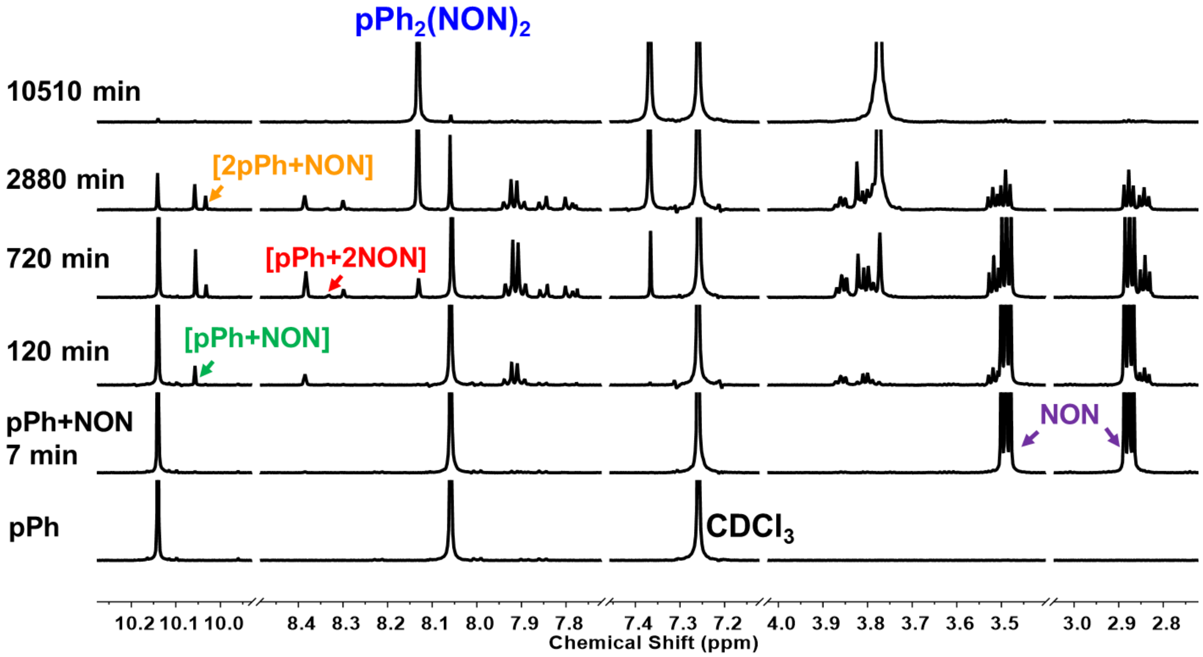

Fig. S5 Temporal evolution of the ^{1}H NMR spectra (500 MHz, $CDCl_3$, 23 °C) showing the formation of intermediates and of the final macrocycle **pPh₂(NON)₂** in fresh aluminum treated $CDCl_3$.

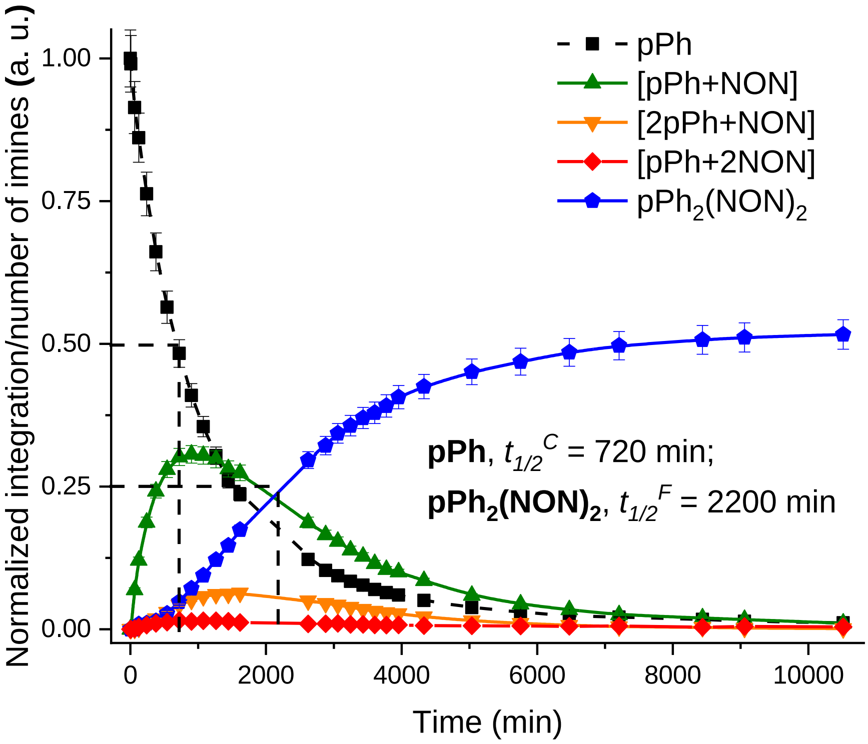

Fig. S6 ^{1}H NMR monitoring during the formation of macrocycle **$pPh_2(NON)_2$** in freshly opened aluminum treated $CDCl_3$.

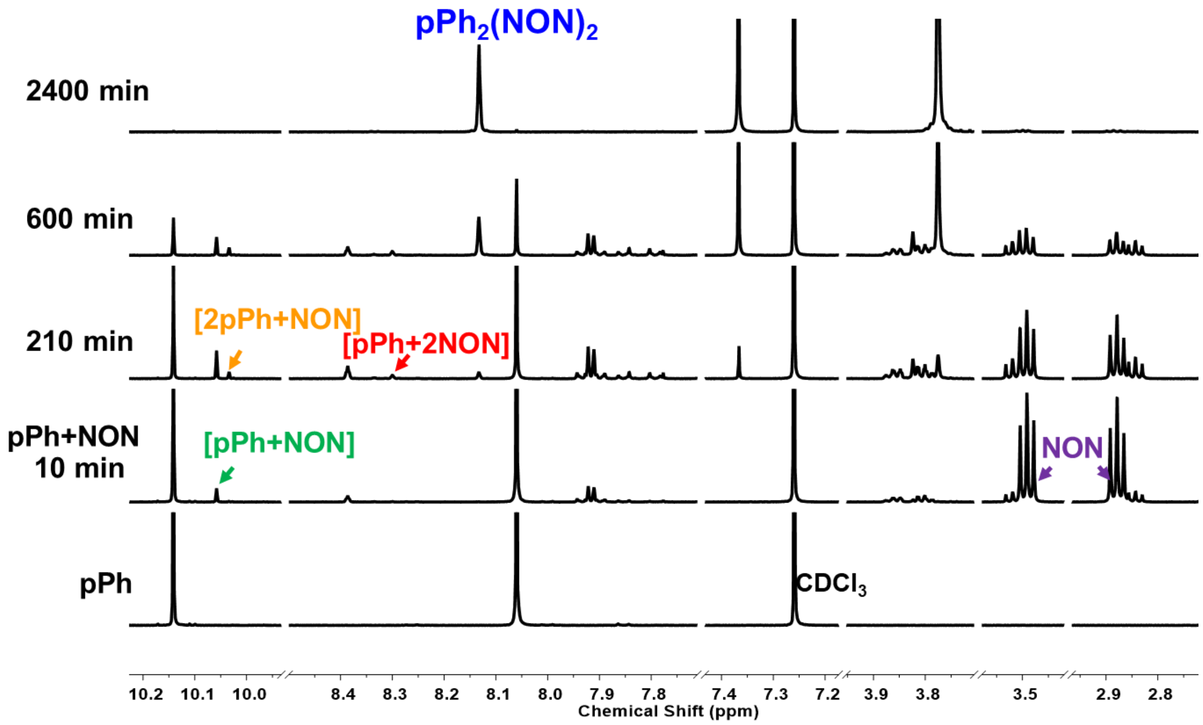

Fig. S7 Temporal evolution of the ^{1}H NMR spectra (500 MHz, $CDCl_3$, 23 °C) showing the formation of intermediates and of the final macrocycle **pPh$_2$(NON)$_2$** in dated aluminum treated $CDCl_3$.

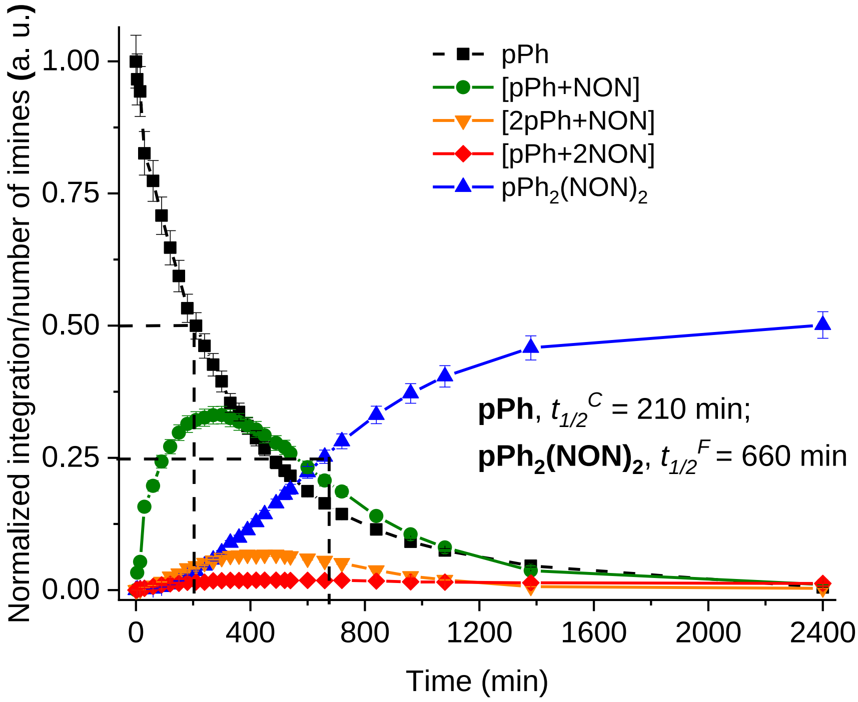

Fig. S8 ^{1}H NMR monitoring during the formation of macrocycle **pPh$_2$(NON)$_2$** in dated aluminum treated $CDCl_3$.

5.2 Formation of macrocycle $BiPh_2(NON)_2$

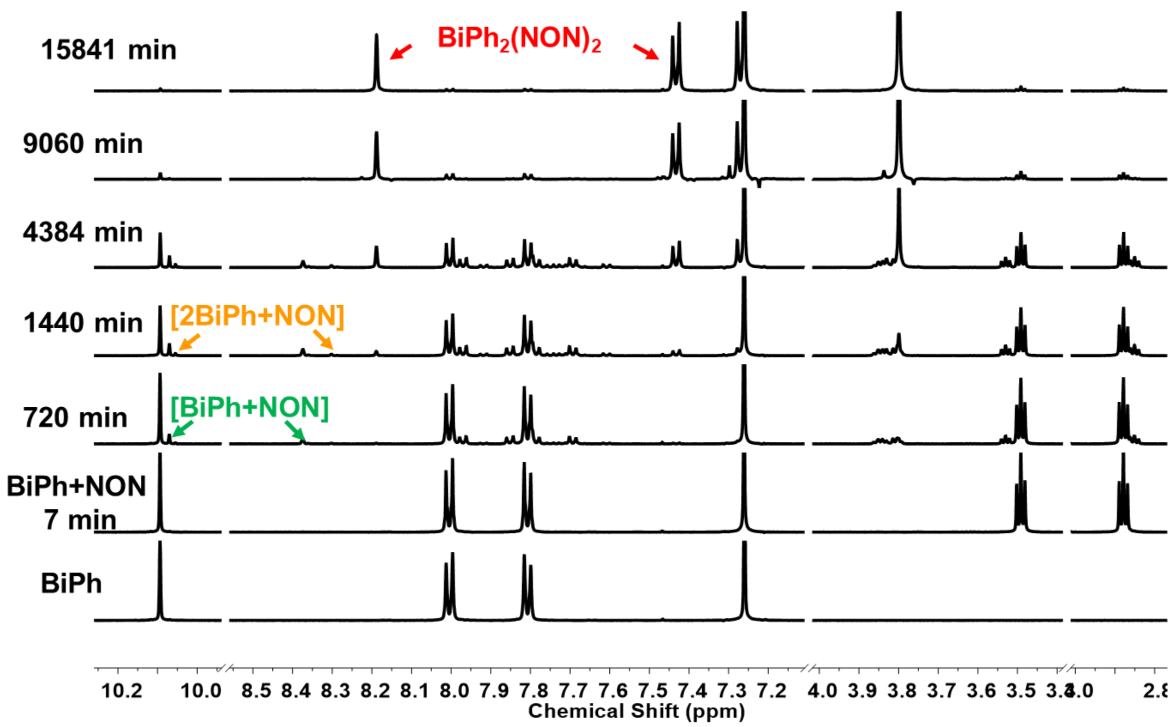

Fig. S9 Temporal evolution of the ^{1}H NMR spectra (500 MHz, $CDCl_3$) of a 1/1 mixture of **BiPh** and **NON** (3.6 mM) showing the formation of two intermediates and of the final macrocycle **$BiPh_2(NON)_2$**.

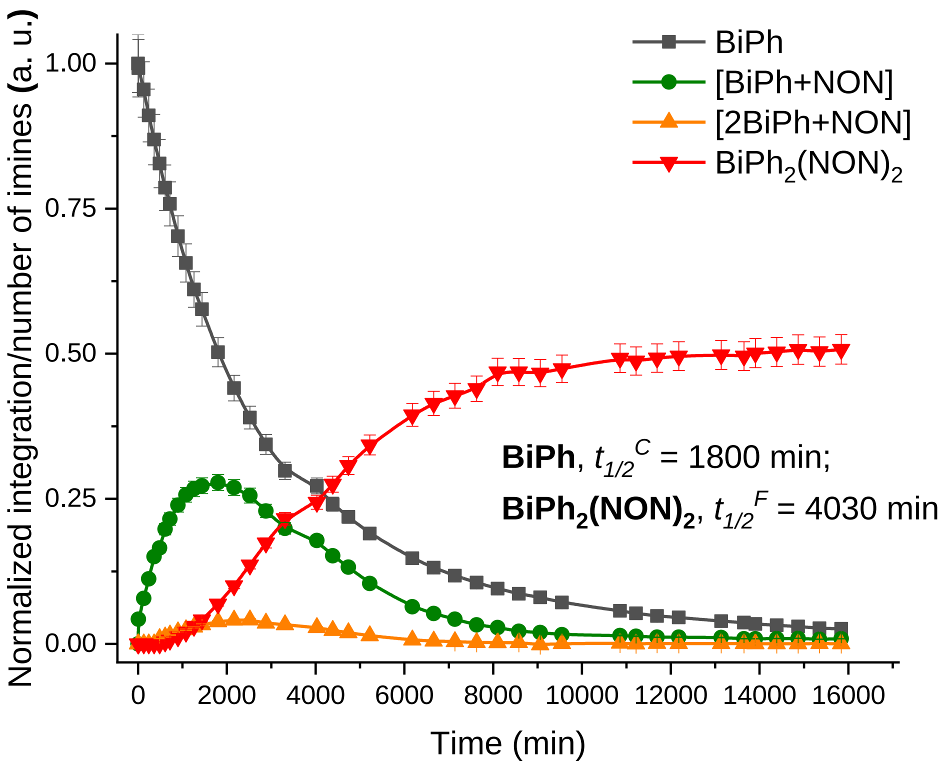

Fig. S10 ^{1}H NMR monitoring of the evolution of a 1/1 mixture of components **BiPh** and **NON** (3.6 mM each in $CDCl_3$) as a function of time over 15841 min. Error in ^{1}H-NMR signal integration: ±5%.

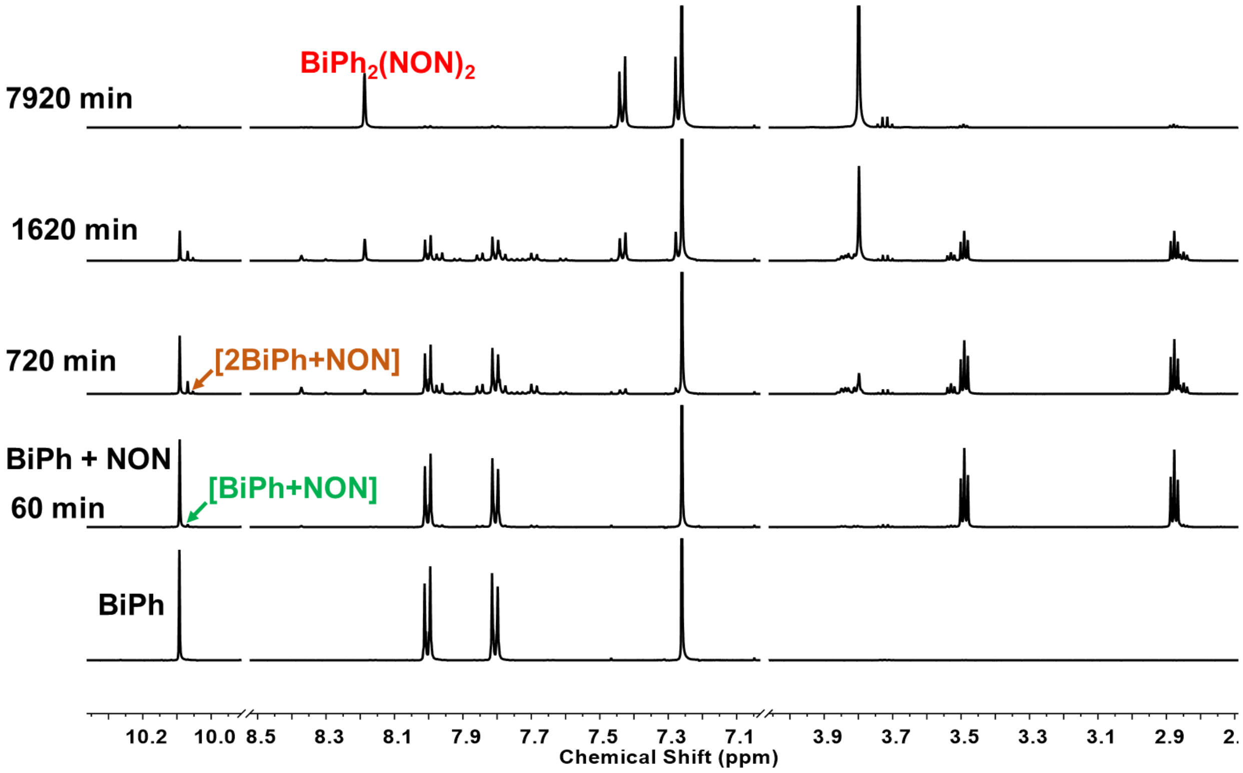

Fig. S11 Temporal evolution of the ^{1}H NMR spectra (500 MHz, 23 °C, dated aluminum treated $CDCl_3$) showing the formation of intermediates and of the final macrocycle **BiPh$_2$(NON)$_2$** in dated aluminum treated $CDCl_3$.

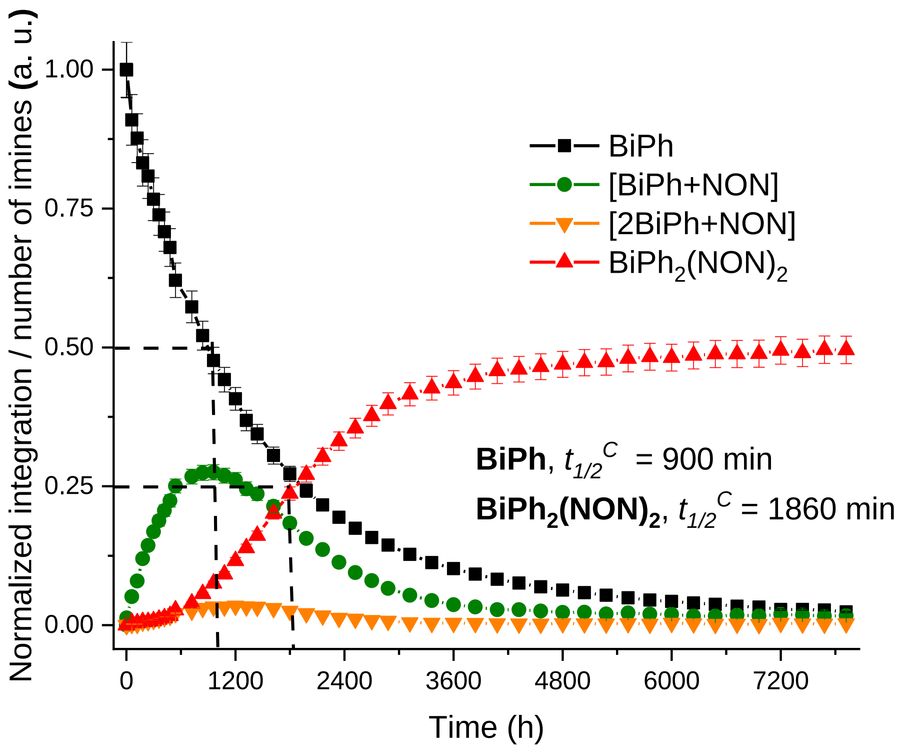

Fig. S12 ^{1}H NMR monitoring of the evolution of the species generated during macrocycle **BiPh$_2$(NON)$_2$** formation in dated aluminum treated $CDCl_3$ as a function of time over 7920 min. Error in ^{1}H-NMR signal integration: ±5%.

5.3 Formation of macrocycle $Py_2(NON)_2$

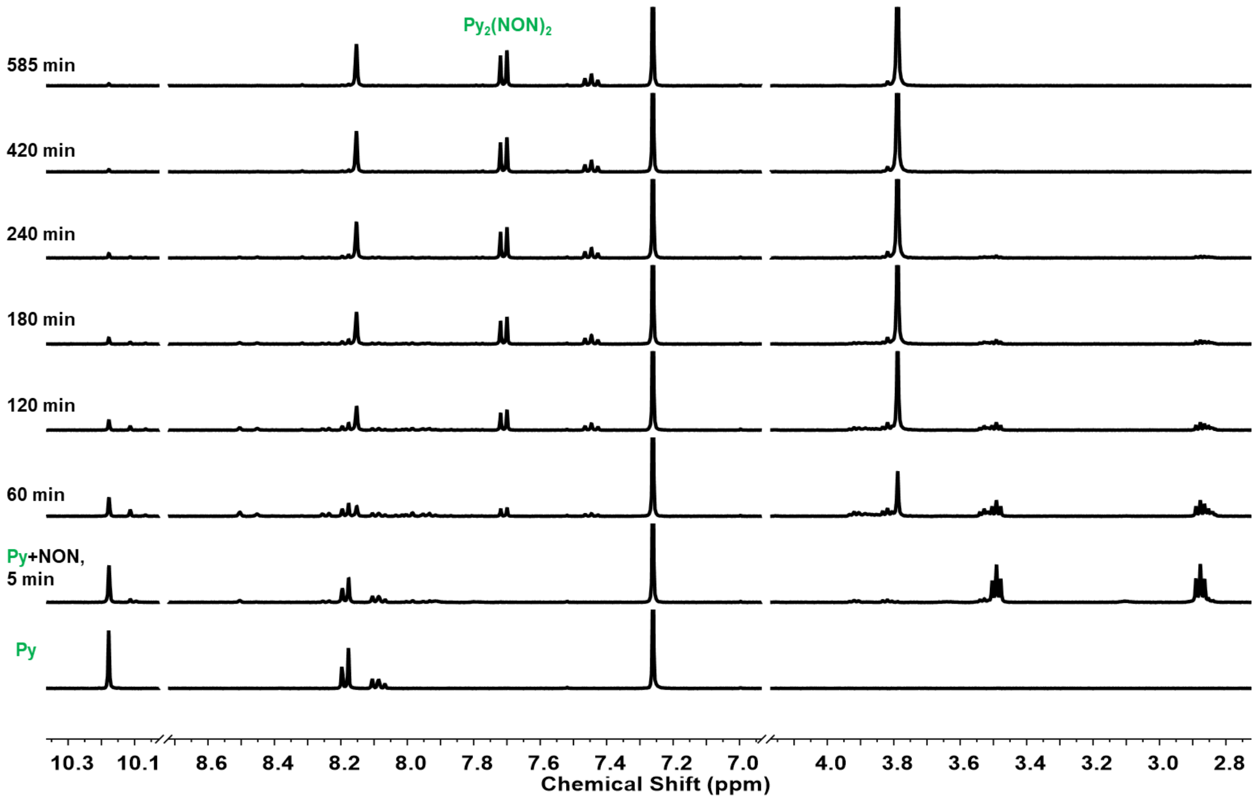

Fig. S13 Temporal evolution of the ^{1}H NMR spectra (400 MHz, $CDCl_3$, 21 °C) of a 1/1 mixture of **Py** and **NON** (3.6 mM) showing the formation of two intermediates and of the final macrocycle **$Py_2(NON)_2$**.

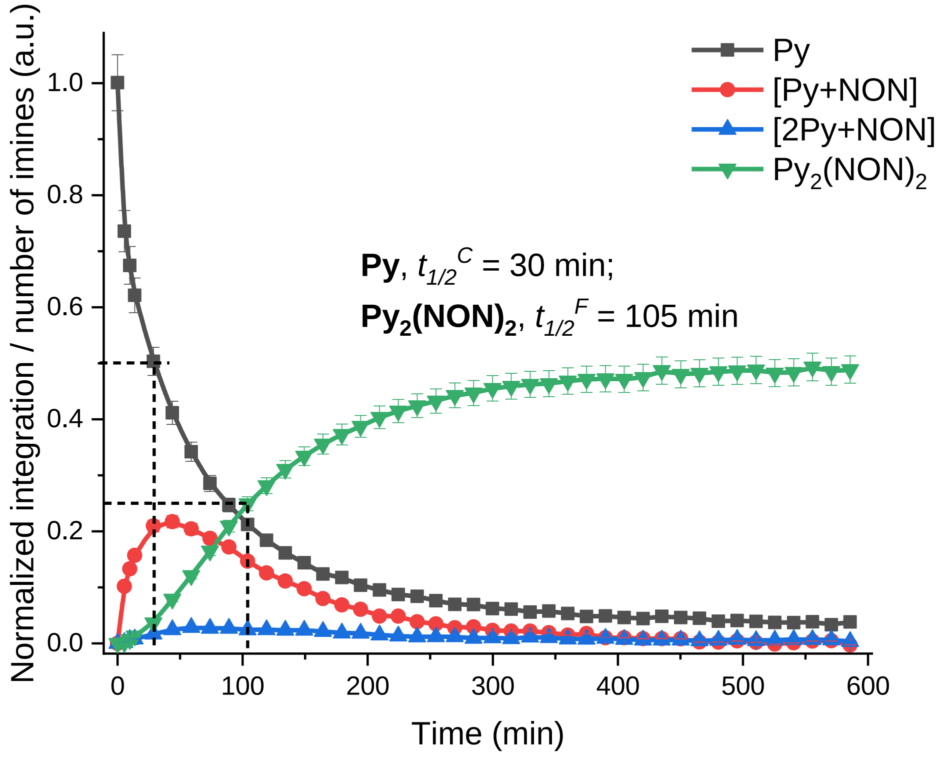

Fig. S14 ^{1}H NMR monitoring of the evolution of a 1/1 mixture of components **Py** and **NON** (3.6 mM each in $CDCl_3$) as a function of time over 586 min. Error in ^{1}H-NMR signal integration: ±5%.

5.4 Time-dependent HRMS of macrocycle pPh$_2$(NON)$_2$

Table S1. HRMS-ESI assignments of the Key Species Identified during the formation process of macrocycle **pPh$_2$(NON)$_2$**

Entry	assignment	formula	Combined ion	m/z calcd.	m/z found
1	**NON**	$C_4H_{12}N_2O$	$+H^+$	105.1022	105.1024
			$+Na^+$	127.0842	127.0840
2	[**pPh**+**NON**]-H_2O	$C_{12}H_{16}N_2O_2$	$+H^+$	221.1285	221.1276
			$+Na^+$	243.1104	243.1095
			$+2H^+$	111.0679	N/A
3	[**pPh**+2**NON**]-2H_2O	$C_{16}H_{26}N_4O_2$	$+H^+$	307.2129	307.2115
			$+Na^+$	329.1948	329.1934
			$+2H^+$	154.1101	154.1095
4	[2**pPh**+**NON**]-2H_2O	$C_{20}H_{20}N_2O_3$	$+H^+$	337.1547	337.1531
			$+Na^+$	359.1372	359.1351
			$+2H^+$	169.0810	N/A
5	[2**pPh**+2**NON**]-3H_2O	$C_{24}H_{30}N_4O_3$	$+H^+$	423.2391	N/A
			$+Na^+$	445.2210	445.2193
			$+2H^+$	212.1232	212.1386
6	[2**pPh**+2**NON**]-4H_2O	$C_{24}H_{28}N_4O_2$	$+H^+$	405.2285	405.2269
			$+Na^+$	427.2104	427.2089
			$+2H^+$	203.1179	N/A

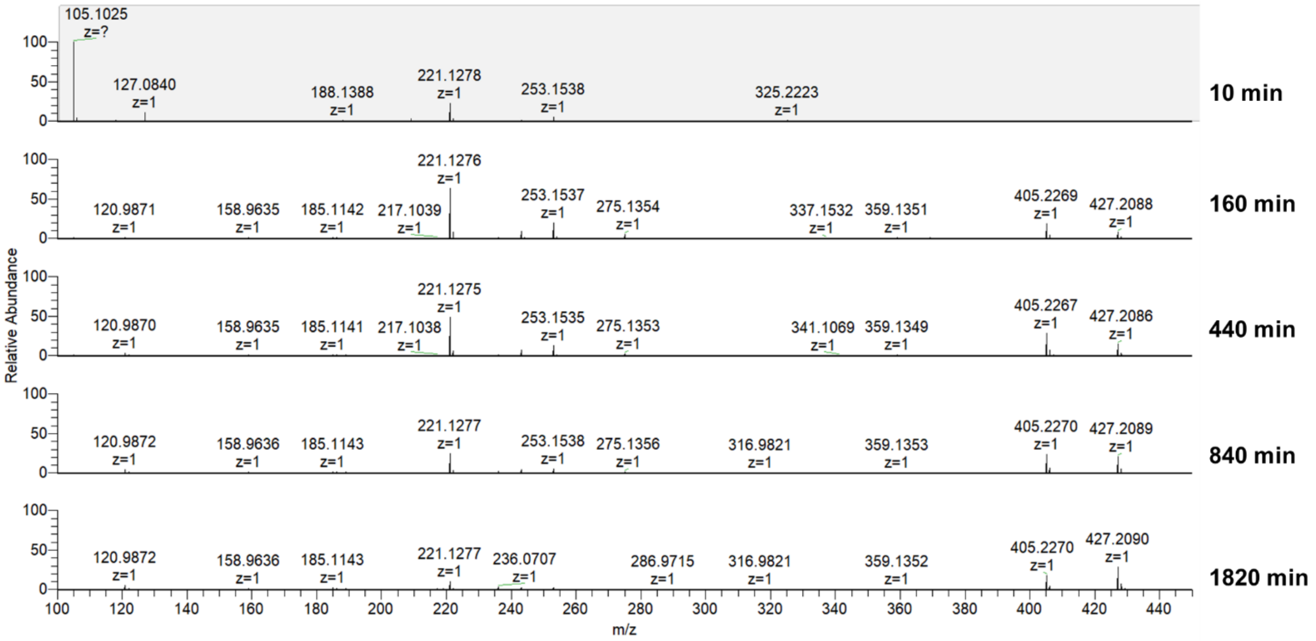

Fig. S15 Evolution of the HRMS-ESI spectra of a 1/1 mixture of **pPh** and **NON** (2 mM each in 50%-50% $CHCl_3$/MeOH).

5.5 Time-dependent HRMS of macrocycle **BiPh$_2$(NON)$_2$**

Table S2. HRMS-ESI assignment of the Key Species Identified during the formation process of macrocycle **BiPh$_2$(NON)$_2$**

Entry	assignment	formula	Combined ion	m/z calcd.	m/z found
1	**NON**	$C_4H_{12}N_2O$	$+H^+$	105.1022	105.1028
			$+Na^+$	127.0842	127.0844
2	[**BiPh**+**NON**]-H_2O	$C_{18}H_{20}N_2O_2$	$+H^+$	297.1598	297.1597
			$+Na^+$	319.1417	319.1416
			$+2H^+$	149.0835	N/A
3	[**BiPh**+2**NON**]-2H_2O	$C_{22}H_{30}N_4O2$	$+H^+$	383.2442	383.2412
			$+Na^+$	405.2261	405.2258
			$+2H^+$	192.1257	192.1256
4	[2**BiPh**+**NON**]-2H_2O	$C_{32}H_{28}N_2O3$	$+H^+$	489.2173	489.2140
			$+Na^+$	511.1992	511.1960
			$+2H^+$	245.1123	N/A
5	[2**BiPh**+2**NON**]-3H_2O	$C_{36}H_{38}N_4O_3$	$+H^+$	575.3017	575.2980
			$+Na^+$	597.2836	597.2926
			$+2H^+$	288.1545	N/A
6	[2**BiPh**+2**NON**]-4H_2O	$C_{36}H_{36}N_4O_2$	$+H^+$	557.2911	557.2876
			$+Na^+$	579.2730	579.2696
			$+2H^+$	279.1492	279.1473

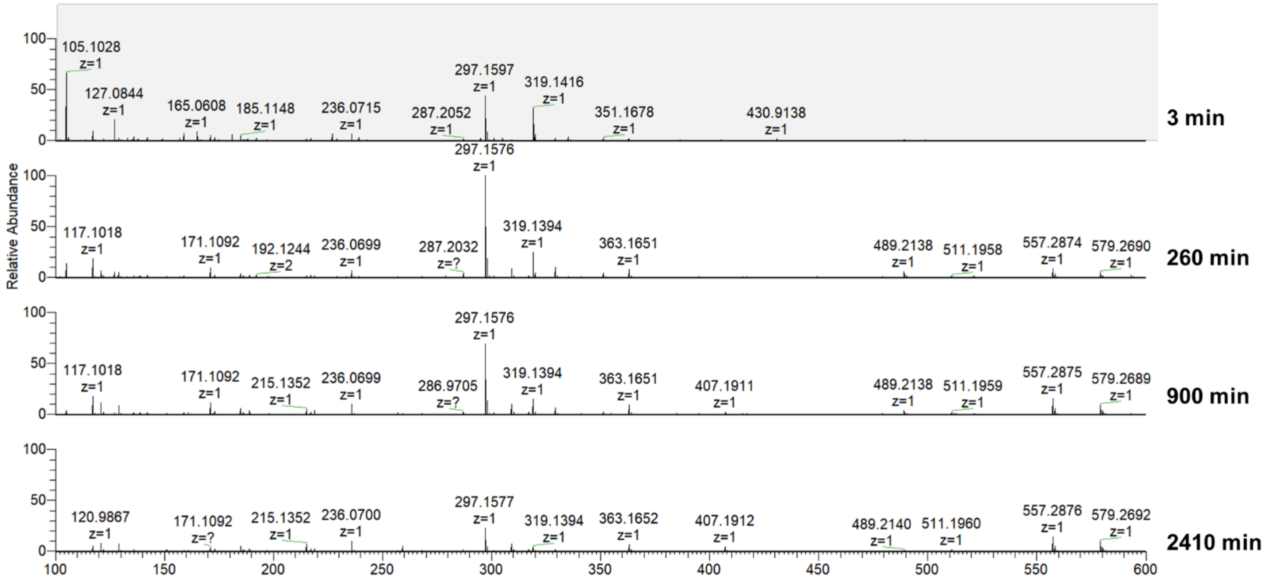

Fig. S16 Evolution of the HRMS-ESI spectra of a 1/1 mixture of **BiPh** and **NON** (2 mM each in 50%-50% $CHCl_3$/MeOH).

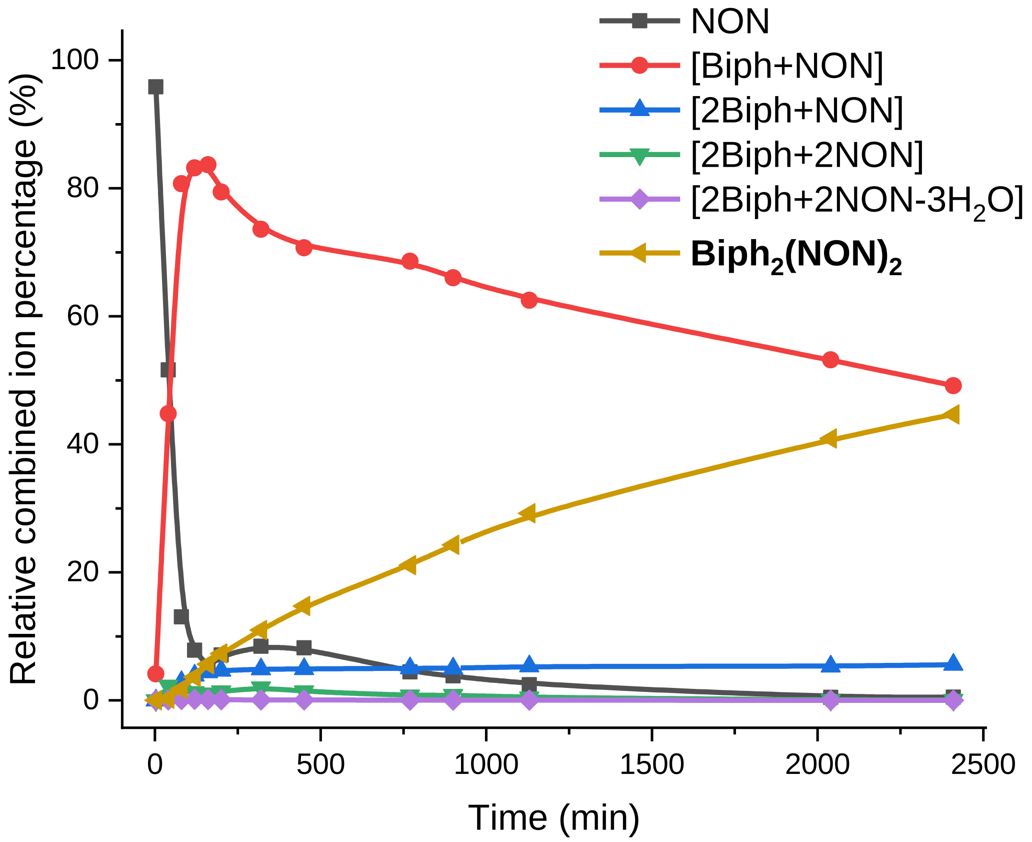

Fig. S17 HRMS-ESI monitoring of the evolution of the species generated during the formation of the macrocycle **BiPh$_2$(NON)$_2$** from **BiPh** and **NON** (2 mM each, 50%-50% $CHCl_3$/MeOH, r.t) as a function of time over 2410 min. NB: The relative combined ion percentage is obtained by the ratio of combined ion count of each species to the sum of combined ion counts for all species at each time point. These data do not provide quantitative information about the relative amounts of each species identified by its mass, but, taken separately, they display the evolution of a given identified species during the course of the reaction. The curves are added to guide the eye.

6. Formation of isolated macrobicyclic cages

6.1 Time-dependent ^{1}H NMR of macrobicyclic cage pPh₃T₂

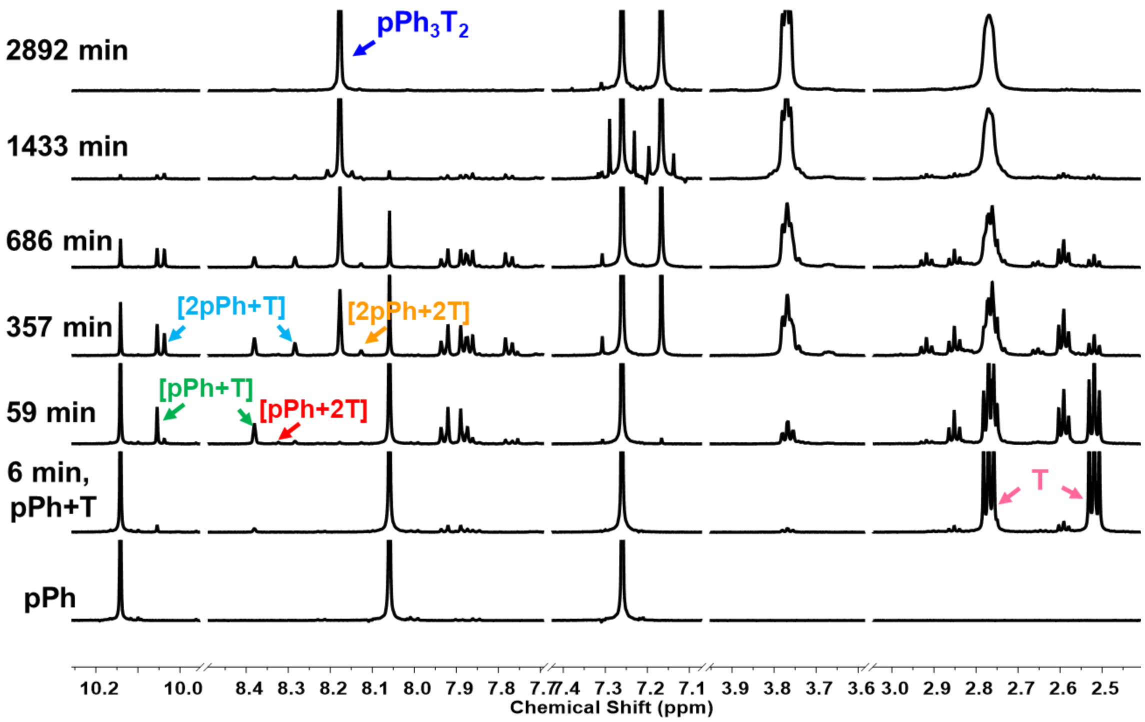

Fig. S18 Temporal evolution of the ^{1}H NMR spectra (500 MHz, $CDCl_3$, 23 °C) of a 3/2 mixture of **pPh** (3.6 mM) and **T** showing the formation of intermediates and of the final macrobicyclic cage **pPh_3T_2.**

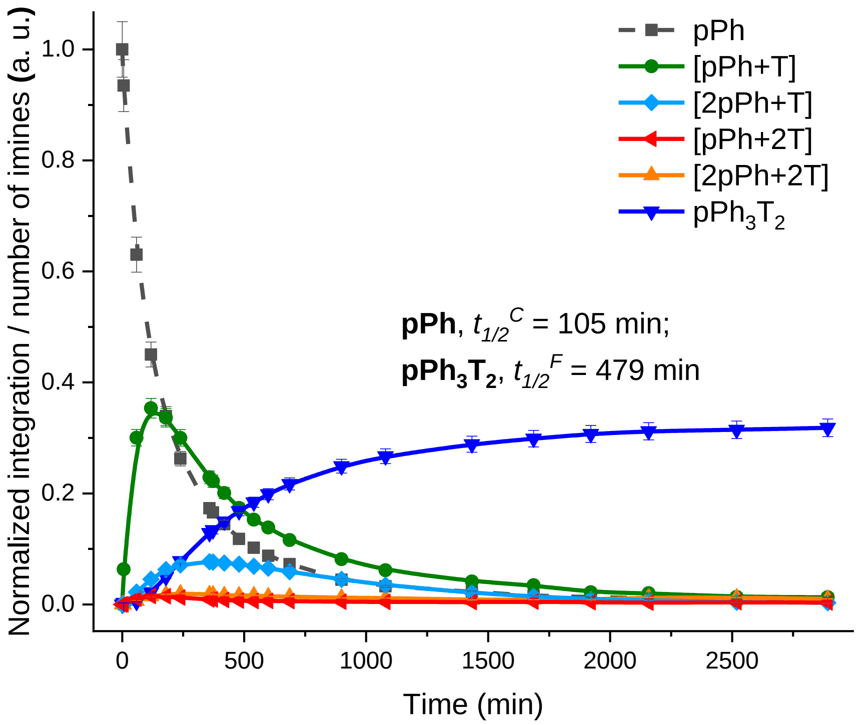

Fig. S19 ^{1}H NMR monitoring of the evolution of the species generated during cage **pPh_3T_2** formation as a function of time over 2892 min. Error in ^{1}H-NMR signal integration: ±5%.

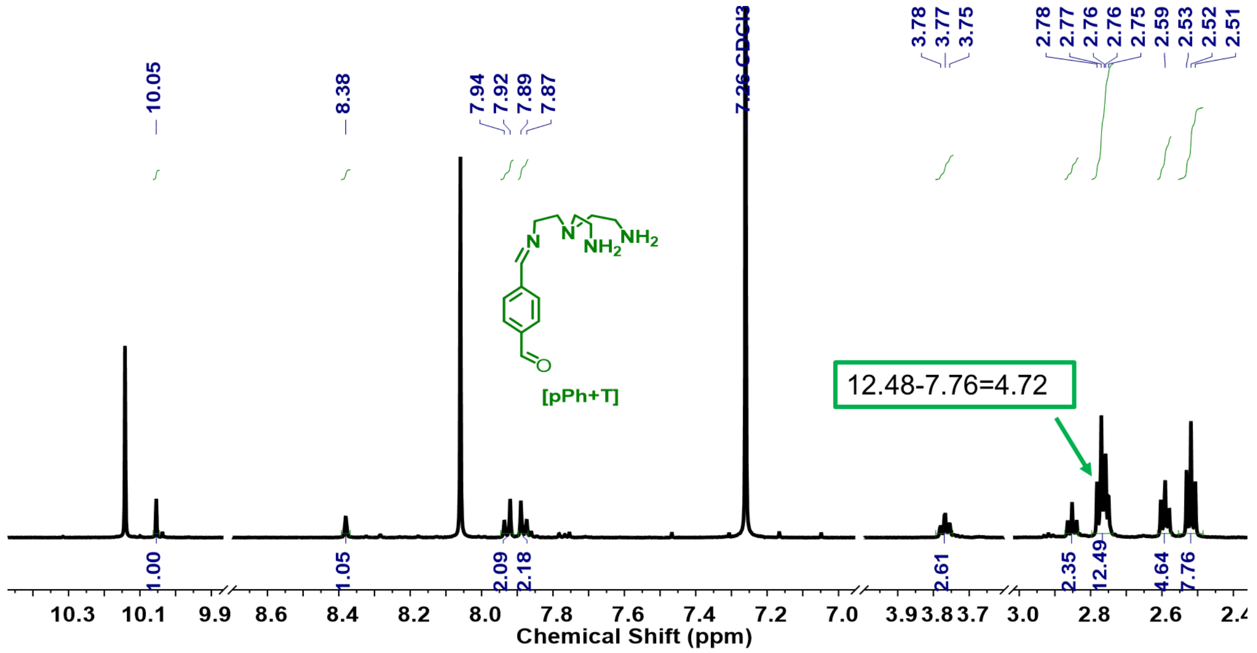

Fig. S20 ^{1}H NMR spectra (400 MHz, $CDCl_3$) of the intermediate [**pPh**+**T**] after 59 min condensation of **pPh** (3.6 mM) and **T** (2.4 mM).

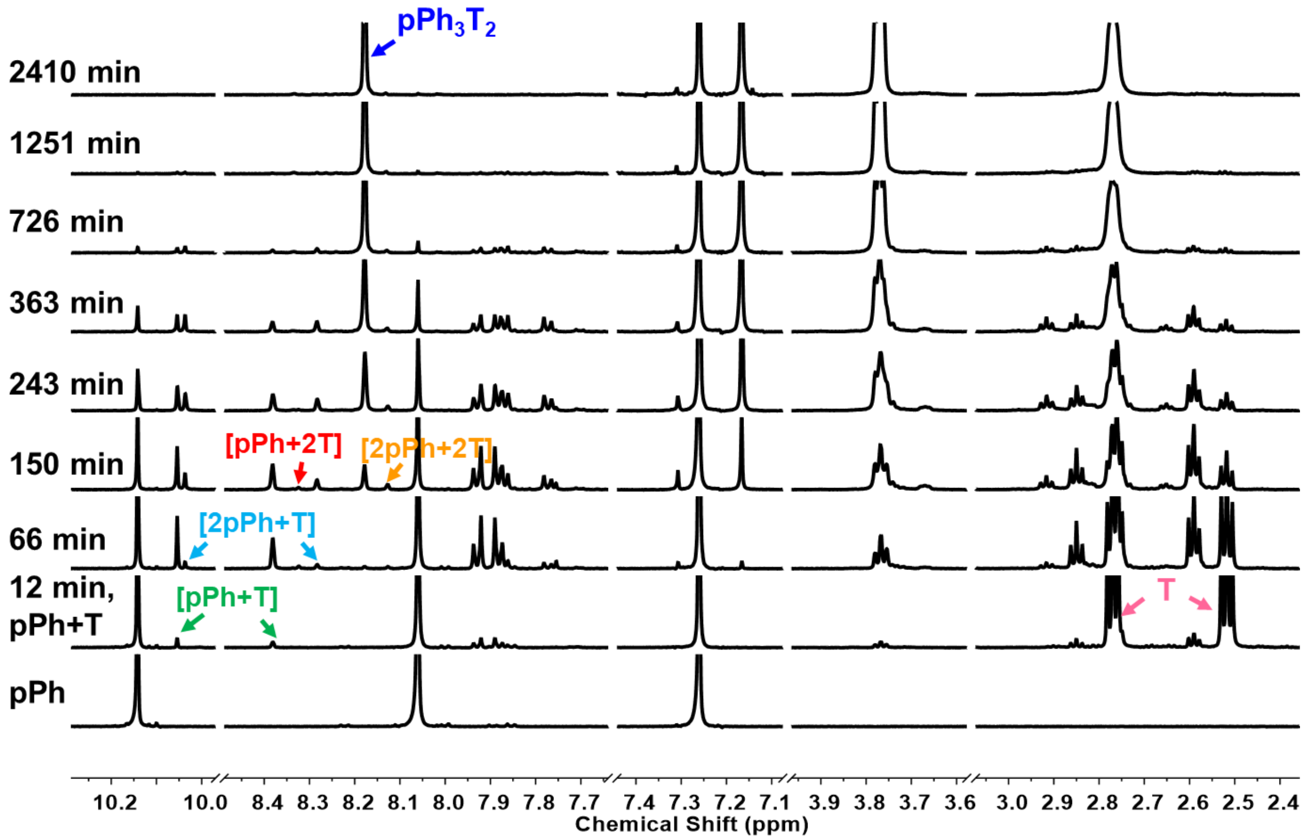

Fig. S21 Temporal evolution of the ^{1}H NMR spectra (500 MHz, CD_3Cl, 23 °C) of a 3/2 mixture of **pPh** (3.6 mM) and **T** (2.4 mM) showing the formation of several intermediates and of the final macrobicyclic cage **pPh_3T_2** in dated aluminum treated $CDCl_3$.

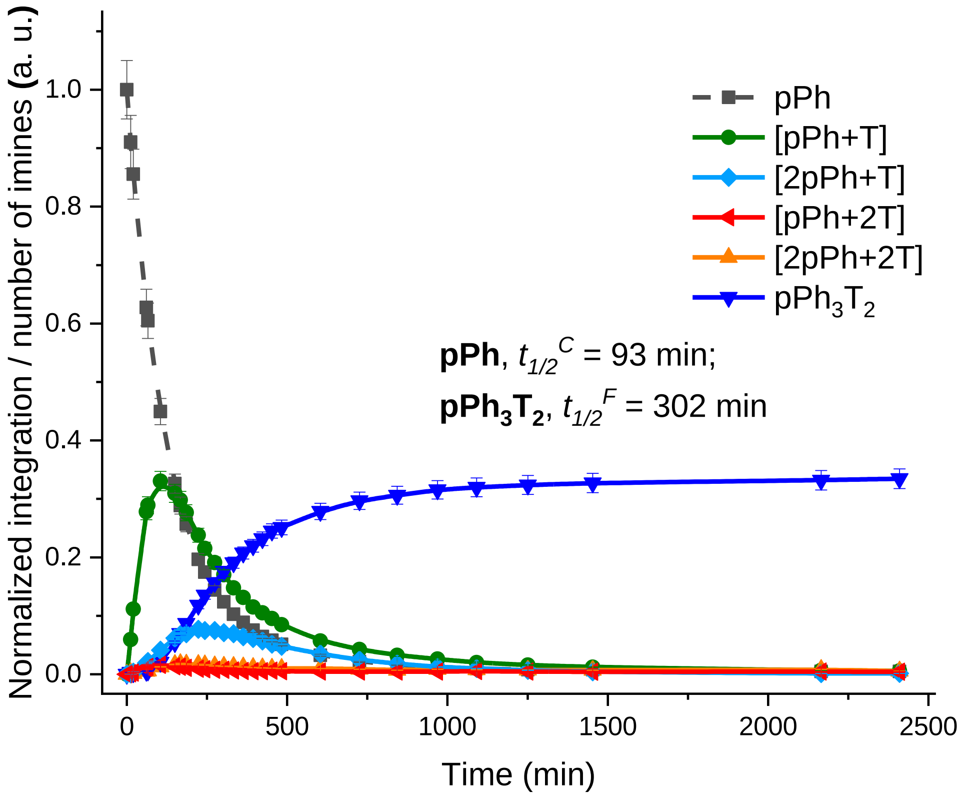

Fig. S22 ^{1}H NMR monitoring of the evolution of the species generated during cage $\mathbf{pPh_3T_2}$ formation as a function of time over 2410 min in dated aluminum treated $CDCl_3$. Error in ^{1}H-NMR signal integration: ±5%.

Assignment of [pPh+2T] and [2pPh+2T] according to reaction 3pPh + 4T

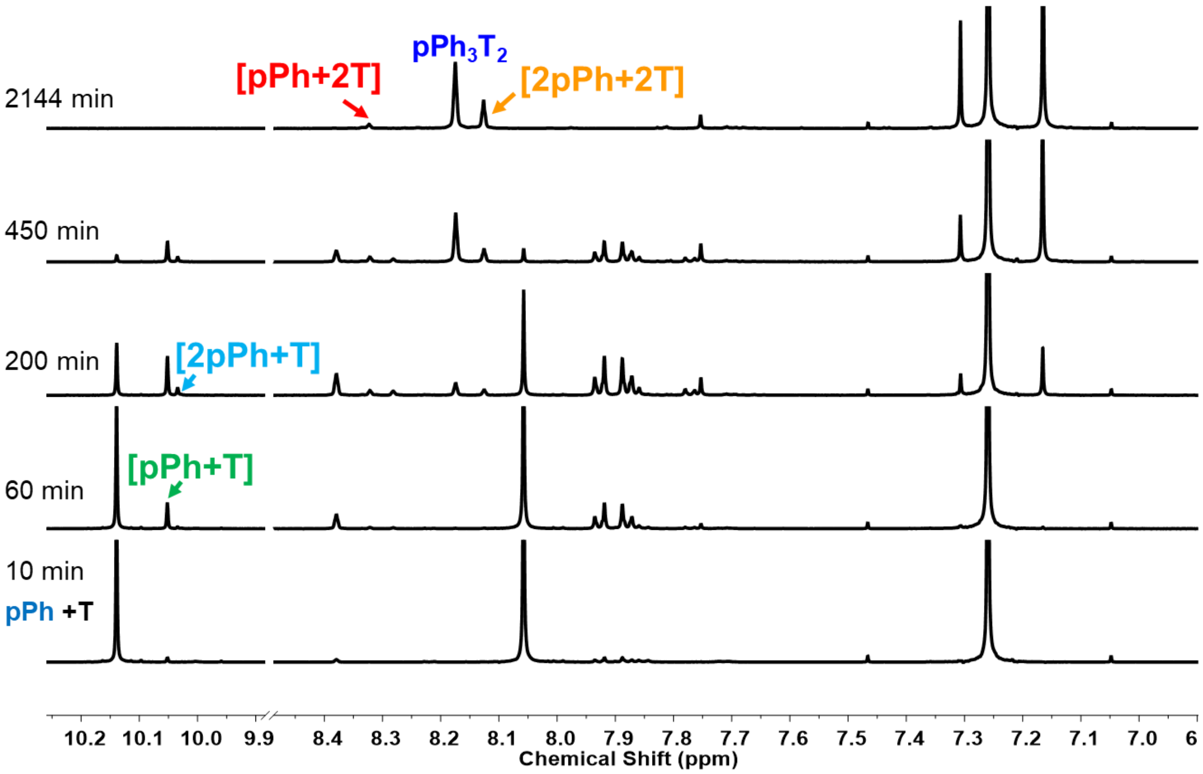

Fig. S23 Temporal evolution of the ^{1}H NMR (500 MHz, $CDCl_3$, 25 °C) spectra of 3**pPh** + 4**T** reaction showing

the formation of several intermediates and of the final macrobicyclic cage **pPh$_3$T$_2$**

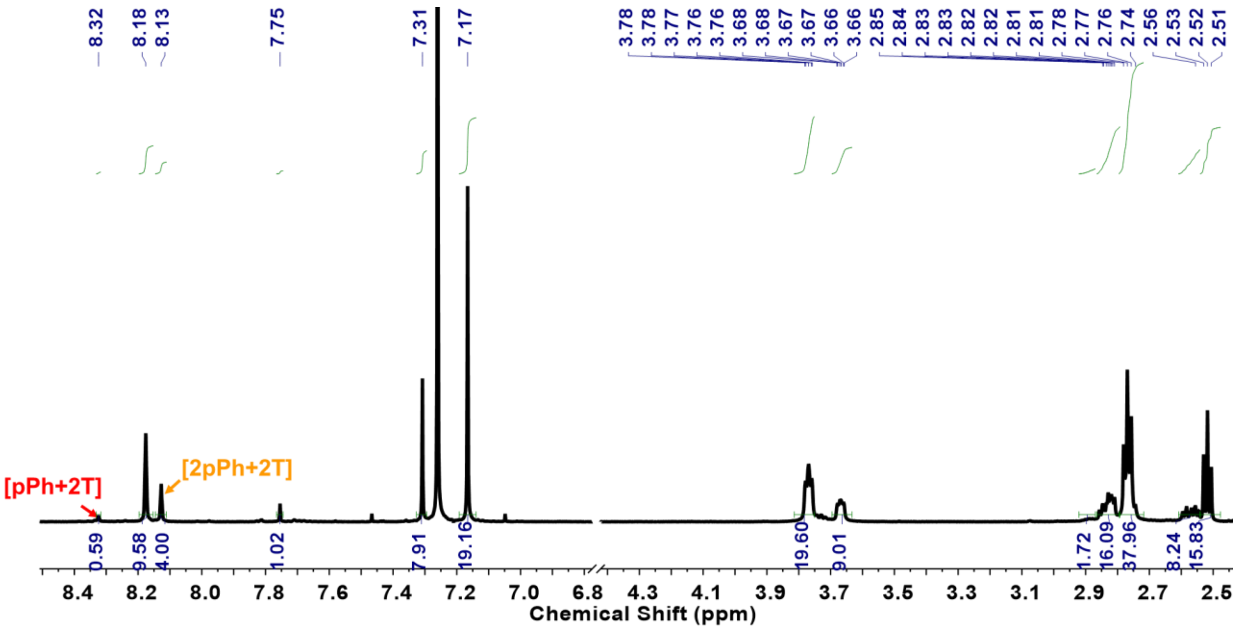

Fig. S24 Equilibrated ^{1}H NMR (500 MHz, $CDCl_3$, 25 °C) spectrum of 3**pPh** + 4**T** reaction after 2144 min.

6.2 Time-dependent ^{1}H NMR of macrobicyclic cage BiPh$_3$T$_2$

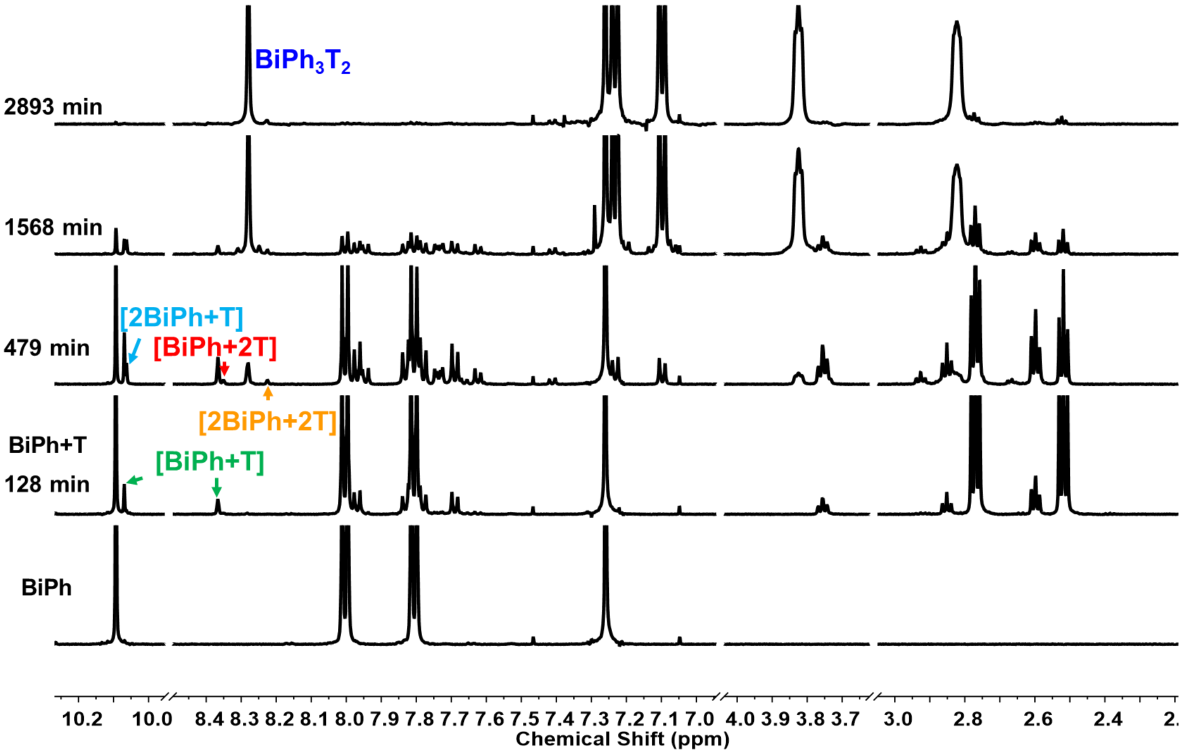

Fig. S25 Temporal evolution of the ^{1}H NMR spectra (500 MHz, $CDCl_3$) of a 3/2 mixture of **BiPh** (3.6 mM) and **T** showing the formation of several intermediates and of the final macrobicyclic cage **BiPh$_3$T$_2$**.

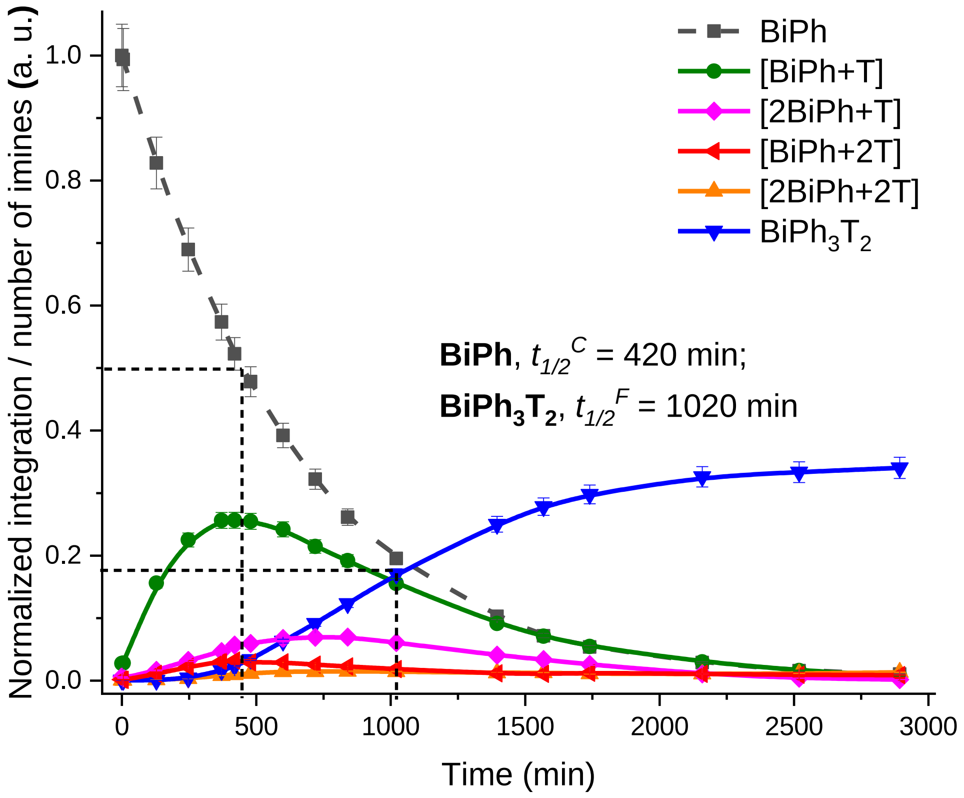

Fig. S26 ^{1}H NMR monitoring of the evolution of cage **BiPh$_3$T$_2$** formation as a function of time over 2893 min. Error in ^{1}H-NMR signal integration: ±5%.

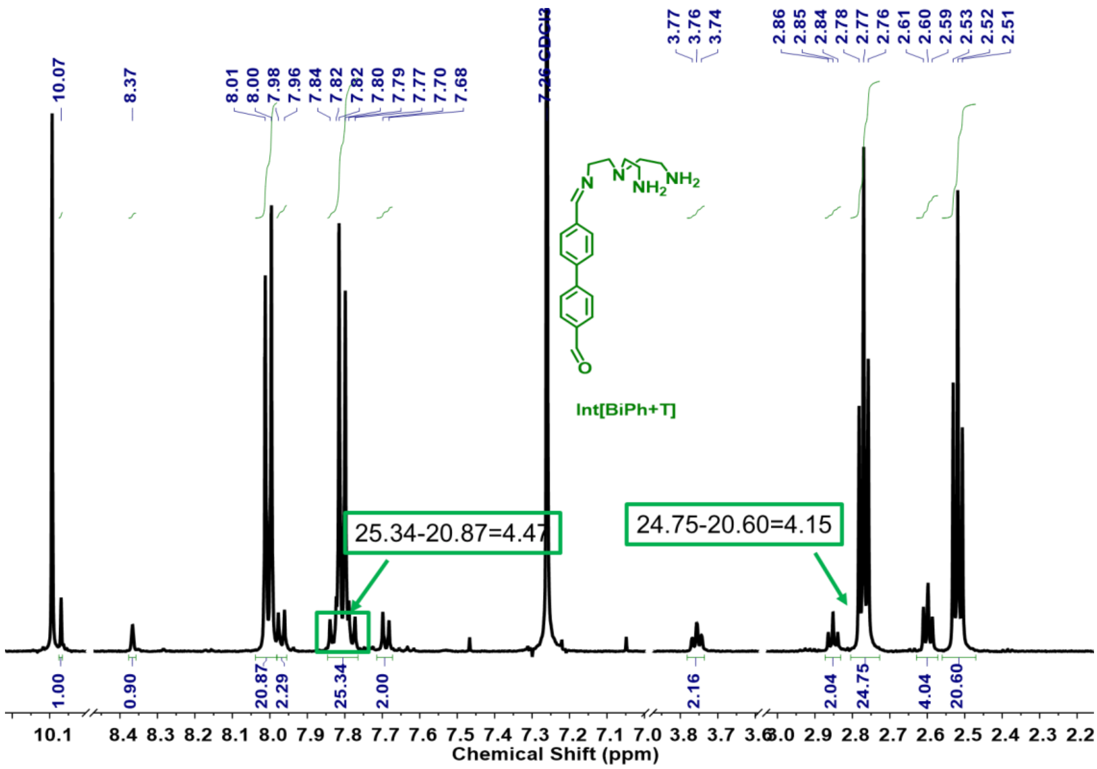

Fig. S27 ^{1}H NMR spectra (500 MHz, $CDCl_3$) of the intermediate [**BiPh**+**T**] after 168 min condensation of **BiPh** (3.6 mM) and **T** (2.4 mM).

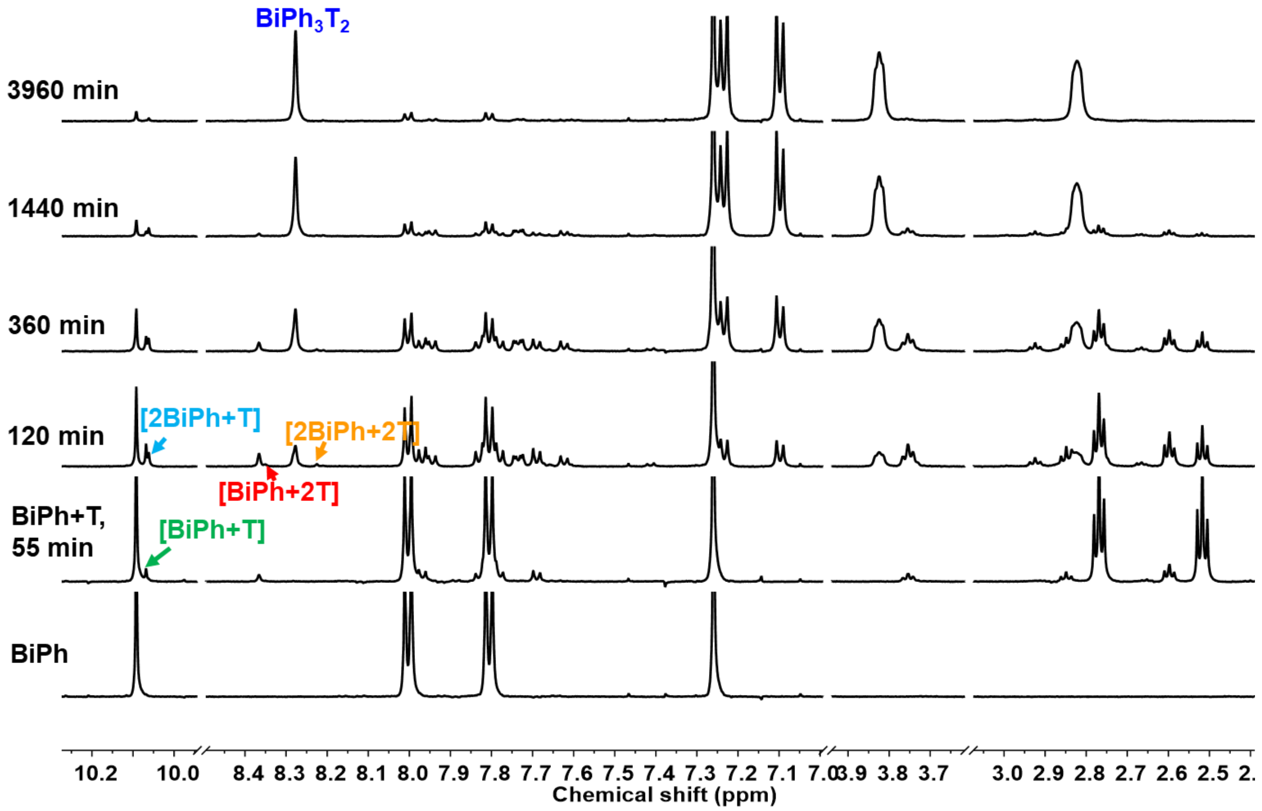

Fig. S28 Temporal evolution of the ^{1}H NMR spectra (500 MHz, $CDCl_3$, 25 °C) of a 3/2 mixture of **BiPh** (3.6 mM) and **T** showing the formation of several intermediates and of the final macrobicyclic cage **BiPh$_3$T$_2$** in dated aluminum treated $CDCl_3$.

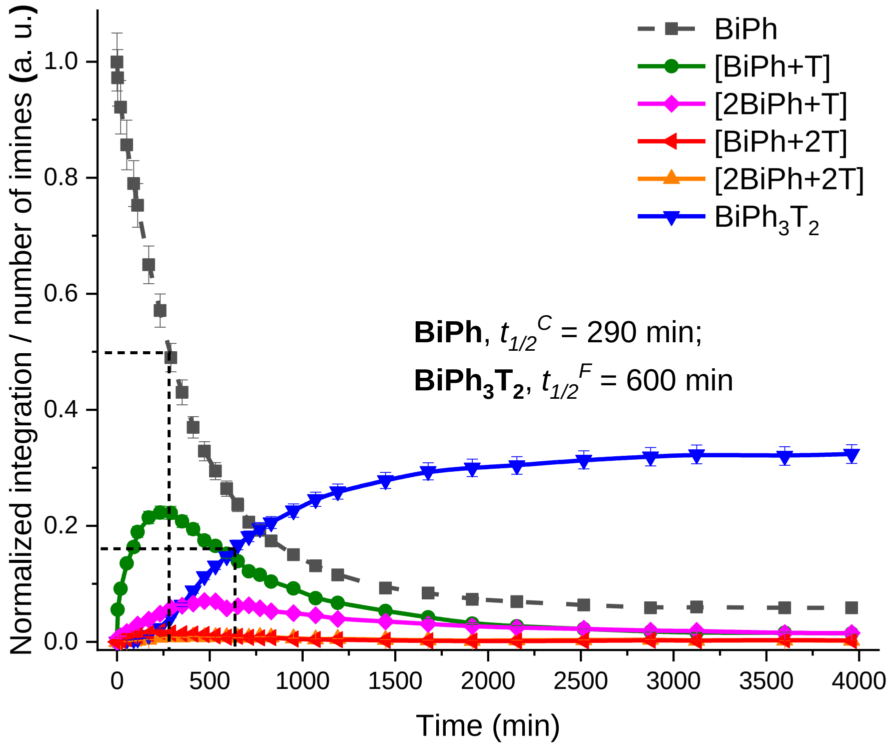

Fig. S29 ^{1}H NMR monitoring of the evolution of cage **BiPh$_3$T$_2$** formation as a function of time over 3960 min. Error in ^{1}H-NMR signal integration: ±5%.

Assignment of [BiPh+2T] and [2BiPh+2T] according to reaction 3BiPh + 4T

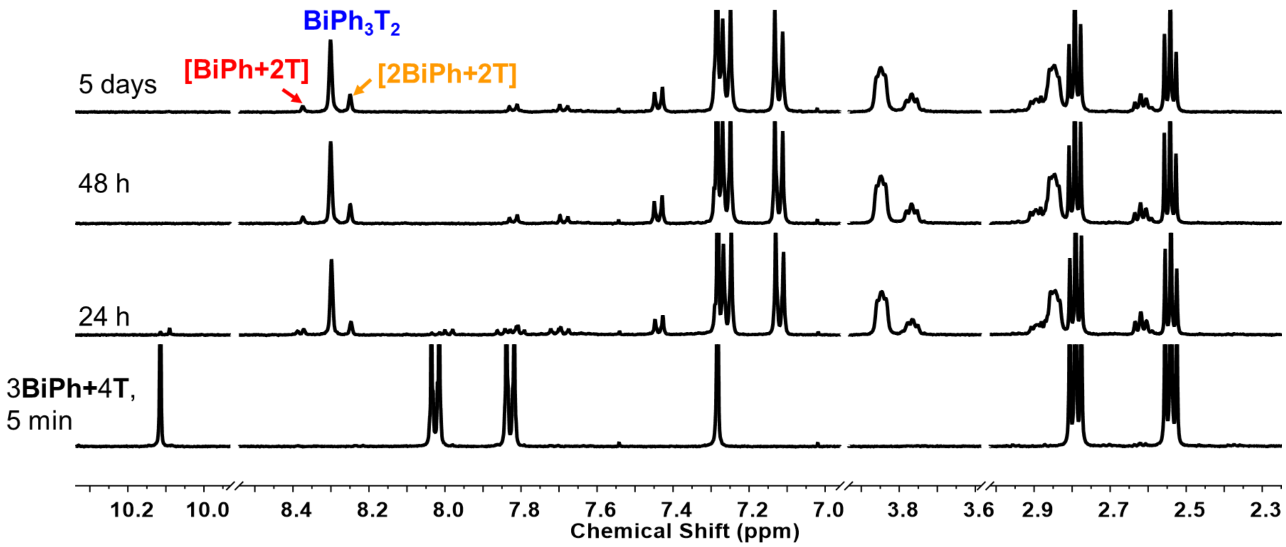

Fig. S30 Temporal evolution of the ^{1}H NMR (400 MHz, $CDCl_3$, 23 °C) spectra of 3**BiPh** + 4**T** reaction in dated aluminum treated $CDCl_3$.

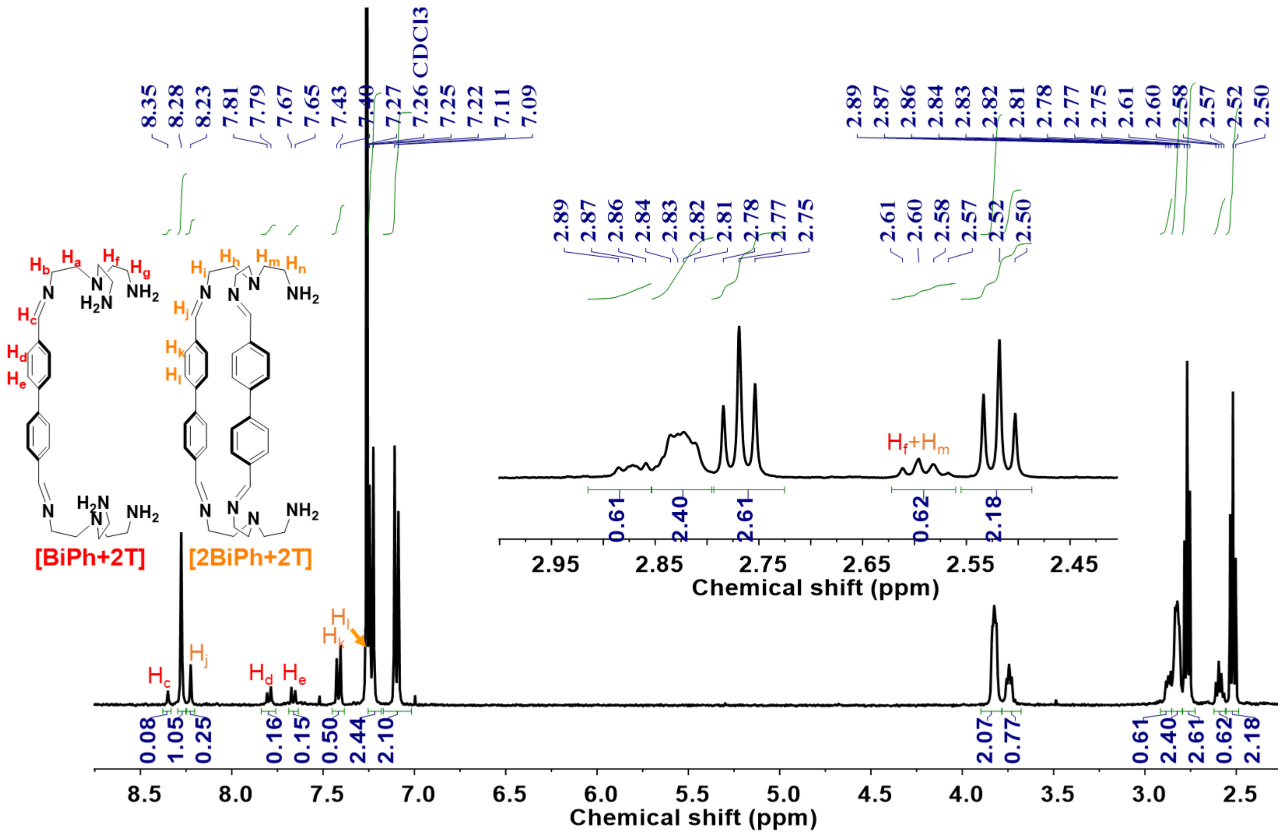

Fig. S31 Equilibrated ^{1}H NMR (400 MHz, $CDCl_3$, 23 °C) spectrum of 3**BiPh** + 4**T** reaction at 48 h.

6.3 Time-dependent ^{1}H NMR of macrobicyclic cage Py_3T_2

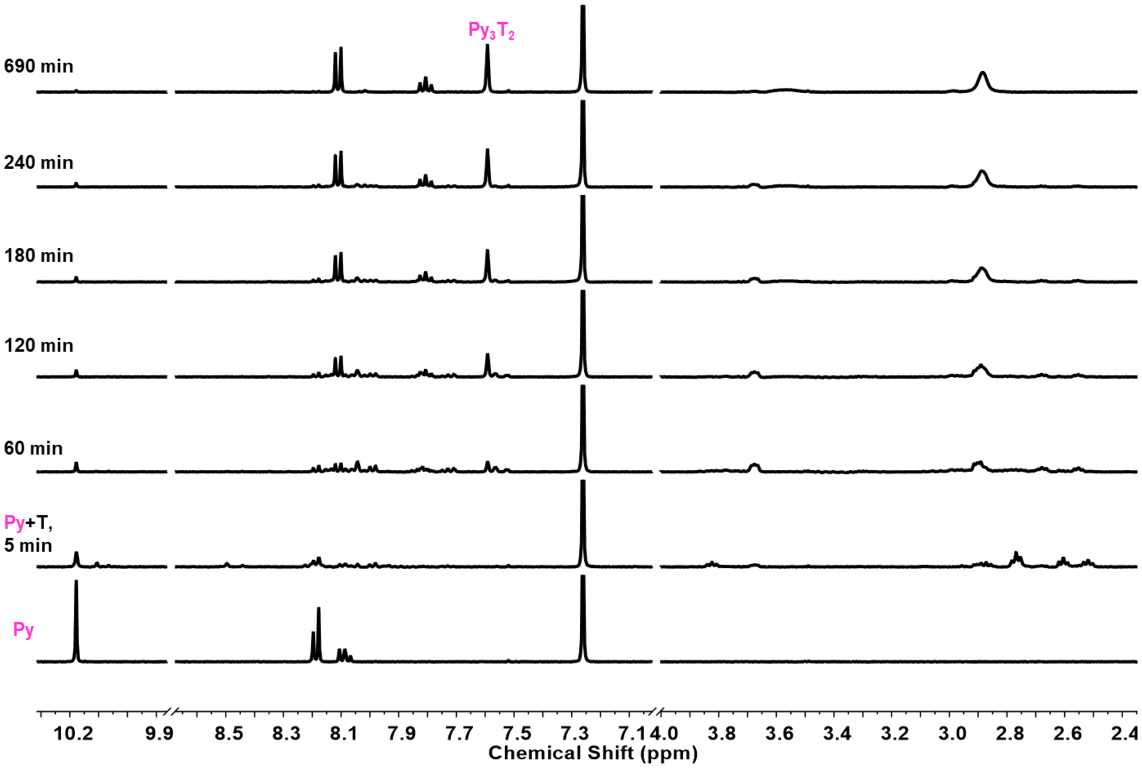

Fig. S32 Temporal evolution of the ^{1}H NMR spectra (400 MHz, $CDCl_3$, 21 °C) of a 3/2 mixture of **Py** (3.6 mM) and **T** showing the formation of several intermediates and of the final macrobicyclic cage **Py_3T_2**.

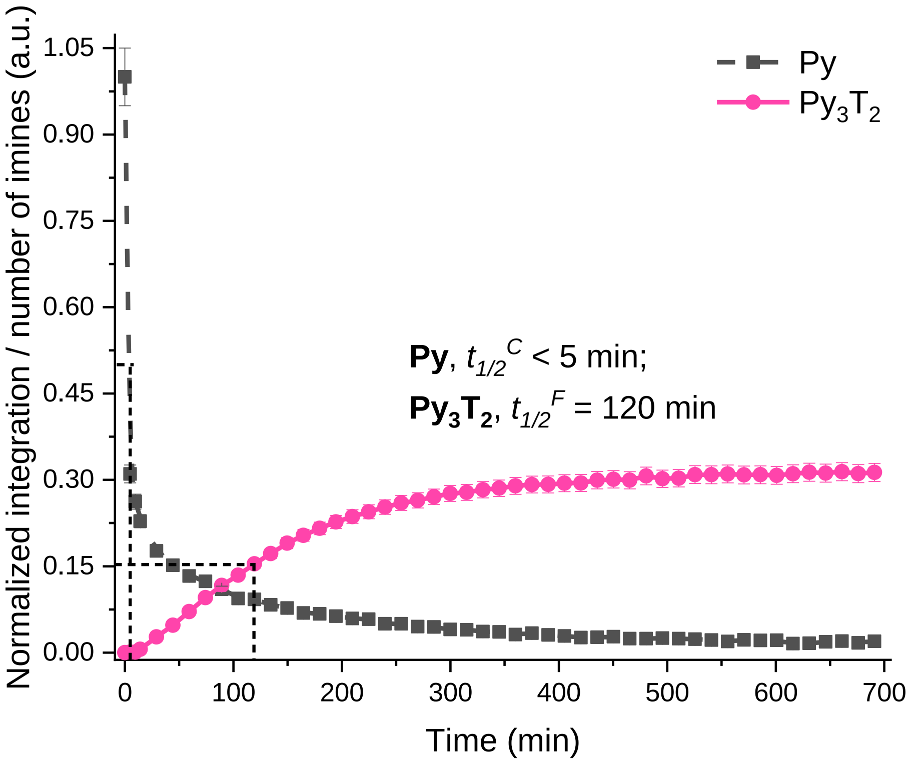

Fig. S33 ^{1}H NMR monitoring of the evolution of cage **Py_3T_2** formation as a function of time over 690 min. Error in ^{1}H-NMR signal integration: ±5%.

6.4 Competitive experiment of 3Py + 3NON + 2T

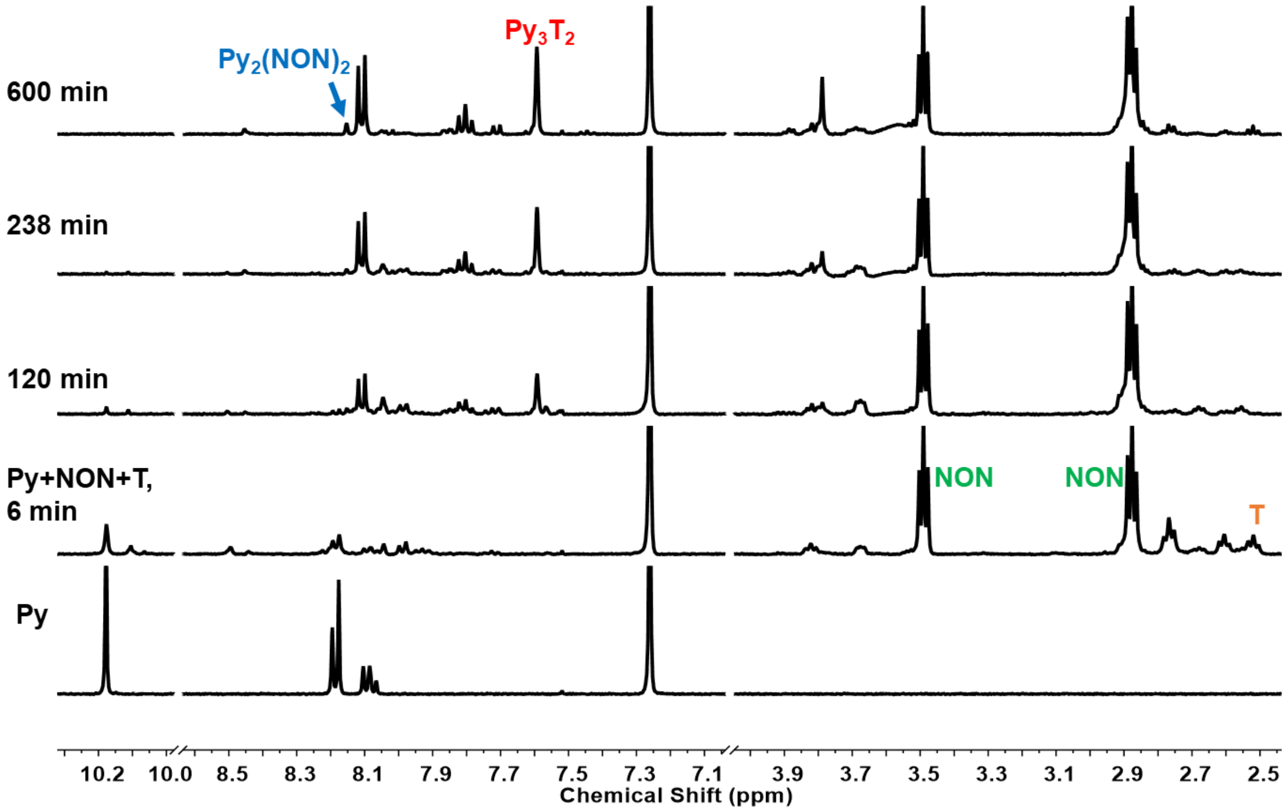

Fig. S34 Temporal evolution of the ^{1}H NMR spectra (400 MHz, $CDCl_3$, 23 °C) of a mixture of **Py** (3.6 mM) with **NON** (3.6 mM) and **T** (2.4 mM) showing the selective formation of macrobicyclic cage **Py₃T₂**.

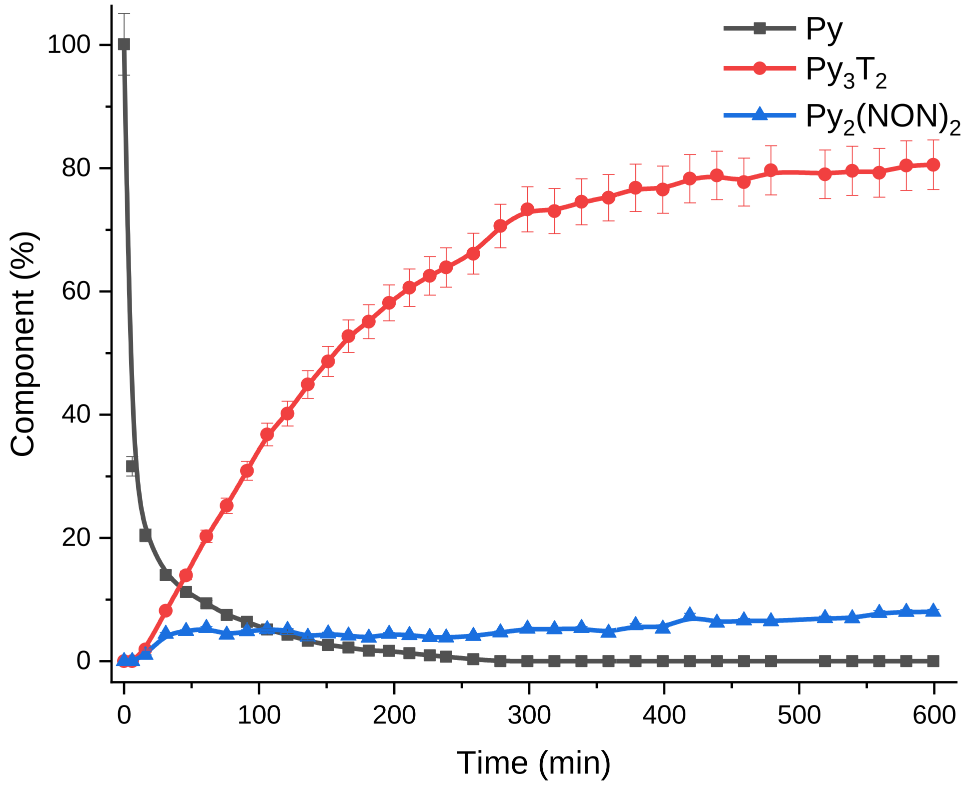

Fig. S35 ^{1}H NMR kinetic profile for the selective formation of cage **Py_3T_2** from the mixture of **Py** (3.6 mM) with **NON** (3.6 mM) and **T** (2.4 mM). Error in ^{1}H-NMR signal integration: ±5%.

6.5 Time-dependent HRMS of macrobicyclic cage pPh_3T_2

Table S3 HRMS-ESI assignment of the Key Species Identified during the formation process of macrocycle **pPh_3T_2**

Entry	assignment	formula	Combined ion	m/z calcd.	m/z found
1	**T**	$C_6H_{18}N_4$	$+H^+$	147.1604	147.1595
			$+Na^+$	169.1424	169.1413
2	[**pPh**+**T**-H_2O]	$C_{14}H_{22}N_4O$	$+H^+$	263.1866	263.1849
			$+Na^+$	285.1686	285.1666
			$+2H^+$	132.0970	N/A
4	[2**pPh**+**T**-2H_2O]	$C_{22}H_{26}N_4O_2$	$+H^+$	379.2129	379.2102
			$+Na^+$	401.1948	401.1921
			$+2H^+$	190.1101	N/A
5	[3**pPh**+**T**-3H_2O]	$C_{30}H_{30}N_4O_3$	$+H^+$	495.2391	495.2358
			$+Na^+$	517.2210	517.2171
			$+2H^+$	248.1232	N/A
3	[**pPh**+2**T**-2H_2O]	$C_{20}H_{38}N_8$	$+H^+$	391.3292	391.3265
			$+Na^+$	413.3112	413.3083
			$+2H^+$	196.1682	196.1670
6	[2**pPh**+2**T**-3H_2O]	$C_{28}H_{42}N_8O$	$+H^+$	507.3554	N/A
			$+Na^+$	529.3374	N/A
			$+2H^+$	254.1814	N/A
7	[2**pPh**+2**T**-4H_2O]	$C_{28}H_{40}N_8$	$+H^+$	489.3449	489.3415
			$+Na^+$	511.3268	511.3236
			$+2H^+$	245.1761	245.1744
8	[3**pPh**+2**T**-4H_2O]	$C_{36}H_{46}N_8O_2$	$+H^+$	623.3816	N/A
			$+Na^+$	645.3636	N/A
			$+2H^+$	312.1945	N/A
9	[3**pPh**+2**T**-5H_2O]	$C_{36}H_{44}N_8O$	$+H^+$	605.3711	605.3671
			$+Na^+$	627.3491	627.3530
			$+2H^+$	303.1892	303.1869
10	[3**pPh**+2**T**-6H_2O]	$C_{36}H_{42}N_8$	$+H^+$	587.3605	587.3568
	Cage **pPh_3T_2**		$+Na^+$	609.3425	609.3388
			$+2H^+$	294.1839	294.1820

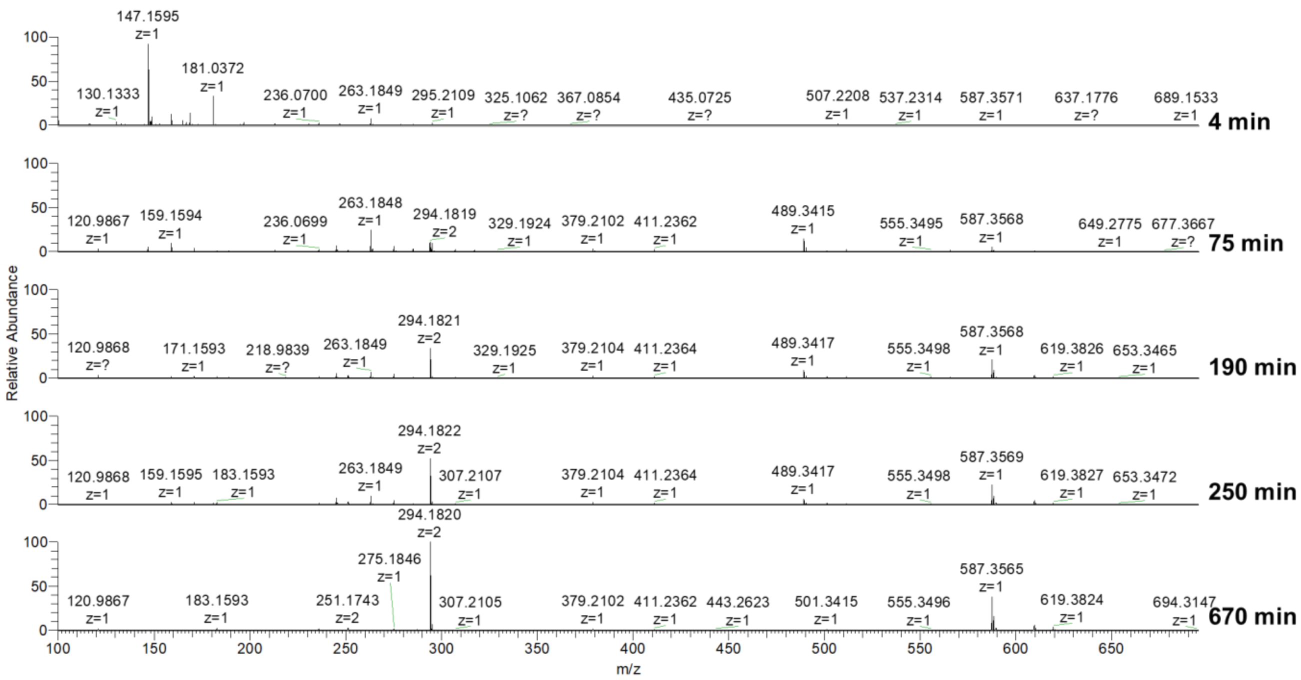

Fig. S36 Temporal evolution of the HRMS-ESI spectra of the 3/2 mixture of **pPh** (2 mM) and **T** in 50%-50% $CHCl_3$/MeOH.

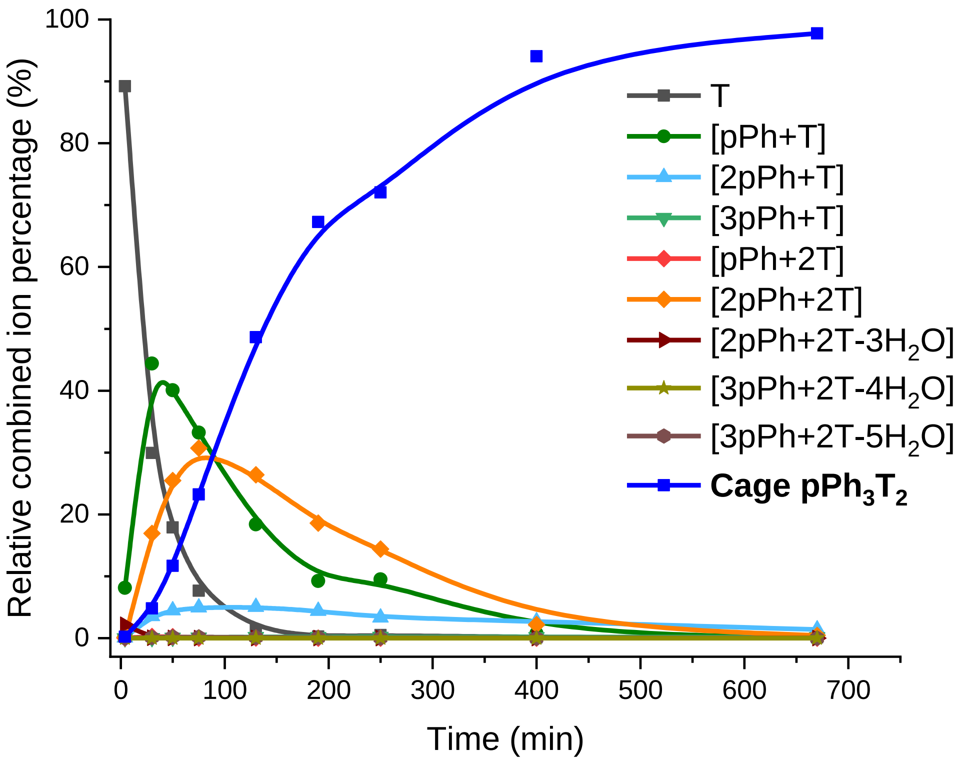

Fig. S37 HRMS-ESI kinetic evolution of the species generated during the formation of the macrobicyclic cage **pPh$_3$T$_2$** from **pPh** (2 mM) and **T** (50%-50% $CHCl_3$/MeOH, r.t) as a function of time over 670 min. NB: The relative combined ion percentage is obtained by the ratio of combined ion count of each species to the sum of combined ion counts for all species at each time point. These data do not provide quantitative information about the relative amounts of each species identified by its mass, but, taken separately, they display the evolution of a given identified species during the course of the reaction. The curves are added to guide the eye.

6.6 Time-dependent HRMS of macrobicyclic cage BiPh$_3$T$_2$

Table S4 HRMS-ESI assignment of the Key Species Identified during the formation process of macrocycle **BiPh$_3$T$_2$**

Entry	assignment	formula	Combined ion	m/z calcd.	m/z found
1	**T**	$C_6H_{18}N_4$	$+H^+$	147.1604	147.1595
			$+Na^+$	169.1424	169.1413
2	[**BiPh**+**T**-H_2O]	$C_{20}H_{26}N_4O$	$+H^+$	339.2179	339.2156
			$+Na^+$	361.1999	361.1974
			$+2H^+$	170.1126	N/A
4	[2**BiPh**+**T**-2H_2O]	$C_{34}H_{34}N_4O_2$	$+H^+$	531.2755	531.2720
			$+Na^+$	553.2574	553.2539
			$+2H^+$	266.1414	266.1407
5	[3**BiPh**+**T**-3H_2O]	$C_{48}H_{42}N_4O_3$	$+H^+$	723.3330	723.3275
			$+Na^+$	745.3149	745.3104
			$+2H^+$	362.1701	N/A
3	[**BiPh**+2**T**-2H_2O]	$C_{26}H_{42}N_8$	$+H^+$	467.3605	N/A
			$+Na^+$	489.3425	489.3366
			$+2H^+$	234.1839	234.1824
6	[2**BiPh**+2**T**-3H_2O]	$C_{40}H_{50}N_8O$	$+H^+$	659.4180	N/A
			$+Na^+$	681.4000	N/A
			$+2H^+$	330.2127	N/A
7	[2**BiPh**+2**T**-4H_2O]	$C_{40}H_{48}N_8$	$+H^+$	641.4075	641.4027
			$+Na^+$	663.3844	663.3894
			$+2H^+$	321.2052	321.2074
8	[3**BiPh**+2**T**-4H_2O]	$C_{54}H_{58}N_8O_2$	$+H^+$	851.4755	N/A
			$+Na^+$	873.4575	N/A
			$+2H^+$	426.2414	N/A
9	[3**BiPh**+2**T**-5H_2O]	$C_{54}H_{56}N_8O$	$+H^+$	833.4650	833.4509
			$+Na^+$	855.4469	855.4075
			$+2H^+$	417.2361	517.2229
10	[3**BiPh**+2**T**-6H_2O]	$C_{54}H_{54}N_8$	$+H^+$	815.4544	815.4484
	Cage **BiPh$_3$T$_2$**		$+Na^+$	837.4364	837.4302
			$+2H^+$	408.2308	408.2283

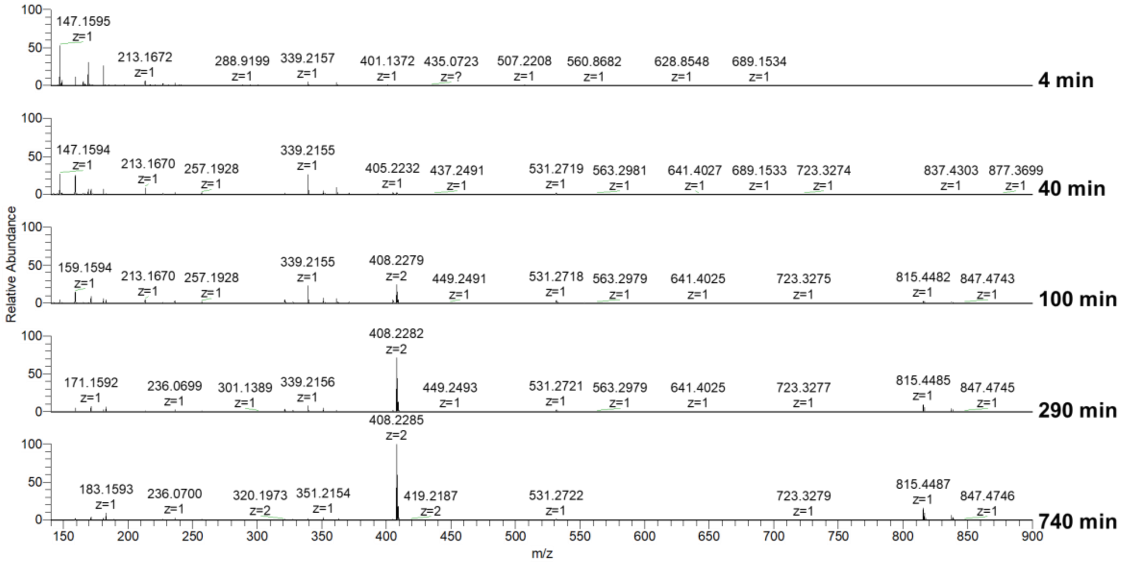

Fig. S38 Temporal evolution of the HRMS-ESI spectra of the 3/2 mixture of **BiPh** (2 mM) and **T** in 50%-50% $CHCl_3$/MeOH.

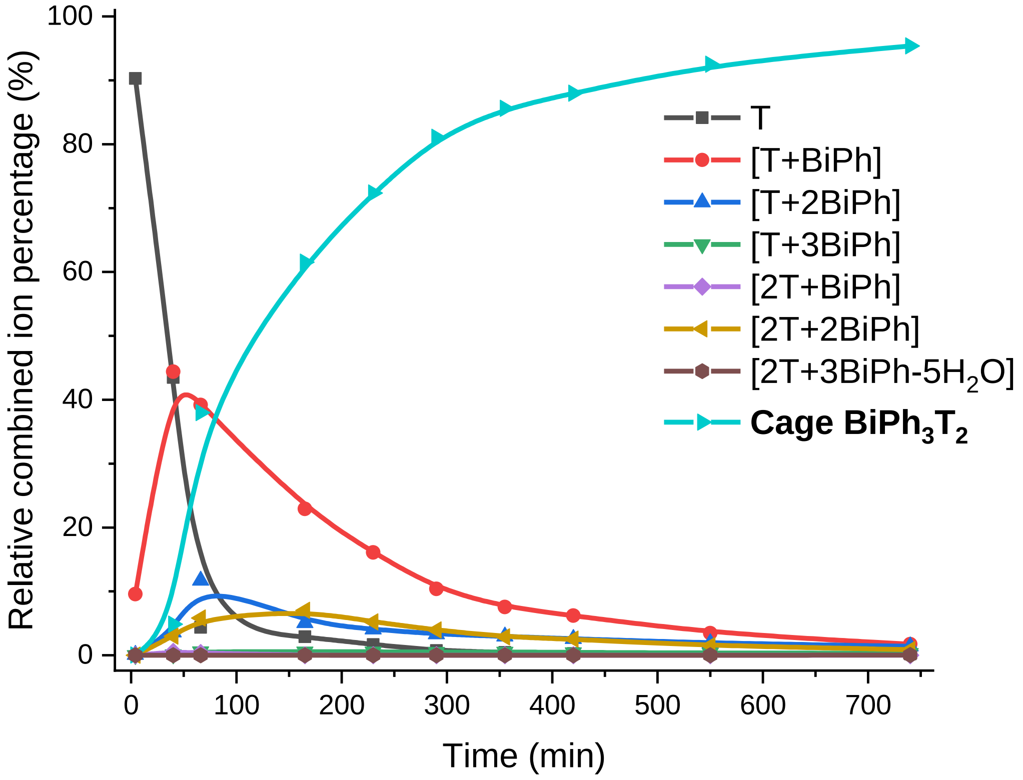

Fig. S39 HRMS-ESI kinetic evolution of the species generated during the formation of the macrocycle $\mathbf{BiPh_3T_2}$ from **BiPh** (2 mM) and **T** (50%-50% $CHCl_3$/MeOH, r.t) as a function of time over 740 min. NB: The relative combined ion percentage is obtained by the ratio of combined ion count of each species to the sum of combined ion counts for all species at each time point. These data do not provide quantitative information about the relative amounts of each species identified by its mass, but, taken separately, they display the evolution of a given identified species during the course of the reaction. The curves are added to guide the eye.

7. Self-sorting experiments

7.1 Self-sorting experiment 2pPh + 2BiPh + 4NON

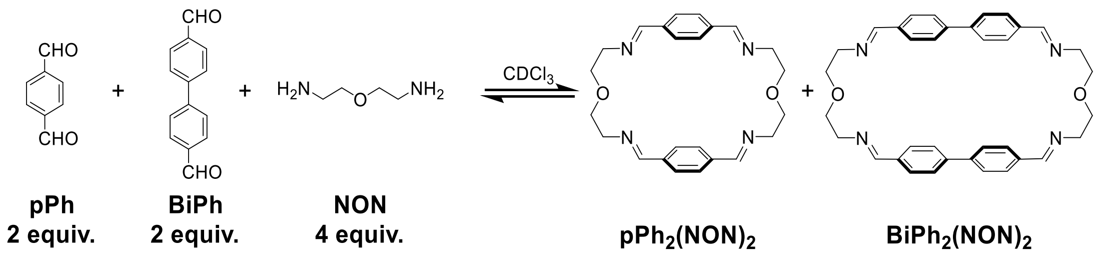

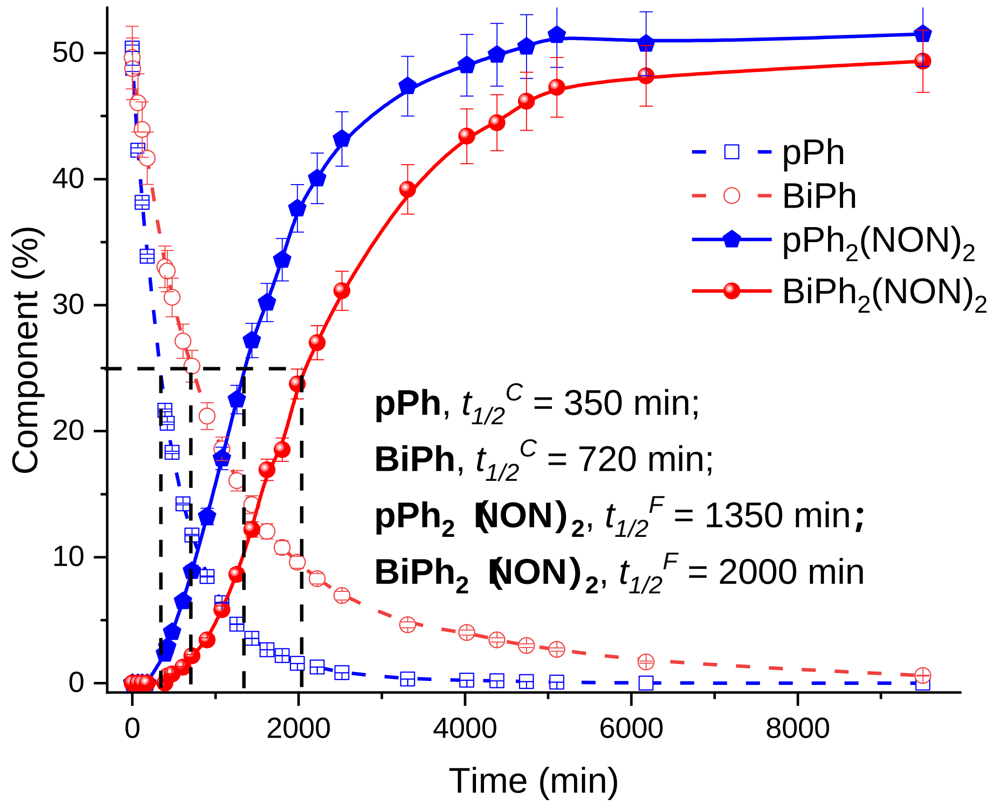

Fig. S40 ^{1}H NMR monitoring during the self-sorting of macrocycle **pPh$_2$(NON)$_2$** and **BiPh$_2$(NON)$_2$** with fresh aluminum treated $CDCl_3$. Error in ^{1}H-NMR signal integration: ±5%.

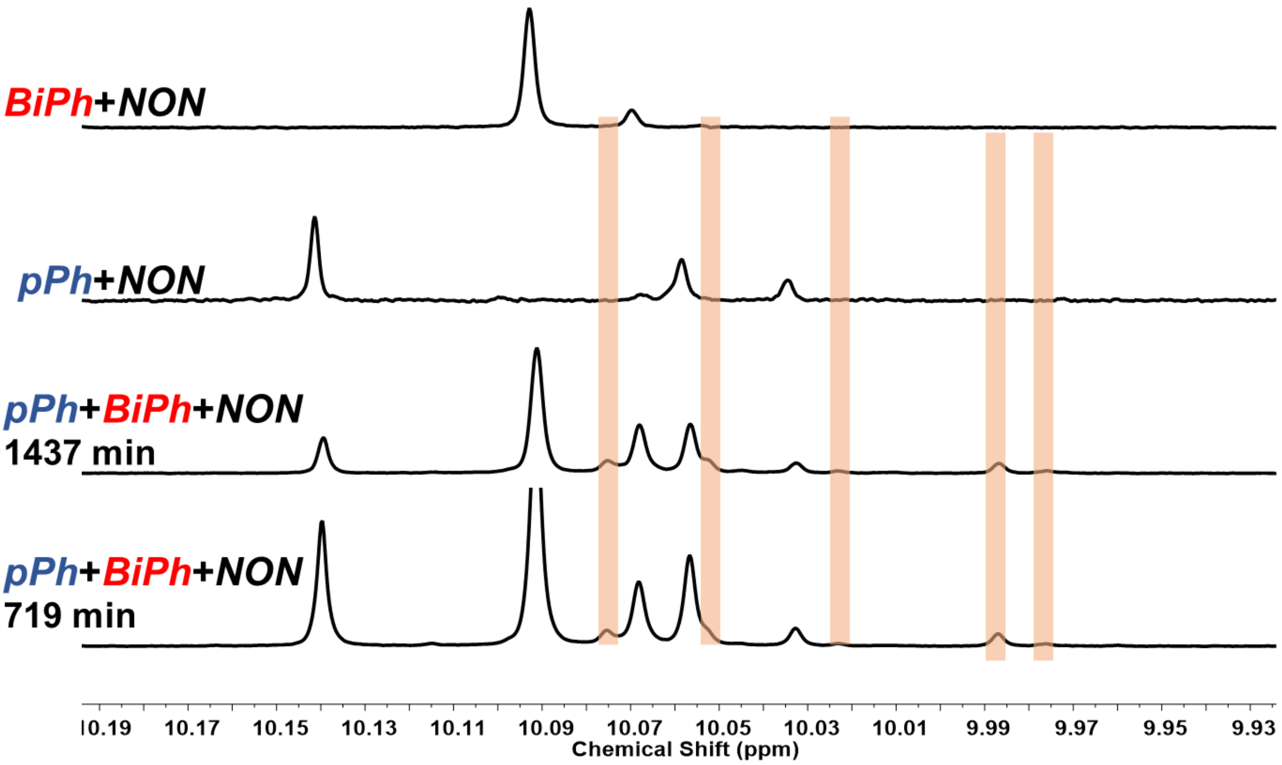

Fig. S41 ^{1}H NMR spectra comparison of **pPh/BiPh/NON** self-sorting reaction with **pPh/NON** and **BiPh/NON** separated reactions. The top two traces correspond to the **pPh/NON** reaction at 2880 min and **BiPh/NON** reaction at 4384 min.

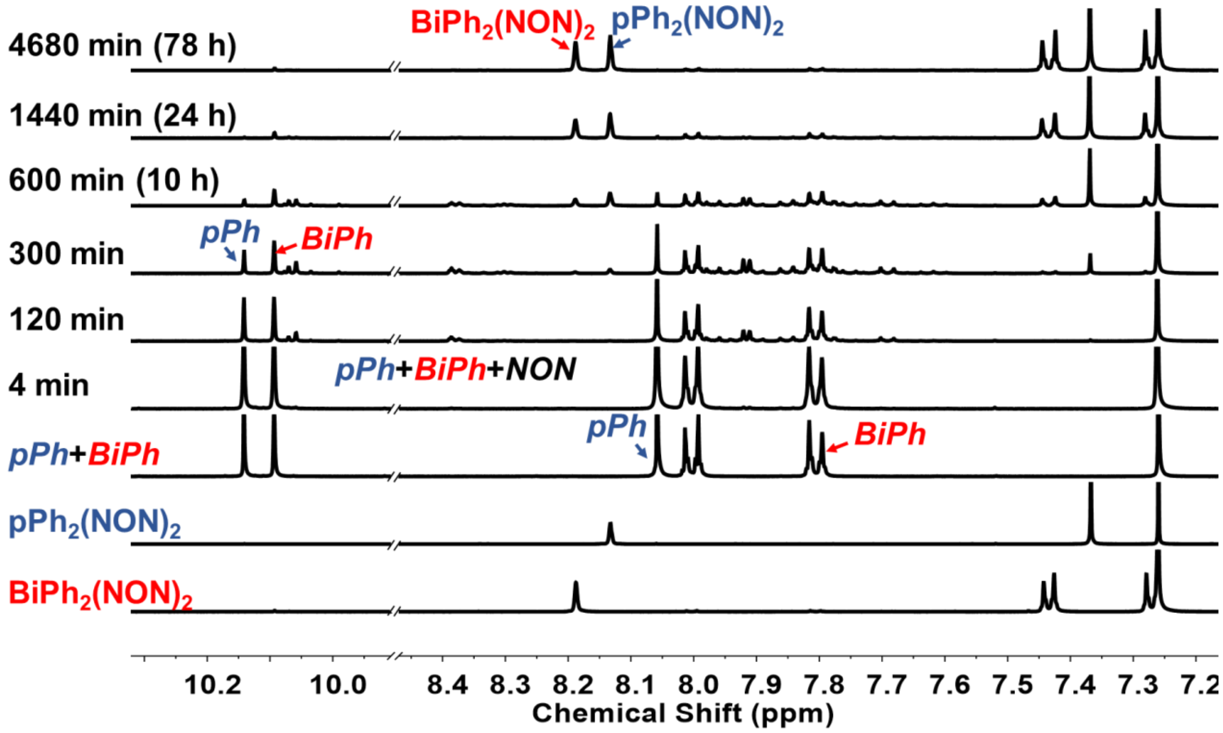

Fig. S42 Temporal evolution of the ^{1}H NMR spectra (400 MHz, $CDCl_3$, 23 °C) during the self-sorting of macrocycle **pPh$_2$(NON)$_2$** and **BiPh$_2$(NON)$_2$** in dated aluminum treated $CDCl_3$.

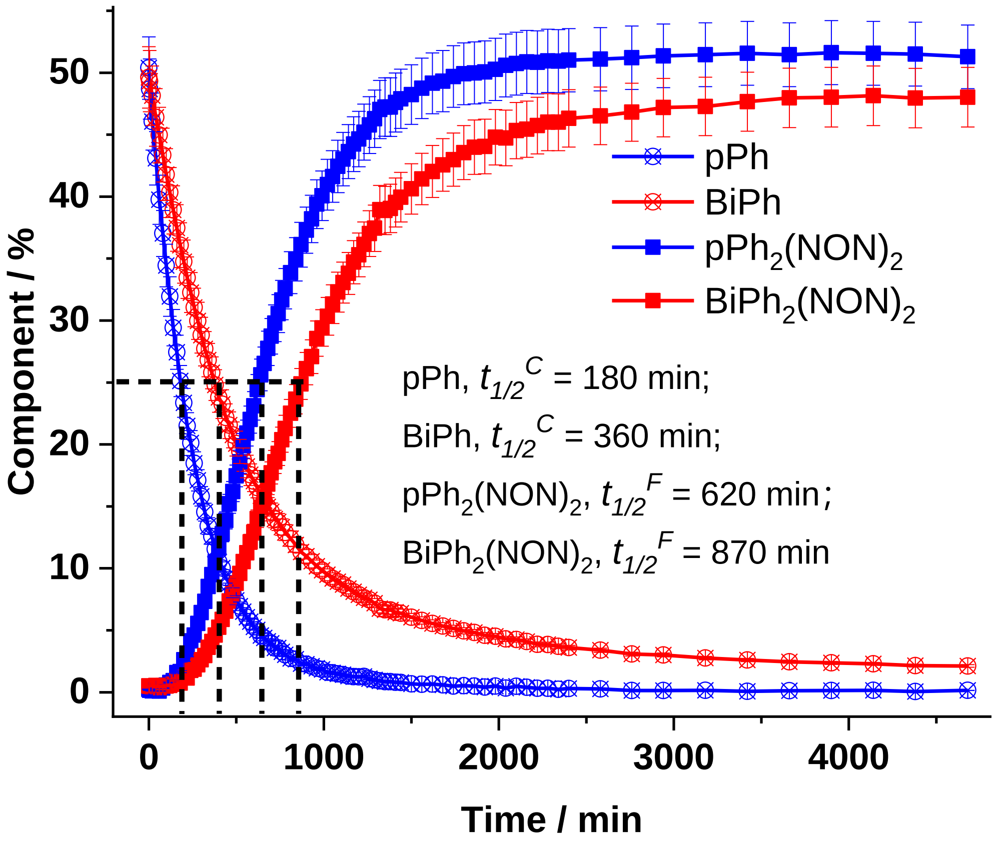

Fig. S43 ^{1}H NMR monitoring during the self-sorting of macrocycle **pPh₂(NON)₂** and **BiPh₂(NON)₂** in dated aluminum treated $CDCl_3$. Error in ^{1}H-NMR signal integration: ±5%.

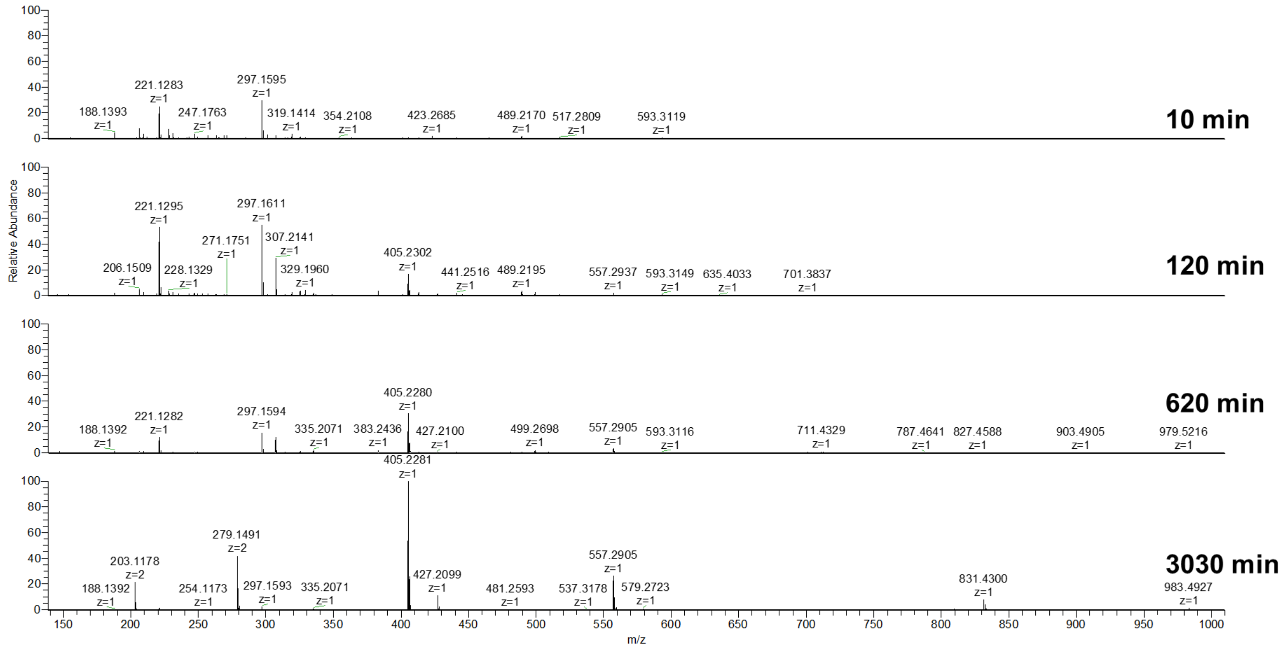

Fig. S44 Temporal evolution of HRMS-ESI spectra of the 2/2/4 mixture of **pPh** (2 mM), **BiPh** (2 mM) and **NON** in 50%-50% $CHCl_3$/MeOH.

Table S5 HRMS-ESI assignments of the Key Species Identified during the self-sorting reaction of 2**pPh** + 2**BiPh** + 4**NON**

Entry	assignment	formula	Combined ion	m/z calcd.	m/z found
1	[**pPh**+**NON**-H_2O]	$C_{12}H_{16}N_2O_2$	$+H^+$	221.1285	221.1283
			$+Na^+$	243.1104	243.1103
2	[**pPh**+2**NON**-2H_2O]	$C_{16}H_{26}N_4O_2$	$+H^+$	307.2129	307.2125
			$+Na^+$	329.1948	329.1967
			$+2H^+$	154.1101	154.1100
3	[2**pPh**+**NON**-2H_2O]	$C_{20}H_{20}N_2O_3$	$+H^+$	337.1547	337.1542
			$+Na^+$	359.1372	359.1362
			$+2H^+$	169.0810	N/A
4	[2**pPh**+2**NON**-3H_2O]	$C_{24}H_{30}N_4O_3$	$+H^+$	423.2391	423.2386
			$+Na^+$	445.2210	445.2118
			$+2H^+$	212.1232	212.1392
5	[2**pPh**+2**NON**-4H_2O] **$pPh_2(NON)_2$**	$C_{24}H_{28}N_4O_2$	$+H^+$	405.2285	405.2279
			$+Na^+$	427.2104	427.2099
			$+2H^+$	203.1179	203.1176
6	[**BiPh**+**NON**-H_2O]	$C_{18}H_{20}N_2O_2$	$+H^+$	297.1598	297.1593
			$+Na^+$	319.1417	319.1410
			$+2H^+$	149.0835	149.0834
7	[**BiPh**+2**NON**-2H_2O]	$C_{22}H_{30}N_4O_2$	$+H^+$	383.2442	N/A
			$+Na^+$	405.2261	405.2281
			$+2H^+$	192.1257	192.1254
8	[2**BiPh**+**NON**-2H_2O]	$C_{32}H_{28}N_2O_3$	$+H^+$	489.2173	489.2166
			$+Na^+$	511.1992	511.2074
			$+2H^+$	245.1123	N/A
9	[2**BiPh**+2**NON**-3H_2O]	$C_{36}H_{38}N_4O_3$	$+H^+$	575.3017	N/A
			$+Na^+$	597.2836	N/A
			$+2H^+$	288.1545	N/A
10	[2**BiPh**+2**NON**-4H_2O] **$BiPh_2(NON)_2$**	C36H36N4O2	$+H^+$	557.2911	557.2904
			$+Na^+$	579.2730	579.2722
			$+2H^+$	279.1492	279.1490
11	[**pPh**+**BiPh**+**NON**-2H_2O]	$C_{26}H_{24}N_2O_3$	$+H^+$	413.1860	413.1852
			$+Na^+$	435.1679	435.1672
			$+2H^+$	207.0966	N/A
12	[**pPh**+**BiPh**+2**NON**-3H_2O]	$C_{30}H_{34}N_4O_3$	$+H^+$	499.2704	499.2697
			$+Na^+$	521.2523	N/A
			$+2H^+$	250.1388	250.1384
13	[**pPh**+**BiPh**+2**NON**-4H_2O] **(pPh)(BiPh)$(NON)_2$**	$C_{30}H_{32}N_4O_2$	$+H^+$	481.2598	481.2592
			$+Na^+$	503.2417	503.2413
			$+2H^+$	241.1335	241.1331

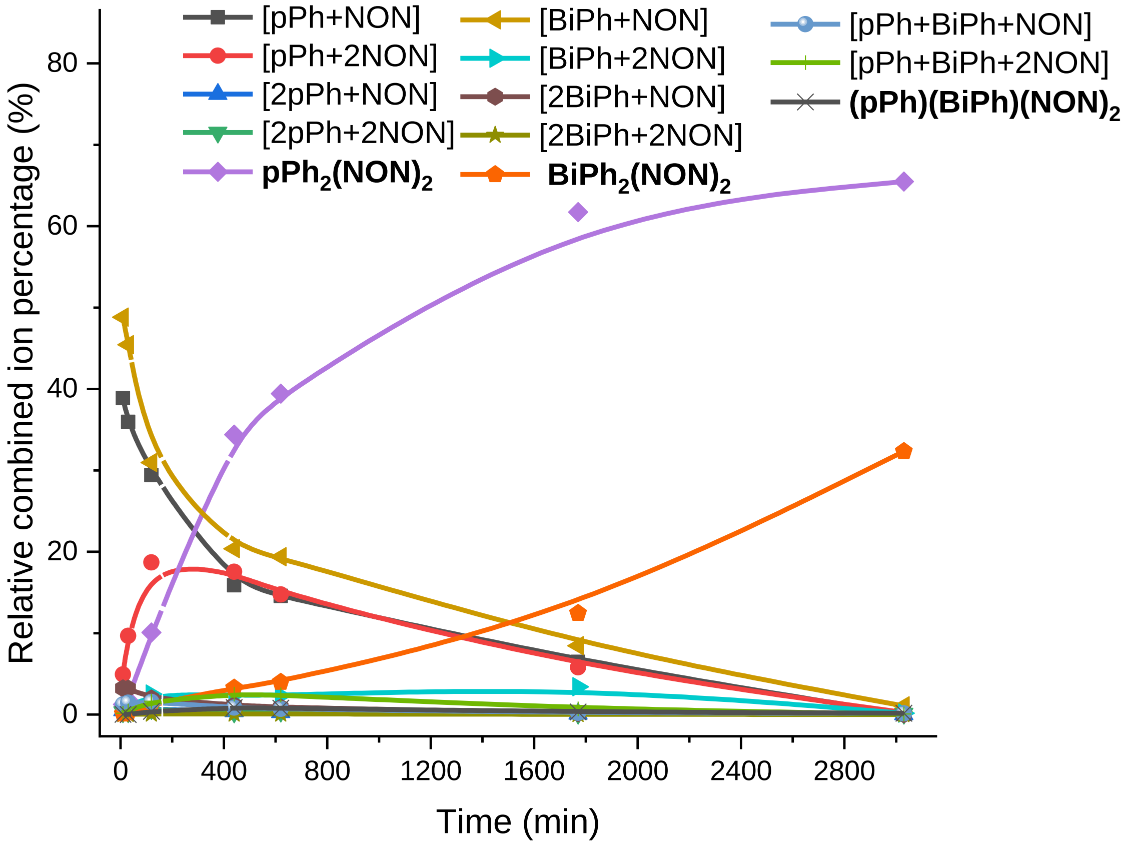

Fig. S45. HRMS-ESI kinetic evolution of the species generated during the during the self-sorting process of 2**pPh** + 2**BiPh** + 4**NON** (50%-50% $CHCl_3$/MeOH, r.t) as a function of time over 1440 min. NB: The relative combined ion percentage is obtained by the ratio of combined ion count of each species to the sum of combined ion counts for all species at each time point. These data do not provide quantitative information about the relative amounts of each species identified by its mass, but, taken separately, they display the evolution of a given identified species during the course of the reaction. The curves are added to guide the eye.

Table S6. HRMS-ESI intensity comparison of heteroleptic macrocycle **(pPh)(BiPh)(NON)$_2$** with homoleptic macrocycles

Time (min)	I_1 (a.u.)	I_2 (a.u.)	I_3 (a.u.)	I_3/avg. (I_1+I_2) *100 (%)
10	1.86E+07	1.49E+06	0	0.00
30	3.81E+07	5.10E+06	180494	0.83
120	4.06E+08	4.99E+07	1.61E+07	7.08
180	7.00E+08	7.16E+07	2.47E+07	6.41
500	6.75E+08	6.36E+07	1.59E+07	4.29
620	6.98E+08	7.06E+07	1.41E+07	3.68
750	7.32E+08	8.20E+07	1.30E+07	3.19
1440	7.08E+08	1.03E+08	6.96E+06	1.72

I_1: combined ion count of **BiPh$_2$(NON)$_2$**

I_2: combined ion count of **pPh$_2$(NON)$_2$**

I_3: combined ion count of **(pPh)(BiPh)(NON)$_2$**

7.2 Self-sorting experiment 3pPh + 3BiPh + 4T

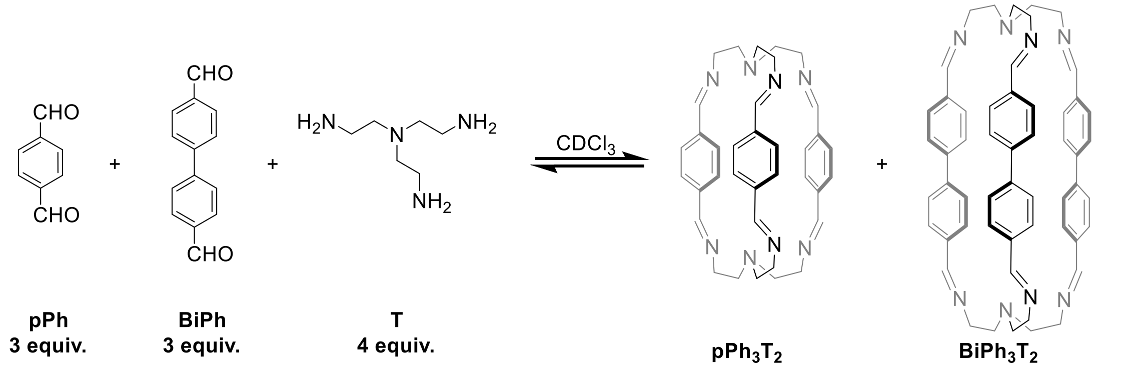

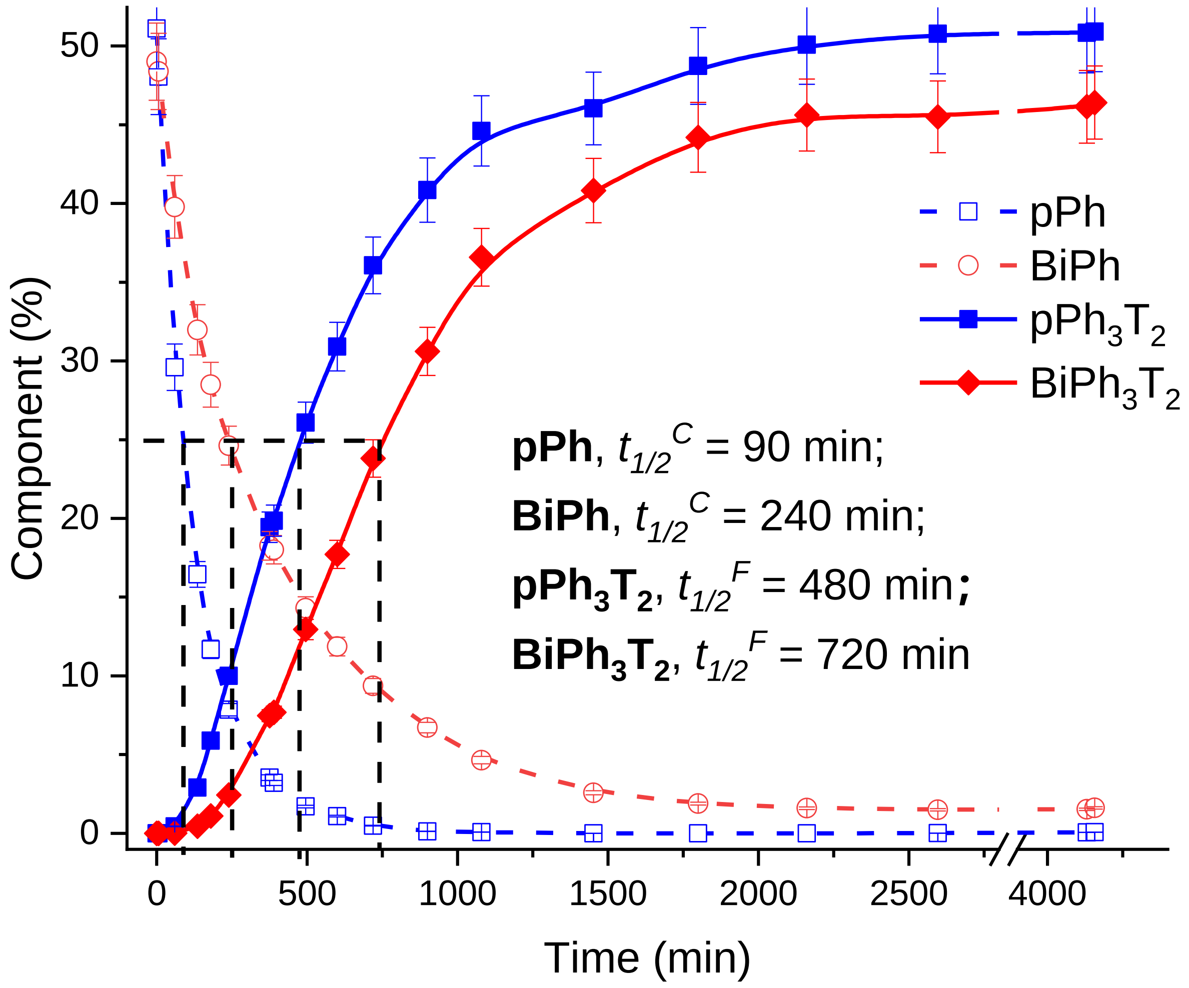

Fig. S46 ^{1}H NMR monitoring during the self-sorting of **pPh_3T_2** and **$BiPh_3T_2$** in fresh aluminum treated $CDCl_3$. Error in ^{1}H-NMR signal integration: ±5%.

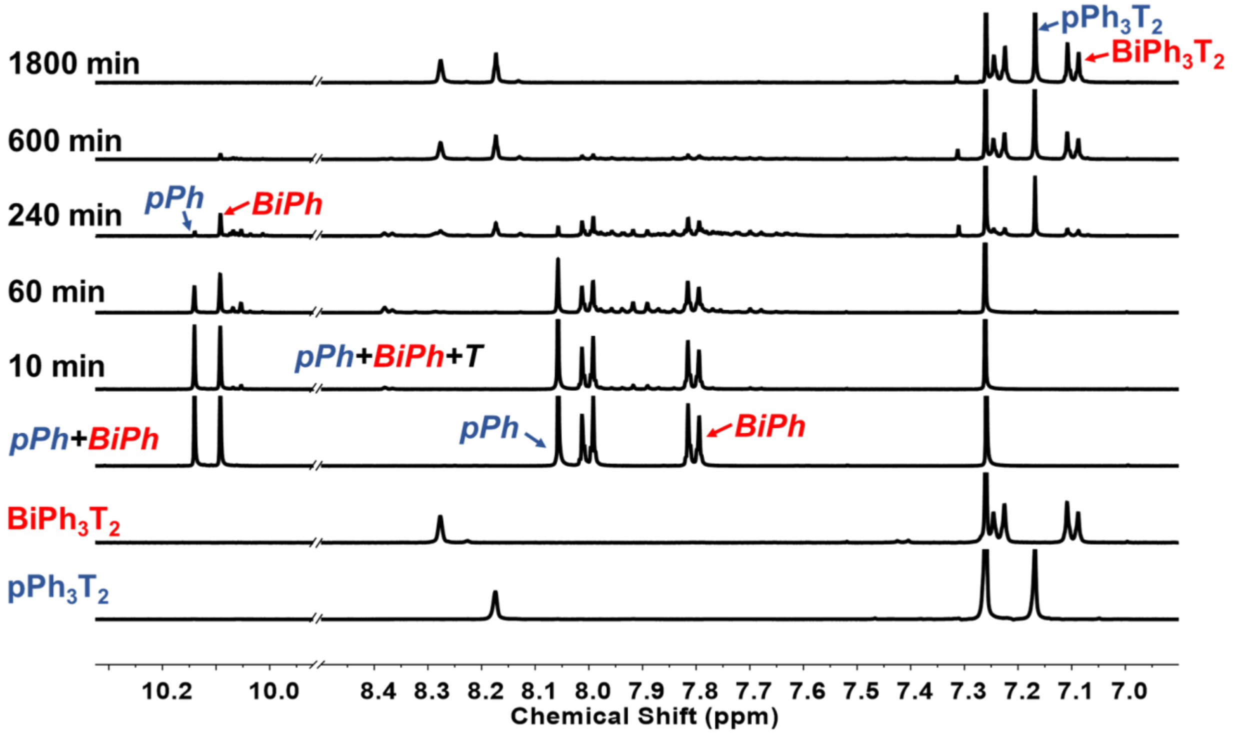

Fig. S47 Temporal evolution of the ^{1}H NMR spectra (400 MHz, $CDCl_3$, 23 °C) during the self-sorting of **pPh_3T_2** and **$BiPh_3T_2$** in dated aluminum treated $CDCl_3$.

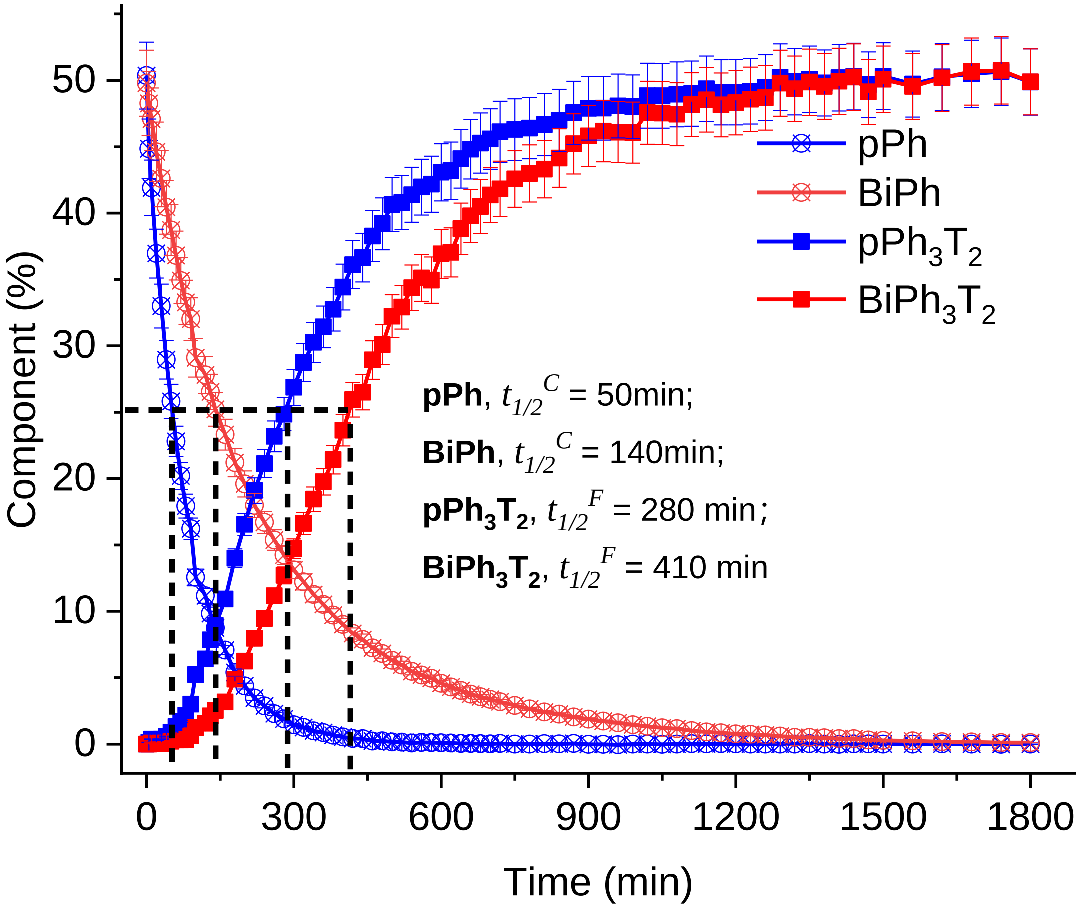

Fig. S48 ^{1}H NMR monitoring during the self-sorting of **pPh_3T_2** and **$BiPh_3T_2$** in dated aluminum treated $CDCl_3$. Error in ^{1}H-NMR signal integration: ±5%.

Table S7 HRMS-ESI assignments of the Key Species Identified during the self-sorting reaction of 3**pPh** + 3**BiPh** + 4**T**.

Entry	assignment	formula	Combined ion	m/z calcd.	m/z found
1	**T**	C6H18N4	$+H^+$	147.1604	147.1597
			$+Na^+$	169.1424	169.1415
2	[**pPh**+**T**-H_2O]	C14H22N4O	$+H^+$	263.1866	263.1852
			$+Na^+$	285.1686	285.1671
			$+2H^+$	132.0970	N/A
3	[2**pPh**+**T**-2H_2O]	C22H26N4O2	$+H^+$	379.2129	379.2100
			$+Na^+$	401.1948	401.1924
			$+2H^+$	190.1101	N/A
4	[3**pPh**+**T**-3H_2O]	C30H30N4O3	$+H^+$	495.2391	495.2362
			$+Na^+$	517.2210	N/A
			$+2H^+$	248.1232	N/A
5	[**pPh**+2**T**-2H_2O]	C20H38N8	$+H^+$	391.3292	391.3262
			$+Na^+$	413.3112	413.3083
			$+2H^+$	196.1682	196.1671
6	[2**pPh**+2**T**-4H_2O]	C28H40N8	$+H^+$	489.3449	489.3417
			$+Na^+$	511.3268	511.3236
			$+2H^+$	245.1761	245.1745
7	[3**pPh**+2**T**-6H_2O] Cage **pPh$_3$T$_2$**	C36H42N8	$+H^+$	587.3605	587.3567
			$+Na^+$	609.3425	609.3391
			$+2H^+$	294.1839	294.1820
8	[**BiPh**+**T**-H_2O]	C20H26N4O	$+H^+$	339.2179	339.2156
			$+Na^+$	361.1999	361.1974
			$+2H^+$	170.1126	N/A
9	[2**BiPh**+**T**-2H_2O]	C34H34N4O2	$+H^+$	531.2755	531.2723
			$+Na^+$	553.2574	553.2543
			$+2H^+$	266.1414	N/A
10	[3**BiPh**+**T**-3H_2O]	C48H42N4O3	$+H^+$	723.3330	723.3093
			$+Na^+$	745.3149	N/A
			$+2H^+$	362.1701	362.1998
11	[**BiPh**+2**T**-2H_2O]	C26H42N8	$+H^+$	467.3605	467.3576
			$+Na^+$	489.3425	N/A[a]
			$+2H^+$	234.1839	234.1827
12	[2**BiPh**+2**T**-4H_2O]	C40H48N8	$+H^+$	641.4075	641.4045
			$+Na^+$	663.3844	663.3864
			$+2H^+$	321.2052	321.2054
13	[3**BiPh**+2**T**-6H_2O] Cage **BiPh$_3$T$_2$**	C54H54N8	$+H^+$	815.4544	815.4494
			$+Na^+$	837.4364	837.4312
			$+2H^+$	408.2308	408.2308
14	[**pPh**+**BiPh**+**T**-2H_2O]	C28H30N4O2	$+H^+$	455.2442	455.2415
			$+Na^+$	477.2261	477.2226
			$+2H^+$	228.1257	N/A
15	[2**pPh**+**BiPh**+**T**-3H_2O]	C36H34N4O3	$+H^+$	571.2704	571.2659
			$+Na^+$	593.2523	N/A
			$+2H^+$	286.1388	N/A

16	[**pPh**+2**BiPh**+**T**-3H_2O]	C42H38N4O3	+H^+	647.3017	647.2968
			+Na^+	669.2836	N/A
			+2H^+	324.1545	N/A
17	[**pPh**+**BiPh**+2**T**-3H_2O]	C34H46N8O	+H^+	583.3867	583.3823
			+Na^+	605.3687	605.3699
			+2H^+	292.1970	292.1949
18	[**pPh**+**BiPh**+2**T**-4H_2O]	C34H44N8	+H^+	565.3762	565.3561
			+Na^+	587.3581	N/A[a]
			+2H^+	283.1917	283.1897
19	[2**pPh**+**BiPh**+2**T**-5H_2O]	C42H48N8O	+H^+	681.4024	681.3974
			+Na^+	703.3843	7.3.3795
			+2H^+	341.2048	341.2025
20	[**pPh**+2**BiPh**+2**T**-4H_2O]	C48H54N8O2	+H^+	775.4442	N/A
			+Na^+	797.4262	797.4228
			+2H^+	388.2258	388.2229
21	[**pPh**+2**BiPh**+2**T**-5H_2O]	C48H52N8O	+H^+	757.4337	757.4284
			+Na^+	779.4156	779.4086
			+2H^+	379.2205	379.2188
22	[**pPh**+2**BiPh**+2**T**-6H_2O] Cage**(pPh)(BiPh)$_2$T$_2$**	C48H50N8	+H^+	739.4231	739.4170
			+Na^+	761.4051	761.3801
			+2H^+	370.2152	370.2128

a. Unable to read the mass-to-charge ratio (m/z) abundance as it possesses extremely close m/z value with other highly responded ion-combined species.

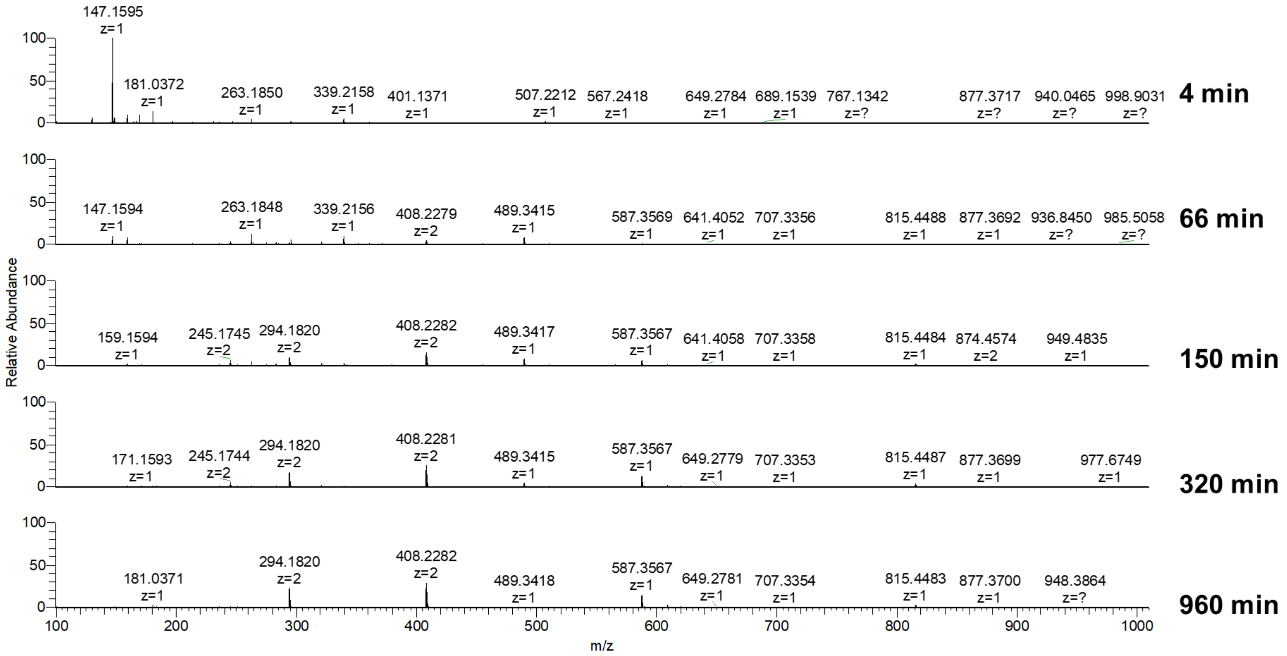

Fig. S49 Temporal evolution of the HRMS-ESI spectra of the 3/3/4 mixture of **pPh** (2 mM), **BiPh** (2 mM) and **T** in 50%-50% $CHCl_3$/MeOH.

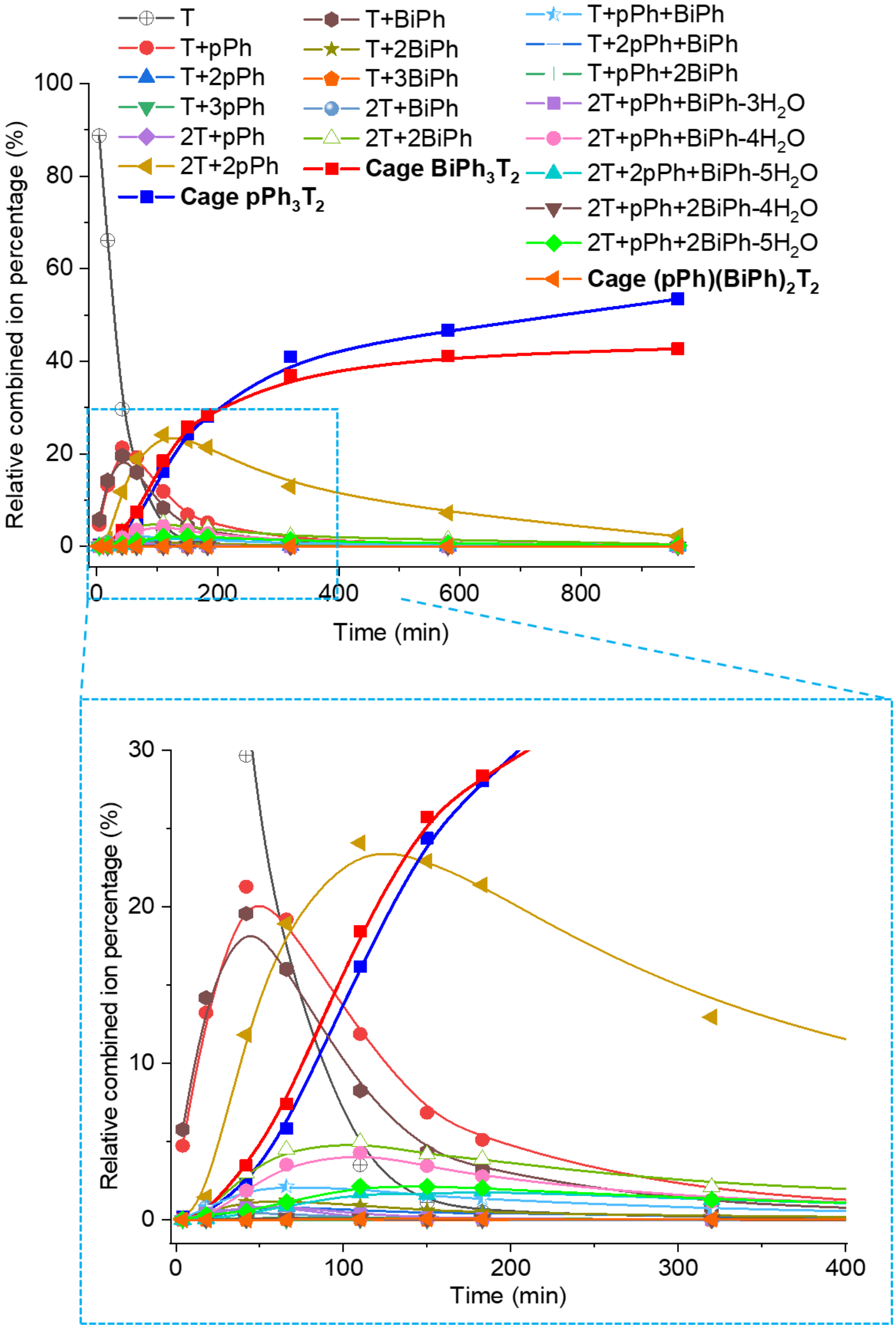

Fig. S50 HRMS-ESI monitoring of the evolution of the species generated during the reaction of 3**pPh** + 3**BiPh** + 4**T** (50%-50% $CHCl_3$/MeOH, r.t) as a function of time over 960 min. NB: The relative combined ion percentage is obtained by the ratio of combined ion count of each species to the sum of combined ion counts for all species at each time point. These data do not provide quantitative information about the relative amounts of each species identified by its mass, but, taken separately, they display the evolution of a given identified species during the course of the reaction. The curves are added to guide the eye.

Table S8. HRMS-ESI intensity comparison of heteroleptic cages **(pPh)(BiPh)$_2$T$_2$** with homoleptic cages

Time (min)	I_1 (a.u.)	I_2 (a.u.)	I_3 (a.u.)	I_3/avg. (I_1+I_2)*100 (%)
4	8.39E+06	1.83E+06	0	0.00
18	1.28E+07	1.44E+07	0	0.00
42	5.22E+07	7.67E+07	0	0.00
66	1.29E+08	1.64E+08	315227.5	0.22
110	3.34E+08	3.80E+08	1.22E+06	0.34
150	5.45E+08	5.76E+08	1.64E+06	0.29
183	6.80E+08	6.88E+08	1.43E+06	0.21
320	1.02E+09	9.16E+08	1.10E+06	0.11
580	1.16E+09	1.02E+09	685708.5	0.06
960	1.27E+09	1.02E+09	175837.5	0.02

I_1: combined ion count of **pPh$_3$T$_2$**

I_2: combined ion count of **BiPh$_3$T$_2$**

I_3: combined ion count of **(pPh)(BiPh)$_2$T$_2$**

7.3 Stepwise self-sorting experiment with BiPh, T and pPh.

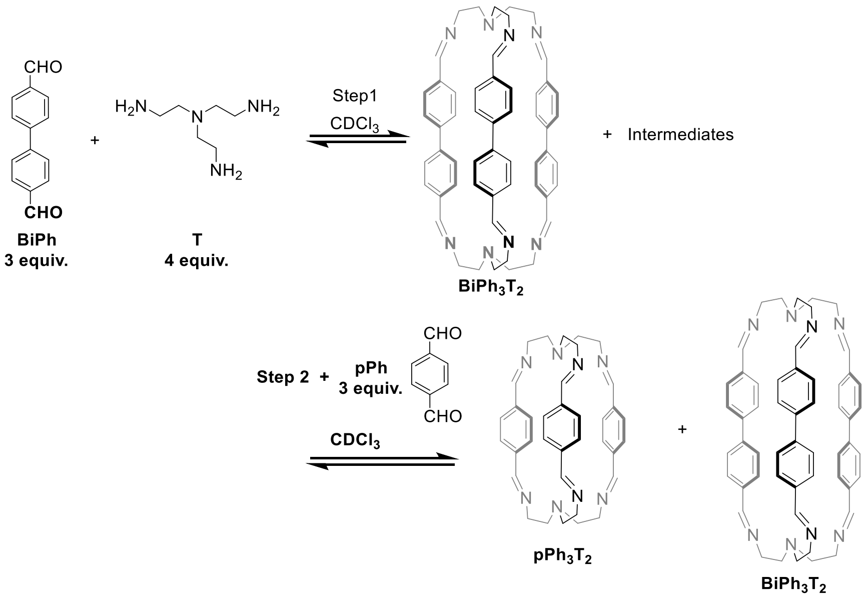

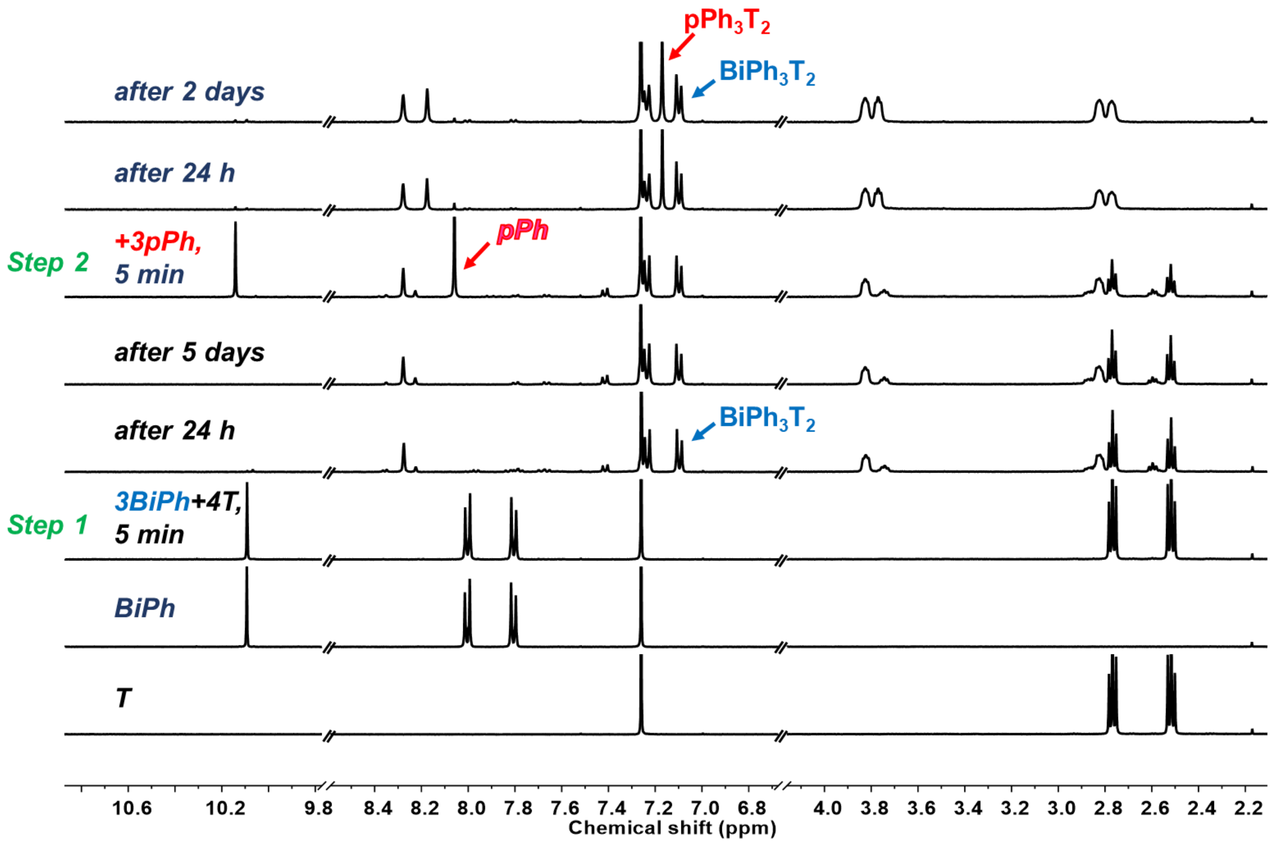

Fig. S51 Temporal evolution of the ^{1}H NMR (400 MHz, $CDCl_3$, 23 °C) spectra of stepwise self-sorting by addition of 3 equiv. **pPh** to the 3**BiPh** + 4**T** solution ([**BiPh**]$_0$ = 3.6 mM) in dated aluminum treated $CDCl_3$.

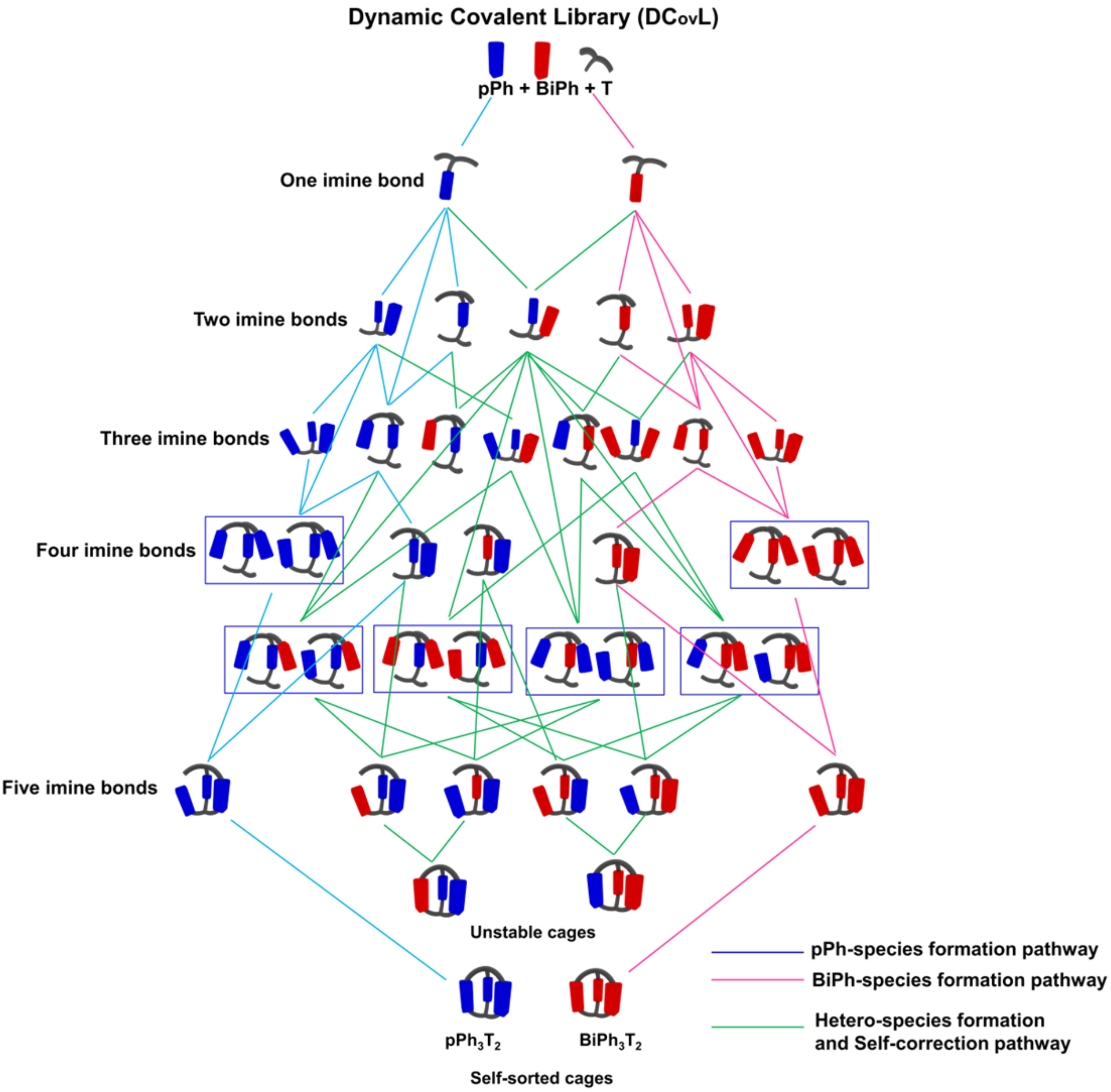

Fig. S52 Possible reaction processes in the three-component [3+2] imine-cage self-sorting system. Blue and red cuboid represent the dialdehyde units, the three-jaw figure represent the triamine T. Cyan and pink lines correspond to the homoleptic reactions to form **[*x*pPh+*y*T]** and **[*x*BiPh+*y*T]** species, respectively. Green lines correspond to the heteroleptic pathways and self-correction pathways.

7.4 DFT calculations

DFT calculations were run with Gaussian 09 (revision B.01).[6] Geometry optimizations were carried out without symmetry restrictions at the B3LYP level,[7] using the 6-311+G(d,p) basis set. The calculations were performed using chloroform as solvent with the Polarizable Continuum Model (PCM) model. Analytical frequency calculations were used to characterise each stationary point as a minimum. These calculations, carried out at 298.15 K, also allowed for obtaining the thermal and entropic corrections required to calculate Gibbs energy differences.

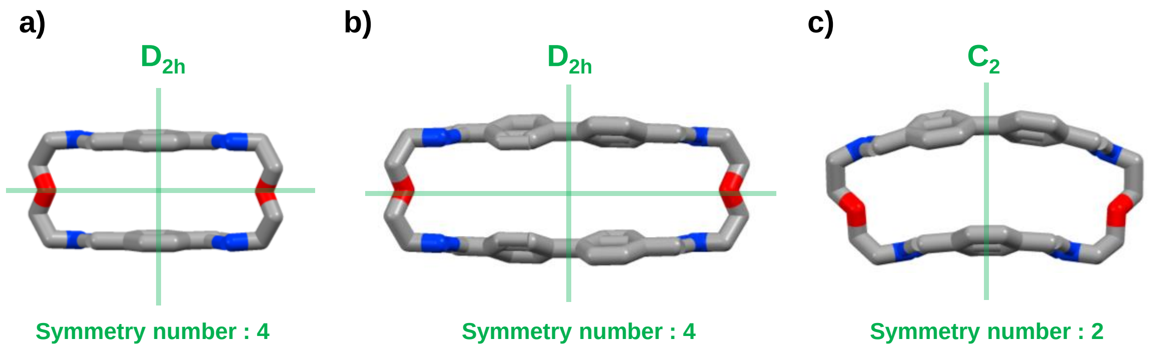

Fig. S53 DFT calculated lowest-in-energy conformations and symmetry numbers for the homoleptic and heteroleptic macrocycles. a) **$pPh_2(NON)_2$**. b) **$BiPh_2(NON)_2$**. c) **$(pPh)(BiPh)(NON)_2$**.

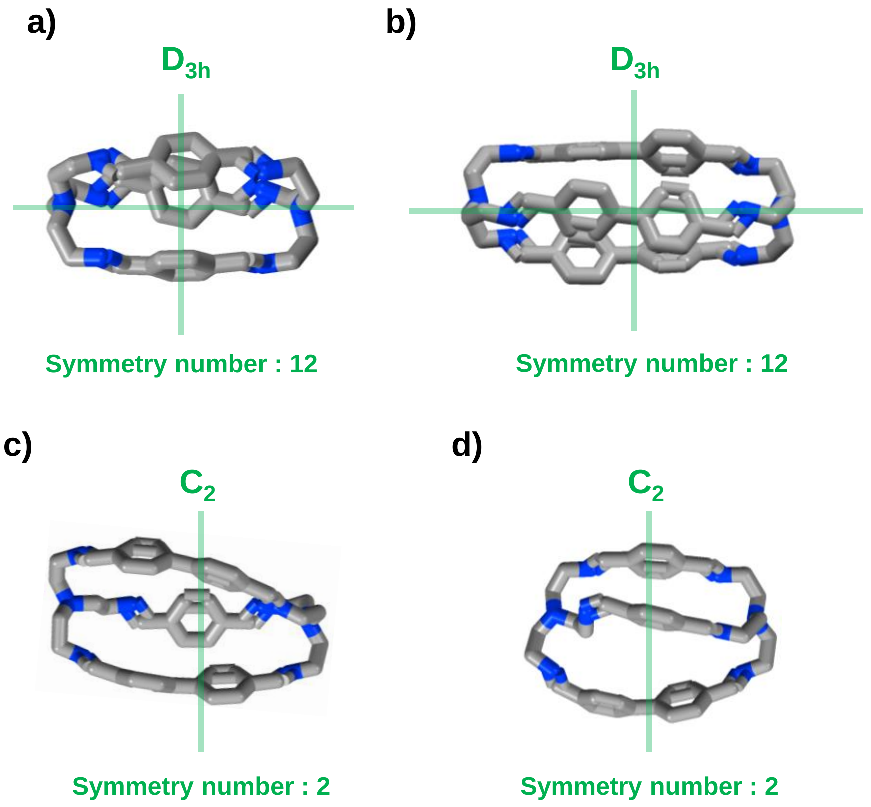

Fig. S54 DFT calculated lowest-in-energy conformations and symmetry numbers for the homoleptic and heteroleptic macrobicyclic cages. a) **pPh_3T_2**. b) **$BiPh_3T_2$**. c) **$(pPh)(BiPh)_2T_2$**. d) **$(pPh)_2(BiPh)T_2$**.

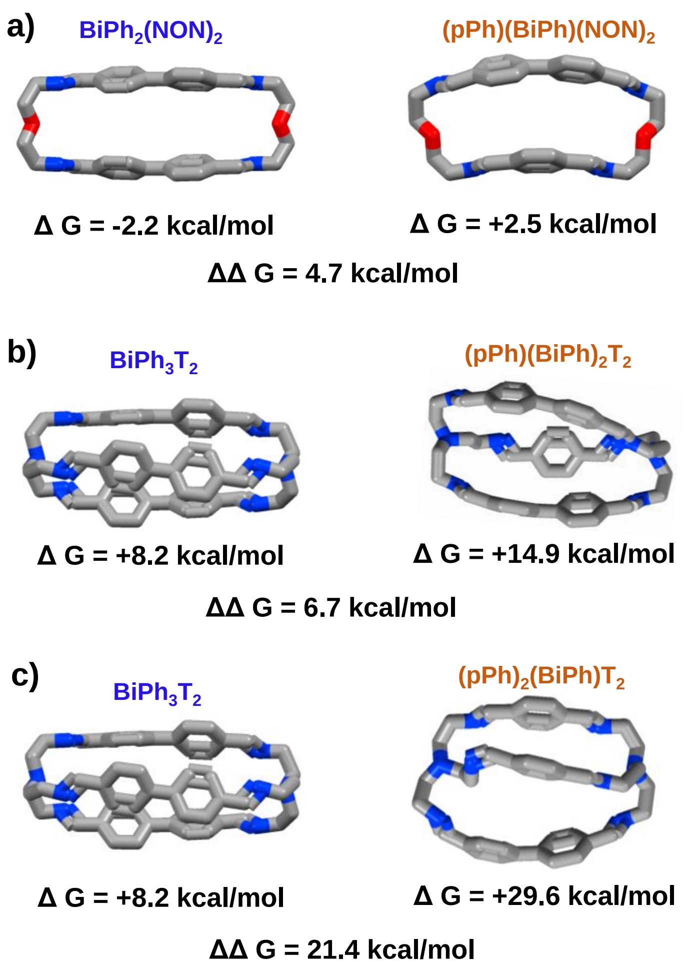

Fig. S55 ΔΔG (heteroleptic – homoleptic) calculated for the **BiPh** derived homoleptic constituents and the possible heteroleptic compounds. a) **$BiPh_2(NON)_2$** and **(pPh)(BiPh)$(NON)_2$**. b) **$BiPh_3T_2$**. and **(pPh)$(BiPh)_2T_2$**. c) **$BiPh_3T_2$**. and **$(pPh)_2(BiPh)T_2$**.

7.5 Self-sorting experiment 2pPh + 2BiPh + 2TriPh + 6NON

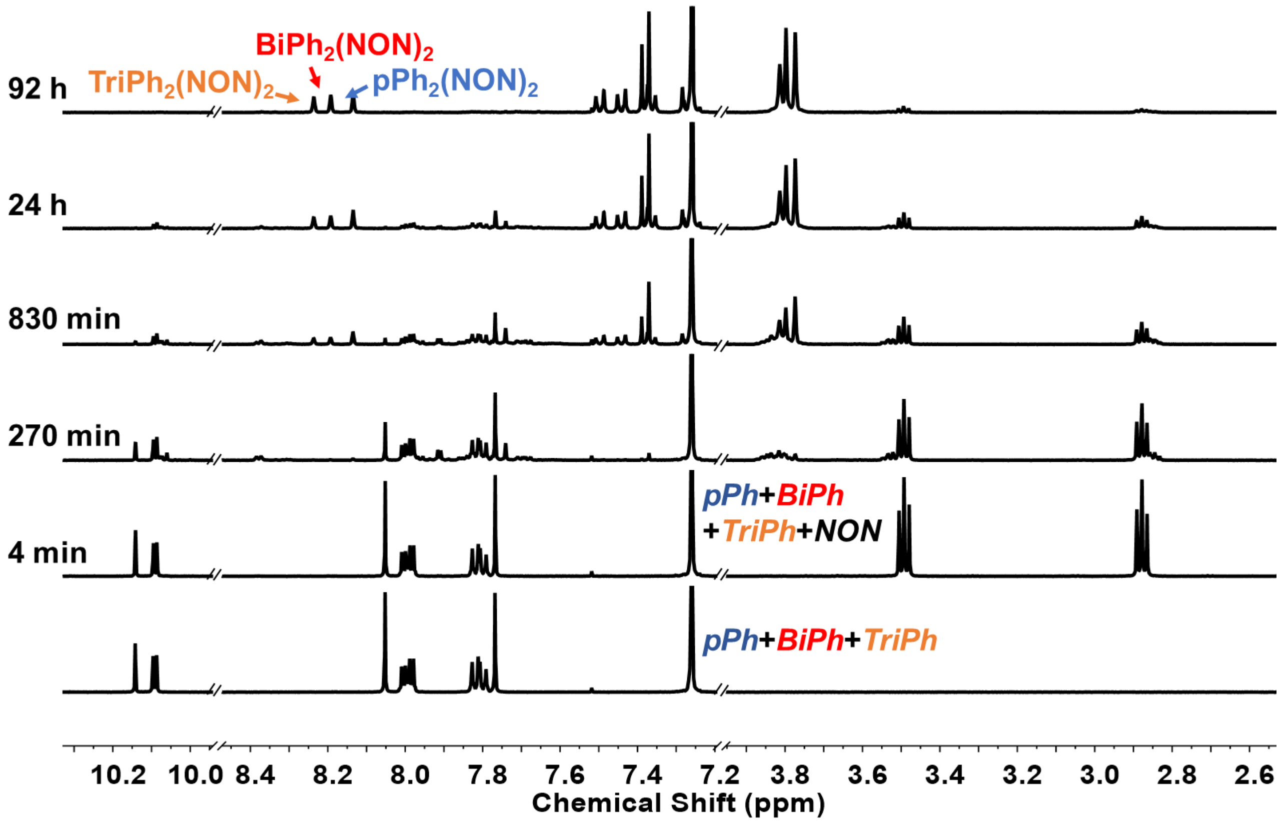

Fig. S56 Temporal evolution of the ^{1}H NMR (400 MHz, $CDCl_3$, 40 °C) spectra of 2**pPh** + 2**BiPh** + 2**TriPh** + 6**NON** ([**pPh**]$_0$ = [**BiPh**]$_0$ = [**TriPh**]$_0$ = 1.0 mM, [**NON**]$_0$ =3.0 mM).

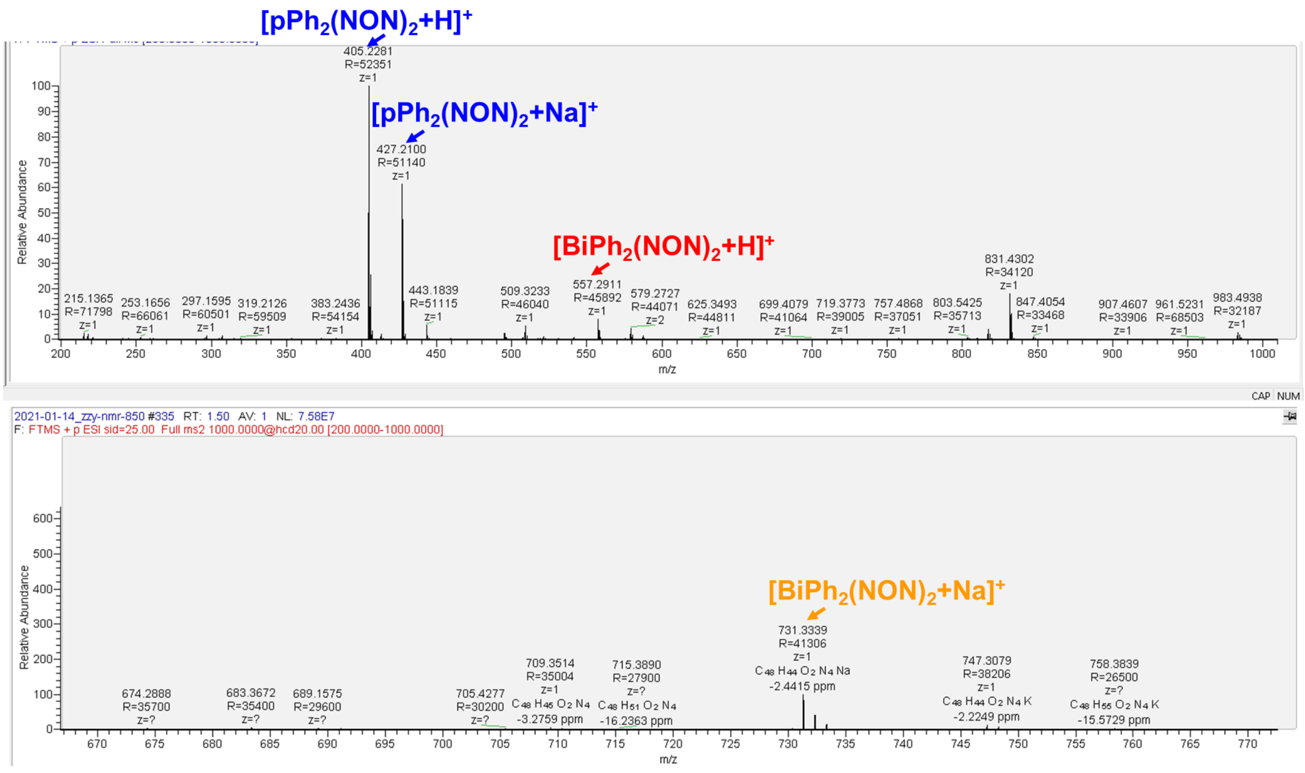

Fig. S57 HRMS-ESI spectrum of three macrocycles obtained in situ from 2**pPh** + 2**BiPh** + 2**TriPh** + 6**NON** reaction in $CDCl_3$ after the equilibration.

7.6 Self-sorting experiment 3pPh + 3BiPh + 3TriPh + 6T

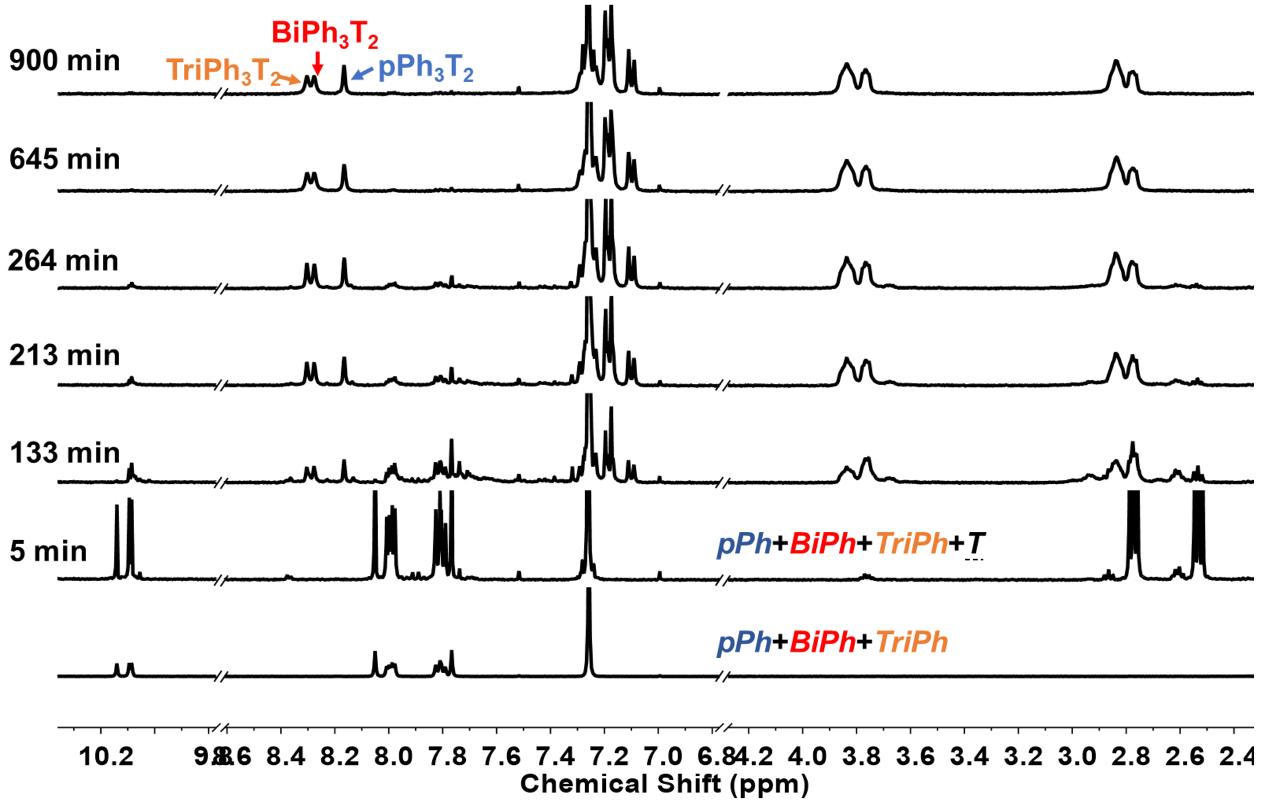

Fig. S58. Temporal evolution of the ^{1}H NMR (400MHz, $CDCl_3$, 40 °C) spectra evolution of 3**pPh** + 3**BiPh** + 3**TriPh** + 6**T** ([**pPh**]$_0$ = [**BiPh**]$_0$ = [**TriPh**]$_0$ = 1.0 mM, [**T**]$_0$ = 2 mM).

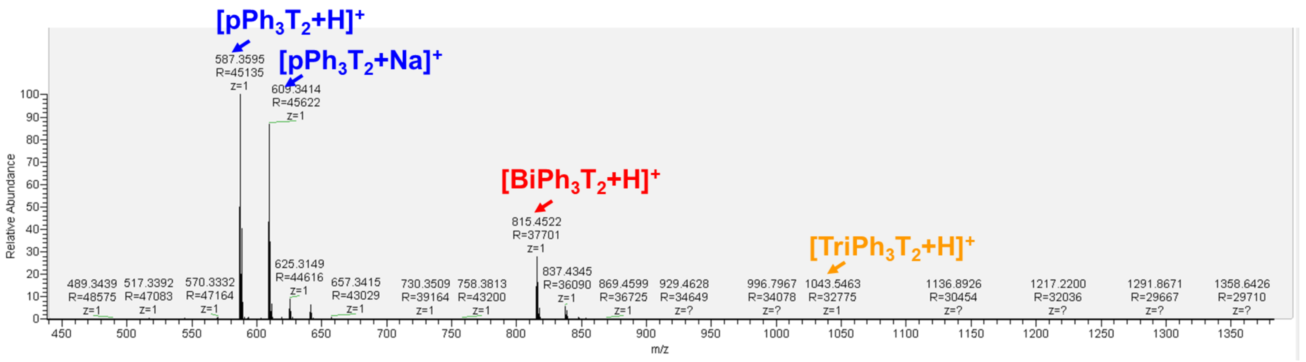

Fig. S59 HRMS-ESI spectrum of three macrobicyclic cages obtained in situ from 3**pPh** + 3**BiPh** + 3**TriPh** + 6**T** reaction in $CDCl_3$ after the equilibration.

7.7 Self-sorting experiment 3Py + 3BiPh + 3TriPh + 2T + 6NON

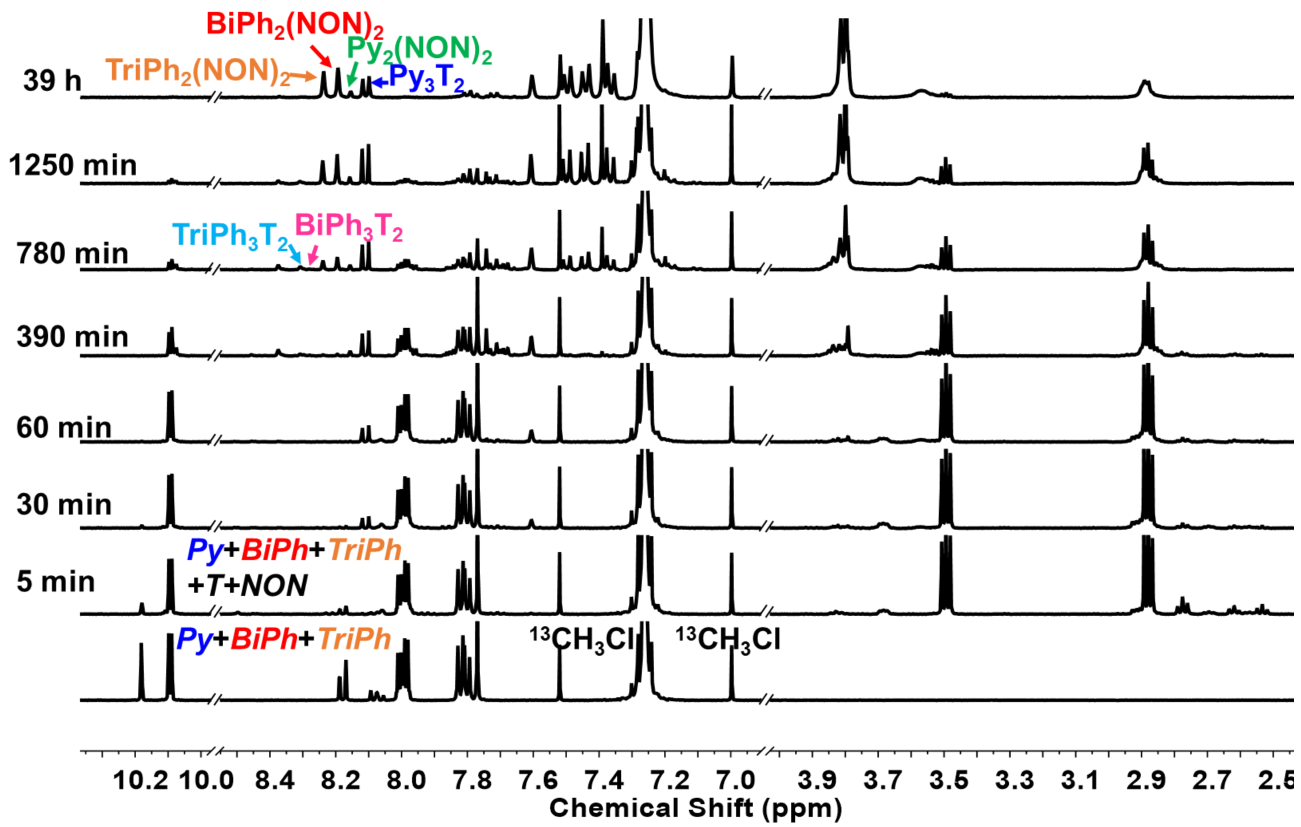

Fig. S60 Temporal evolution of the ^{1}H NMR (400MHz, $CDCl_3$, 40 °C) spectra of 3**Py** + 3**BiPh** + 3**TriPh** + 2**T** + 6**NON** ([**Py**]$_0$ = [**BiPh**]$_0$ = [**TriPh**]$_0$ = 1.0 mM).

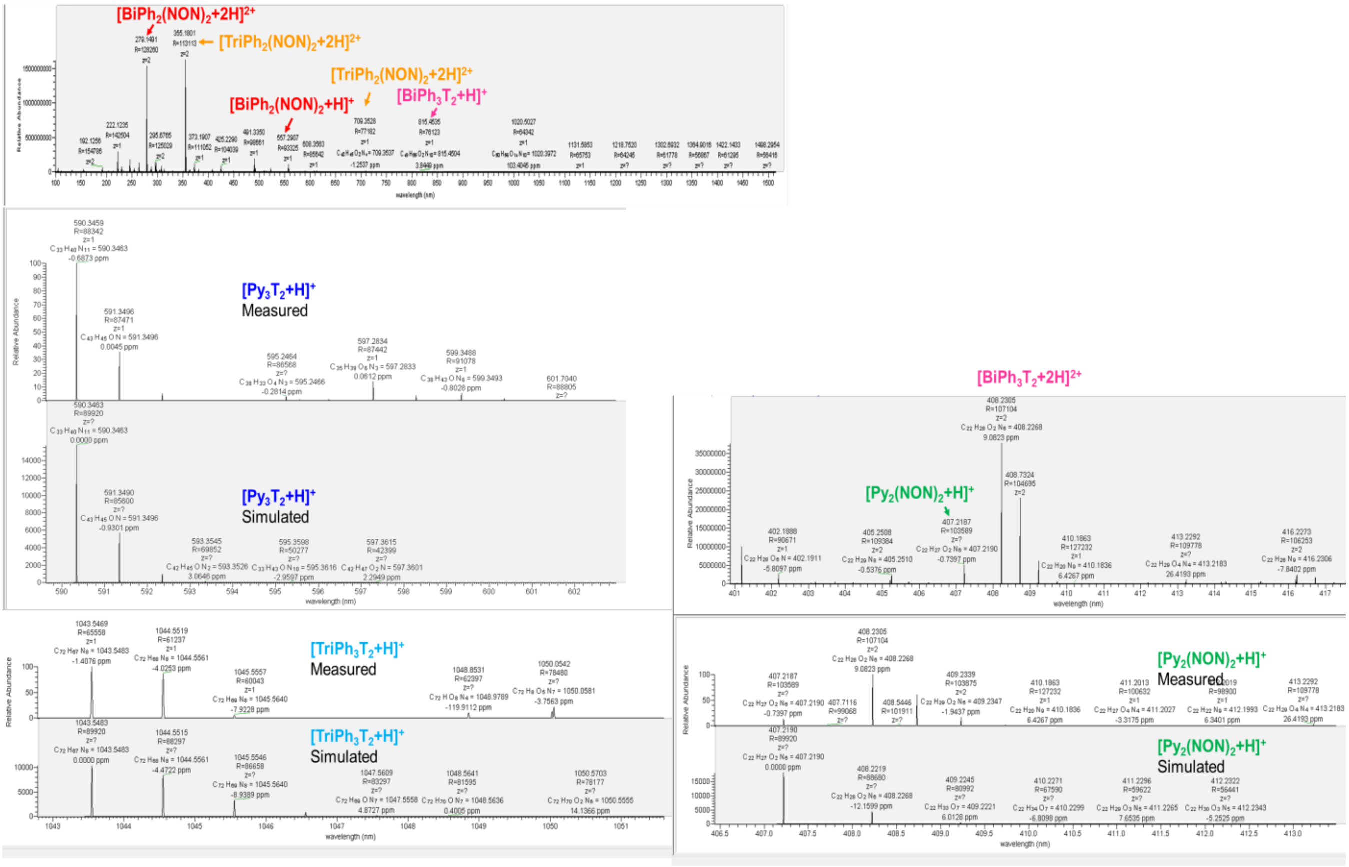

Fig. S61 HRMS-ESI spectrum of the final products obtained in situ from 3**Py** + 3**BiPh** + 3**TriPh** + 2**T** + 6**NON** reaction in $CDCl_3$ after the equilibration.

7.8 Self-sorting experiment 3Py + 3pPh + 3BiPh + 3TriPh + 2T + 9NON

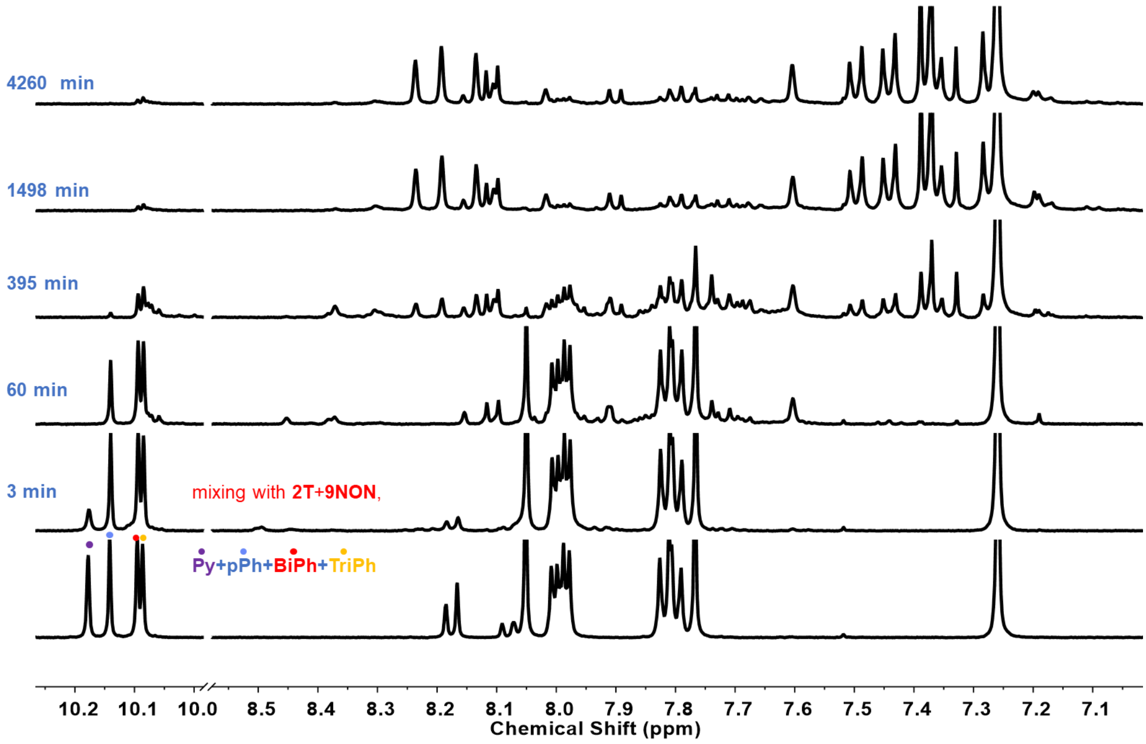

Fig. S62 Temporal evolution of the ^{1}H NMR (400MHz, $CDCl_3$, 40 °C) spectra of 3**Py** + 3**pPh**+ 3**BiPh** + 3**TriPh** + 2**T** + 9**NON** ($[\mathbf{Py}]_0 = [\mathbf{pPh}]_0 = [\mathbf{BiPh}]_0 = [\mathbf{TriPh}]_0 = 2.0$ mM).

8. X-ray crystallography

Single crystal for the macrocycle **pPh$_2$(NON)$_2$** was obtained by slow evaporation of MeCN. Single crystal for **BiPh$_2$(NON)** was obtained by slow vapor diffusion of n-Hexane into a $CDCl_3$ solution of the macrocycle. The single crystal of **TriPh$_3$T$_2$** was obtained from the reaction solution of 3**TriPh**+2**T** at 4 °C in $CHCl_3$. CCDC numbers: 2006765, 2006766 and 2018494, respectively.

Both macrocyclic structures revealed a C_2-symmetry enforced by intramolecular π···π interactions, with the shortest $C_{ar}\cdots C_{ar}$ distance of 3.840 Å and 3.585 Å, respectively. Despite the long distances between the oxygen atoms -$d_{O\cdots O}$= 10.584 and 15.116 Å for **pPh$_2$(NON)$_2$** and **BiPh$_2$(NON)$_2$**- no solvent molecules were observed within the cavity. A water molecule was found to be interacting with the lone pair of the N atoms (imino groups) in **pPh$_2$(NON)$_2$**, with a $N\cdots O_{water}$ distance of 2.896 Å. The **TriPh$_3$T$_2$**·molecular structure showed a distorted D_3 symmetry and the aromatic bridging units were also interacting through π···π forces (shortest C···C distance = 3.425 Å). These intramolecular interactions were likely compressing the 3D-conformation of the cyclic species and thus reducing the cavity void, hampering the encapsulation of guest molecules.

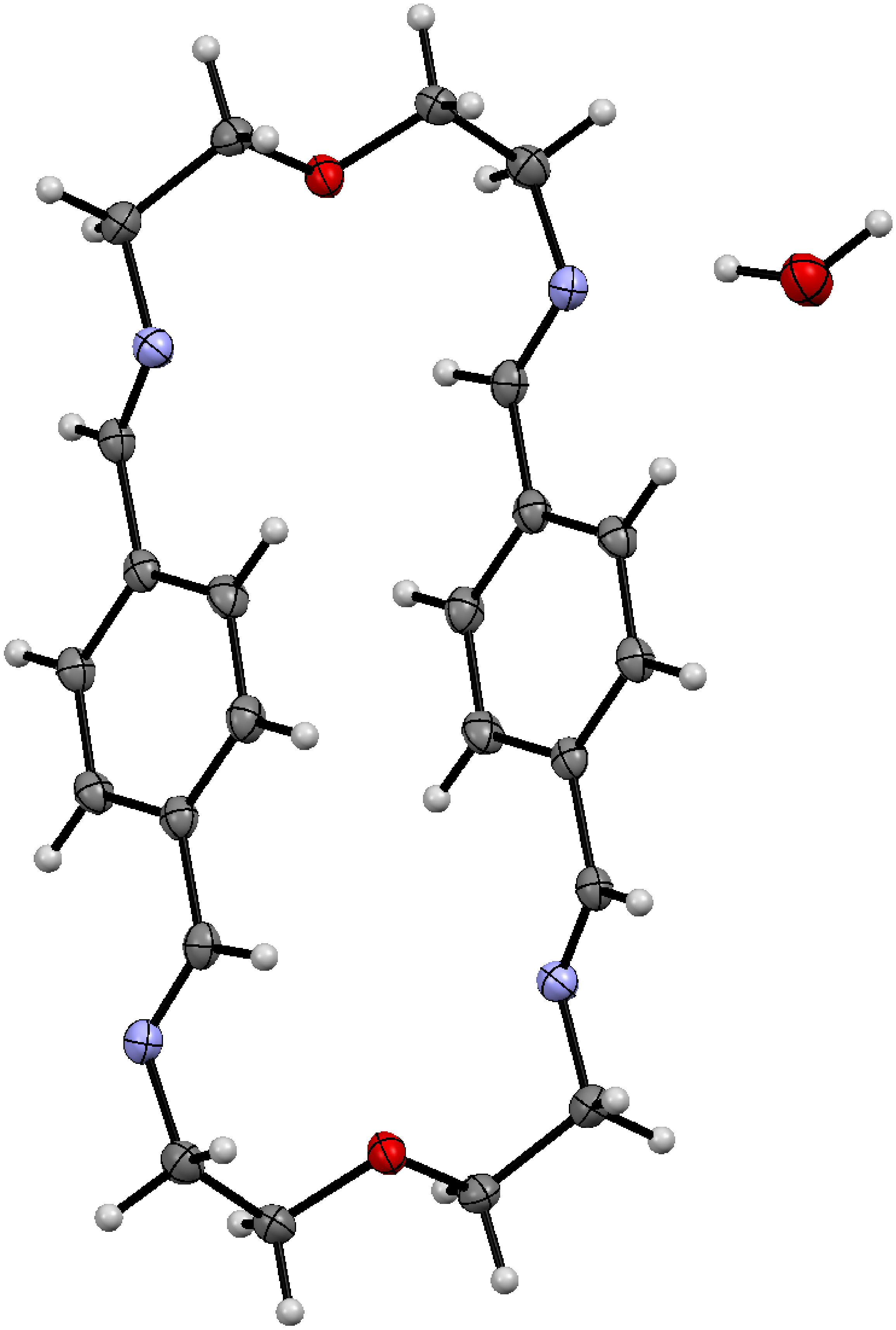

Fig. S63 Thermal ellipsoid plot for Macrocycle **pPh$_2$(NON)$_2$·H$_2$O**. Ellipsoids at 50% probability. Color scheme: carbon (grey), hydrogen (white), nitrogen (blue), oxygen (red).

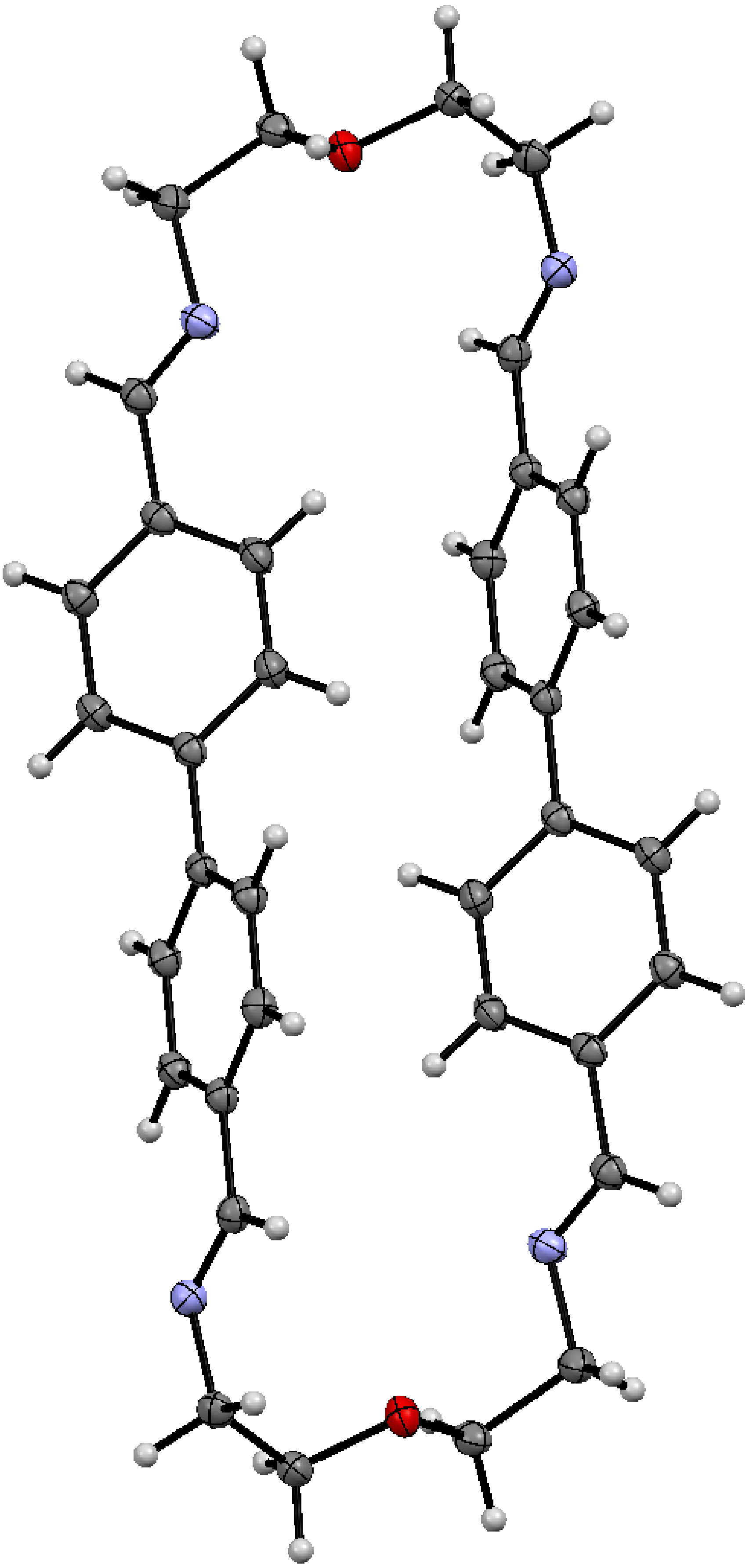

Fig. S64 Thermal ellipsoid plot for Macrocycle **$BiPh_2(NON)_2$**. Ellipsoids at 50% probability. Color scheme: carbon (grey), hydrogen (white), nitrogen (blue), oxygen (red).

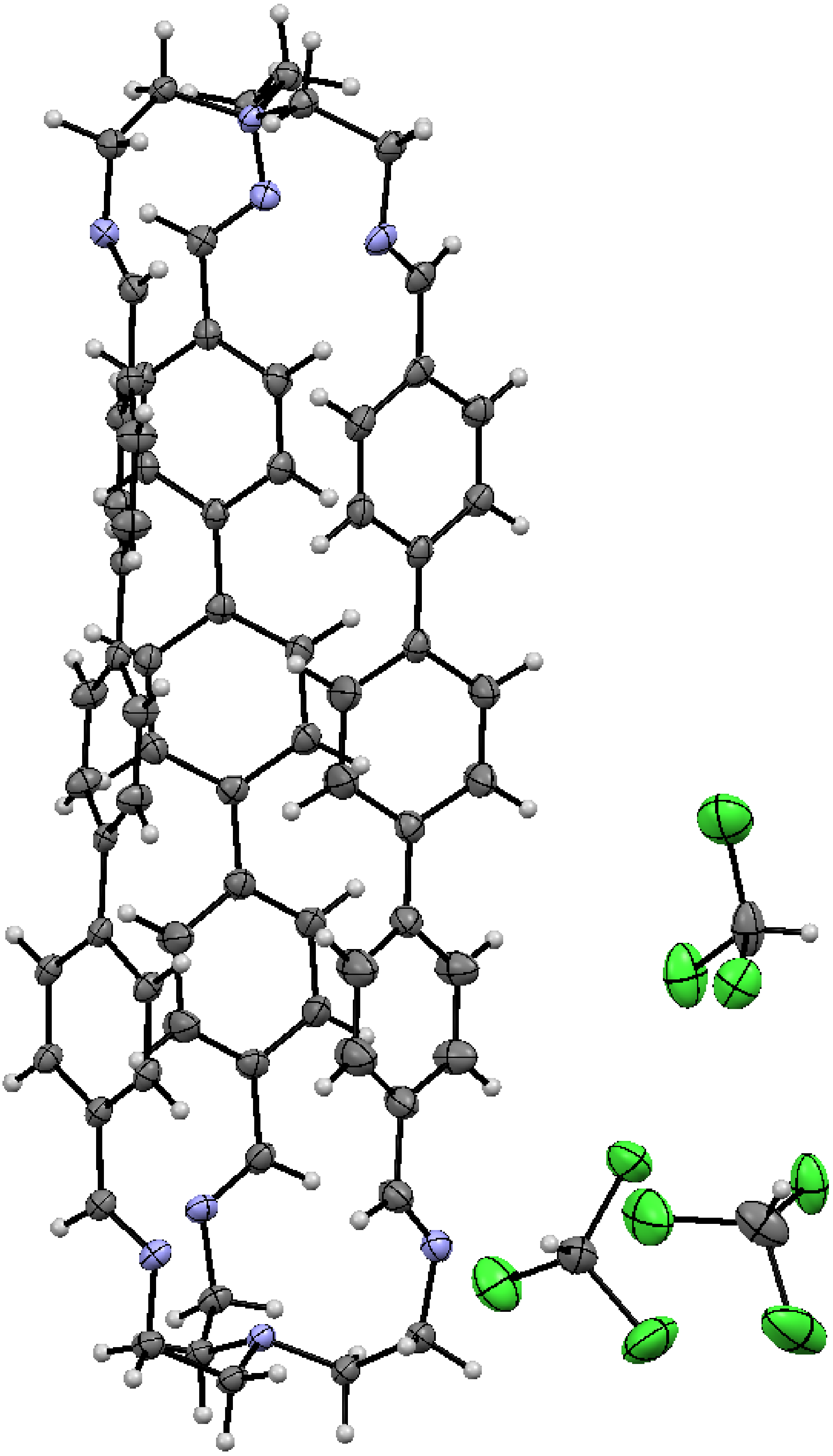

Fig. S65 Thermal ellipsoid plot for Macrobicyclic cage **TriPh$_3$T$_2$·3CHCl$_3$**. Ellipsoids at 50% probability. Color scheme: carbon (grey), hydrogen (white), nitrogen (blue), chlorine (green).

Table S9 Crystal data and structure refinement for **pPh$_2$(NON)$_2$**, **BiPh$_2$(NON)$_2$** and **TriPh$_3$T$_2$**

Identification code	**pPh$_2$(NON)$_2$·H$_2$O**	**BiPh$_2$(NON)$_2$**	**TriPh$_3$T$_2$·3CHCl$_3$**
Empirical formula	$C_{24}H_{28}N_4O_2 \cdot H_2O$	$C_{36}H_{36}N_4O_2$	$C_{72}H_{66}N_8$, 3($CHCl_3$)
Formula weight	422.52	556.69	1401.43
Temperature/K	120(2)	120(2)	120(2)
Crystal system	orthorhombic	triclinic	triclinic
Space group	P c c n	P− 1	P -1
a/Å	16.0157(5)	8.9175(4)	12.9054(10)
b/Å	16.9055(6)	9.6423(5)	13.4545(11)
c/Å	8.2684(2)	10.4108(5)	22.5621(18)
α/°	90	62.612(2)	76.114(3)
β/°	90	67.462(2)	82.526(3)
γ/°	90	72.983(2)	62.714(3)
Volume/Å^3	2238.70(12)	726.71(6)	3379.0(5)
Z	4	1	2
ρ_{calcd}/g·cm^{-3}	1.254	1.272	1.377
μ/mm^{-1}	0.084	0.080	0.424
F(000)	904	296	1456
Crystal size/mm^3	0.120×0.140×0.160	0.200×0.150×0.080	0.400×0.200×0.140
Radiation	0.71073	0.71073	0.71073
2θ range for data collection/°	2.410-28.012	2.304-30.008	1.986-27.978
Index ranges	$-21 \le h \le 21, -22 \le k \le 21, -10 \le l \le 10$	$-11 \le h \le 12, -13 \le k \le 13, -14 \le l \le 14$	$-16 \le h \le 17, -17 \le k \le 17, -29 \le l \le 29$
Reflections collected	27088	27416	147869
Reflns. unique	2703	4246	16195
Reflns. obsd.	2311	3538	12684
R_{int}	0.0412	0.0341	0.0546
Params. refined	145	190	829
Goodness-of-fit on F^2	1.054	1.025	1.031
Final R indexes [I>=2σ (I)]	R_1=0.0432, wR_2= 0. 1117	R_1= 0.0394, wR_2= 0.1026	R_1= 0.0659, wR_2= 0.1778
Final R indexes [all data]	R_1= 0.0513, wR_2= 0.1168	R_1= 0.0498, wR_2= 0.1104	R_1= 0.0831, wR_2= 0.1941
Largest diff. peak/hole / e Å^{-3}	0.350/-0.495	0.380/-0.189	1.932/-1.456

9. References

1. G. M. Sheldrick, *Acta Crystallogr., Sect. A: Found. Crystallogr.*, 2008, **64**, 112–122.
2. C. F.Macrae, I. J.Bruno, J. A. Chisholm, P. R. Edgington, P. McCabe, E. Pidcock, L. Rodriguez-Monge, R. Taylor, J. van de Streek, and P. A. Wood, *J. Appl. Crystallogr.*, 2008, **41**, 466–470.
3. N. Alzakhem, C. Bischof, M. Seitz, *Inorg. Chem.* **2012**, *51*, 9343–9349.
4. M. Kołodziejski, A. R. Stefankiewicz, J.-M. Lehn, *Chem. Sci.* **2019**, *10*, 1836–1843.
5. Z. Yang, J.-M. Lehn, *J. Am. Chem. Soc.* **2020**, *142*, 15137–15145.
6. M. J. Frisch, G. W. Trucks, H. B. Schlegel, G. E. Scuseria, M. A. Robb, J. R. Cheeseman, G. Scalmani, V. Barone, B. Mennucci, G. A. Petersson, et al., Gaussian 09, Revision B.01 (Gaussian, Inc), **2010**.
7. A. D. Becke, *J. Chem. Phys.* **1993**, *98*, 5648-5652.

10. Cartesian coordinates and energies

- Lowest energy conformation for **H_2O**.

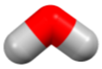

Cartesian coordinates (3 atoms), E (298.15 K) = -76.44962 Hartree				
1	O	-5.0653	2.3918	0.1955
2	H	-4.1039	2.4425	0.1955
3	H	-5.3385	3.315	0.1955

- Lowest energy conformation for **NON**.

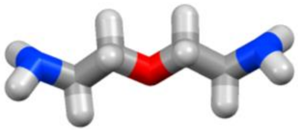

Cartesian coordinates (19 atoms), E (298.15 K) = -344.317532 Hartree				
1	C	-1.6371	1.0687	0.0257
2	H	-1.2072	0.062	0.0379
3	H	-1.2046	1.5847	-0.8376
4	C	-1.201	1.7962	1.2928
5	H	-1.6056	2.8194	1.2909
6	H	-1.6092	1.2817	2.1756
7	O	0.2171	1.8189	1.3376
8	C	0.7381	2.481	2.4793
9	H	0.3931	1.9924	3.4028
10	H	0.3962	3.5263	2.5116
11	C	2.2609	2.4403	2.4142
12	H	2.5813	2.9126	1.4799
13	H	2.5789	1.3941	2.3626
14	N	-3.1078	1.0252	-0.0541
15	H	-3.4333	1.4754	-0.9008
16	H	-3.4352	0.0678	-0.0931
17	N	2.8369	3.1216	3.587
18	H	3.426	2.489	4.1143
19	H	3.4276	3.8917	3.2982

- Lowest energy conformation for **T**.

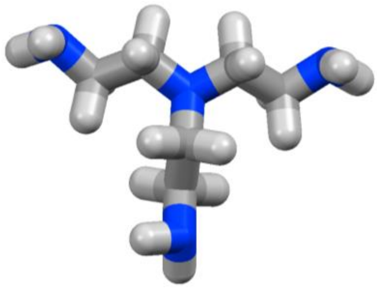

Cartesian coordinates (28 atoms), E (298.15 K) = -458.367788 Hartree				
1	C	-1.7944	0.7755	0.2214
2	H	-1.3583	-0.1752	0.5415
3	H	-1.4724	0.9438	-0.8168
4	C	-1.2514	1.883	1.1262

5	H	-1.7489	2.8372	0.864
6	H	-1.5476	1.6446	2.1499
7	C	0.7569	2.6731	2.2574
8	H	0.203	2.3404	3.1377
9	H	0.6316	3.7723	2.1984
10	C	2.2337	2.3544	2.4962
11	H	2.826	2.6249	1.6091
12	H	2.3348	1.2735	2.6293
13	N	-3.2559	0.6914	0.3691
14	H	-3.6868	1.5073	-0.0582
15	H	-3.6044	-0.1078	-0.1516
16	N	2.6909	3.0211	3.7252
17	H	3.6404	2.7301	3.9373
18	H	2.7379	4.0249	3.5699
19	N	0.2067	2.003	1.0787
20	C	0.7035	2.5511	-0.1932
21	H	-0.1328	2.8669	-0.8279
22	H	1.2954	3.4574	-0.0026
23	C	1.5577	1.5601	-0.9901
24	H	2.3965	1.2249	-0.3621
25	H	0.9555	0.6742	-1.2102
26	N	1.9707	2.1644	-2.2654
27	H	2.6505	2.9	-2.0898
28	H	2.4534	1.4731	-2.8313

- Lowest energy conformation for **pPh**.

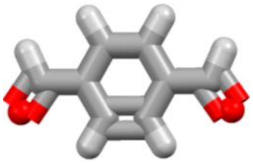

Cartesian coordinates (16 atoms), E (298.15 K) = -458.936582 Hartree

1	C	-3.314	2.2748	0.0002
2	C	-1.9231	2.2747	0.0006
3	C	-1.2268	3.4867	0.0001
4	C	-1.9269	4.704	-0.001
5	C	-3.3099	4.704	-0.0015
6	C	-4.0102	3.4869	-0.0009
7	H	-3.8624	1.3389	0.0006
8	H	-1.3748	1.3388	0.0014
9	H	-1.3645	5.6297	-0.0014
10	H	-3.8722	5.6299	-0.0023
11	C	-5.4932	3.4749	-0.0012
12	H	-5.9477	2.4638	-0.0007
13	C	0.2562	3.4746	0.0007
14	H	0.7106	2.4634	0.0015
15	O	-6.1869	4.4681	-0.0019
16	O	0.95	4.4677	0.0004

- Lowest energy conformation for **BiPh**.

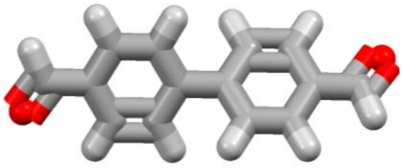

Cartesian coordinates (26 atoms), E (298.15 K) = -689.976039 Hartree

1	C	-2.6353	0.6156	0.0278
2	H	-3.6928	0.538	-0.2979
3	O	-2.1622	1.6877	0.3428
4	C	-1.8999	-0.6654	0.0359
5	C	-2.5497	-1.8434	-0.3464
6	C	-0.5542	-0.7175	0.4228
7	C	-1.8696	-3.0544	-0.343
8	H	-3.5898	-1.8078	-0.6541
9	C	0.1247	-1.9245	0.4227
10	H	-0.0613	0.1983	0.7264
11	C	-0.5205	-3.1157	0.0399
12	H	-2.3796	-3.9548	-0.6631
13	H	1.1584	-1.9555	0.7454
14	C	0.2099	-4.4061	0.0402
15	C	1.5586	-4.468	-0.344
16	C	-0.435	-5.597	0.4249
17	C	2.2385	-5.6791	-0.347
18	H	2.0684	-3.568	-0.6655
19	C	0.2436	-6.804	0.4255
20	H	-1.4684	-5.5654	0.7485
21	C	1.589	-6.8566	0.0371
22	H	3.2782	-5.7152	-0.6559
23	H	-0.249	-7.7194	0.7307
24	C	2.3242	-8.1377	0.0296
25	H	3.3812	-8.0607	-0.2977
26	O	1.8514	-9.2094	0.3467

- Lowest energy conformation for **(pPh)$_2$NON$_2$**.

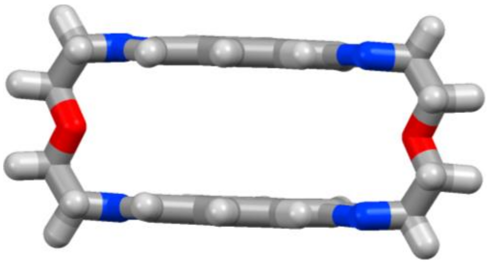

Cartesian coordinates (58 atoms), E (298.15 K) = -1300.716382 Hartree

1	C	10.0912	7.8671	5.9351
2	C	10.141	9.2564	5.7445
3	H	9.9422	9.9162	6.5828
4	C	10.431	9.7935	4.4991
5	H	10.4634	10.8651	4.3479

6	C	10.6738	8.9501	3.4065
7	C	10.629	7.5607	3.5983
8	H	10.8125	6.901	2.7563
9	C	10.3448	7.0237	4.8449
10	H	10.3031	5.952	4.9942
11	C	10.955	9.4855	2.0636
12	H	11.0678	8.7317	1.2705
13	C	11.3116	11.1374	0.4491
14	H	11.4099	10.2817	-0.2359
15	H	12.2511	11.7015	0.4265
16	C	10.2096	12.059	-0.055
17	H	10.0633	12.8833	0.6563
18	H	10.5064	12.489	-1.0234
19	C	8.1163	4.802	8.8398
20	H	8.261	3.9826	8.1226
21	H	7.9534	4.363	9.8355
22	C	9.3594	5.681	8.8712
23	H	9.1789	6.5375	9.5382
24	H	10.1764	5.0854	9.2941
25	C	9.7559	7.3319	7.2656
26	H	9.512	8.0858	8.0285
27	N	11.0601	10.7274	1.8198
28	N	9.7404	6.0899	7.5303
29	O	9.013	11.3094	-0.193
30	C	5.9242	9.0375	2.3328
31	C	5.8734	7.6483	2.5242
32	H	6.0714	6.9879	1.6861
33	C	5.5834	7.1122	3.7699
34	H	5.5502	6.0406	3.9217
35	C	5.3417	7.9563	4.8622
36	C	5.3875	9.3456	4.6696
37	H	5.2048	10.0059	5.5113
38	C	5.6716	9.8817	3.4226
39	H	5.7141	10.9532	3.2727
40	C	5.0608	7.4218	6.2055
41	H	4.949	8.1762	6.9983
42	C	4.7042	5.771	7.8212
43	H	4.6076	6.627	8.506
44	H	3.764	5.2081	7.8449
45	C	5.8055	4.8481	8.3246
46	H	5.9501	4.0235	7.6133
47	H	5.509	4.4186	9.2933
48	C	7.8993	12.1021	-0.5727
49	H	7.7532	12.9219	0.1438
50	H	8.0626	12.5406	-1.5685
51	C	6.6572	11.2218	-0.6043
52	H	6.839	10.3649	-1.2705

53	H	5.8398	11.8162	-1.0283
54	C	6.2597	9.5718	1.0019
55	H	6.5034	8.8173	0.2395
56	N	4.9551	6.1801	6.4501
57	N	6.2757	10.8136	0.7366
58	O	7.0032	5.5962	8.4615

- Lowest energy conformation for **$(BiPh)_2NON_2$**.

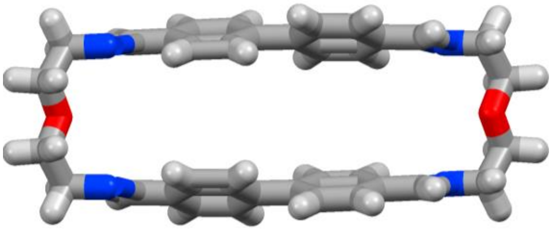

Cartesian coordinates (78 atoms), E (298.15 K) = -1762.792149 Hartree

1	C	9.1691	4.2199	3.4964
2	C	9.2804	4.903	2.2703
3	H	8.3886	5.1139	1.6923
4	C	10.5059	5.3501	1.8043
5	H	10.5776	5.8876	0.867
6	C	11.6736	5.1414	2.5522
7	C	11.5718	4.463	3.7722
8	H	12.465	4.2876	4.3632
9	C	10.3437	4.0029	4.2326
10	H	10.3007	3.457	5.1674
11	C	12.9846	5.6309	2.0959
12	H	13.8009	5.5414	2.8282
13	C	14.5118	6.5978	0.6126
14	H	14.9147	5.9599	-0.1834
15	H	15.2008	6.5469	1.4692
16	C	14.4685	8.0201	0.0747
17	H	15.4603	8.2968	-0.3136
18	H	13.7491	8.0741	-0.7539
19	C	13.8887	10.2333	0.6644
20	H	13.1723	10.2503	-0.1684
21	H	14.8392	10.6583	0.3077
22	C	13.3516	11.08	1.8079
23	H	14.0438	11.0316	2.6621
24	H	13.3218	12.1189	1.4571
25	C	11.7387	10.471	3.3883
26	H	12.5044	10.5894	4.1696
27	C	10.4093	10.0579	3.8668
28	C	10.1489	10.0124	5.2411
29	H	10.9265	10.2963	5.943
30	C	8.9042	9.6195	5.7188
31	H	8.7236	9.6216	6.787
32	C	7.8506	3.7657	4.0003
33	C	8.1502	9.2884	3.4576

34	H	7.3908	8.9727	2.7524
35	C	9.3862	9.6926	2.9801
36	H	9.5878	9.7117	1.9164
37	N	13.1779	6.1258	0.9424
38	N	11.9996	10.6837	2.1636
39	O	14.0808	8.9022	1.1163
40	C	6.5607	8.7872	5.342
41	C	6.4494	8.104	6.568
42	H	7.3411	7.893	7.146
43	C	5.2238	7.657	7.0341
44	H	5.1521	7.1194	7.9713
45	C	4.0562	7.8659	6.2862
46	C	4.158	8.5443	5.0663
47	H	3.2648	8.7198	4.4753
48	C	5.3861	9.0043	4.6058
49	H	5.4291	9.5503	3.6712
50	C	2.7452	7.3765	6.7425
51	H	1.9288	7.466	6.0102
52	C	1.218	6.4097	8.2259
53	H	0.815	7.0476	9.0219
54	H	0.5288	6.4606	7.3693
55	C	1.2612	4.9874	8.7638
56	H	0.2694	4.7107	9.1522
57	H	1.9806	4.9334	9.5924
58	C	1.841	2.7742	8.1742
59	H	2.5574	2.7573	9.007
60	H	0.8906	2.3491	8.5309
61	C	2.3782	1.9274	7.0307
62	H	1.686	1.9757	6.1765
63	H	2.4081	0.8886	7.3816
64	C	3.9912	2.5361	5.4503
65	H	3.2256	2.4174	4.669
66	C	5.3206	2.9492	4.9718
67	C	5.581	2.9946	3.5974
68	H	4.8033	2.7107	2.8956
69	C	6.8257	3.3875	3.1197
70	H	7.0062	3.3854	2.0515
71	C	7.8792	9.2414	4.8382
72	C	7.5797	3.7188	5.3809
73	H	8.3391	4.0345	6.0861
74	C	6.3437	3.3145	5.8584
75	H	6.1421	3.2955	6.9222
76	N	2.5518	6.8816	7.8961
77	N	3.7302	2.3239	6.675
78	O	1.6487	4.1052	7.7222

- Lowest energy conformation for **(pPh)(BiPh)NON$_2$**.

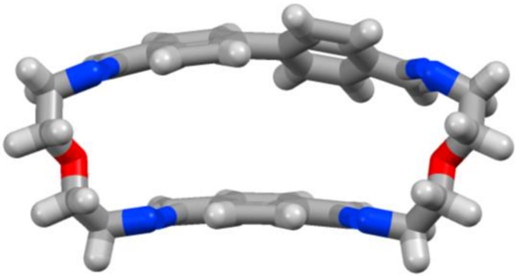

Cartesian coordinates (68 atoms), E (298.15 K) = -1531.745179 Hartree

1	C	-1.5071	3.4309	-1.3185
2	C	-2.8845	3.2619	-1.2319
3	C	-3.4876	2.91	-0.0188
4	C	-2.6822	2.7953	1.1227
5	C	-1.3112	2.9576	1.0348
6	C	-0.6885	3.2413	-0.1951
7	C	-4.9117	2.5461	0.0234
8	C	0.7885	3.2012	-0.2952
9	N	-5.4964	2.2012	1.0967
10	O	-6.0508	-0.5517	0.6242
11	N	-3.6664	-2.4899	0.3596
12	C	-6.8446	1.6763	1.0048
13	C	-6.8608	0.2301	1.4888
14	C	-5.9987	-1.9184	1.0049
15	C	-5.0788	-2.6769	0.0591
16	N	3.5381	-2.5223	0.7234
17	O	5.984	-0.6201	0.782
18	N	5.6684	2.2486	0.7454
19	C	4.9615	-2.7844	0.5664
20	C	5.8438	-1.8818	1.4188
21	C	6.8623	0.2561	1.4727
22	C	6.9505	1.5697	0.7002
23	C	4.9594	2.2361	-0.3087
24	C	2.8583	-2.2809	-0.3228
25	C	2.7782	2.4415	-1.461
26	C	1.4091	2.678	-1.4388
27	C	1.6059	3.5566	0.7946
28	C	2.9675	3.308	0.7787
29	C	3.5684	2.709	-0.3381
30	C	-0.6516	-1.8793	-1.6112
31	C	0.7378	-1.9075	-1.5461
32	C	1.3903	-2.1418	-0.3305
33	C	0.6147	-2.3157	0.8302
34	C	-0.768	-2.2948	0.7642
35	C	-1.4238	-2.0932	-0.4637
36	C	-2.89	-2.1783	-0.5968
37	H	-1.0629	3.6914	-2.272
38	H	-3.496	3.3753	-2.1215

39	H	-3.1481	2.5193	2.06
40	H	-0.7038	2.7919	1.9161
41	H	-5.4283	2.5289	-0.9476
42	H	-7.2491	1.7249	-0.0177
43	H	-7.502	2.2552	1.6631
44	H	-7.8952	-0.1452	1.4906
45	H	-6.4755	0.1809	2.5164
46	H	-5.6313	-2.0177	2.0354
47	H	-7.0062	-2.358	0.9573
48	H	-5.2931	-3.7448	0.188
49	H	-5.3296	-2.4079	-0.978
50	H	5.1328	-3.8147	0.8995
51	H	5.2912	-2.7108	-0.4809
52	H	5.4046	-1.7645	2.4189
53	H	6.8316	-2.3525	1.5319
54	H	6.4914	0.4476	2.4888
55	H	7.8643	-0.1914	1.5494
56	H	7.7069	2.1966	1.183
57	H	7.2801	1.3558	-0.3277
58	H	5.3301	1.7998	-1.2475
59	H	3.3353	-2.2116	-1.3118
60	H	3.2299	1.9867	-2.3366
61	H	0.808	2.3842	-2.2905
62	H	1.1605	4.0085	1.673
63	H	3.5809	3.5358	1.6415
64	H	-1.1434	-1.715	-2.5643
65	H	1.3231	-1.7677	-2.4489
66	H	1.1215	-2.4918	1.771
67	H	-1.3665	-2.4563	1.6523
68	H	-3.2746	-2.001	-1.6122

- Lowest energy conformation for **pPh$_3$T$_2$**.

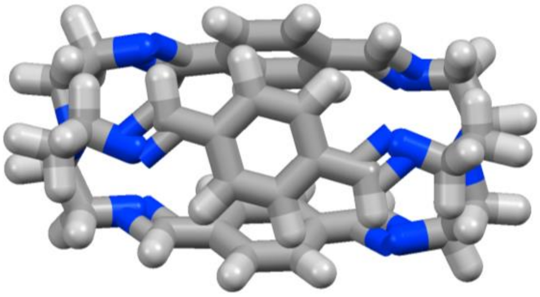

Cartesian coordinates (86 atoms), E (298.15 K) = -1834.844653 Hartree

1	C	4.6022	10.184	-1.6052
2	H	4.9772	11.0786	-1.1067
3	H	4.6412	10.3849	-2.693
4	C	5.5888	9.0519	-1.2936
5	H	5.2697	8.1022	-1.747
6	H	6.5371	9.33	-1.7703
7	C	5.7349	7.7616	0.6483

8	H	5.456	6.8857	0.0443
9	C	5.9488	7.493	2.0806
10	C	5.7589	6.1931	2.5735
11	H	5.5081	5.3951	1.8825
12	C	5.8548	5.9249	3.9309
13	H	5.6811	4.928	4.316
14	C	6.147	6.9546	4.8344
15	C	6.3922	8.2425	4.3349
16	H	6.6328	9.0421	5.0271
17	C	6.2925	8.5115	2.9787
18	H	6.4493	9.5118	2.5959
19	C	6.1614	6.737	6.2904
20	H	6.4433	7.6149	6.8895
21	C	5.9136	5.5616	8.2905
22	H	6.2146	6.5205	8.7365
23	H	6.6879	4.8303	8.5534
24	C	4.6064	5.0449	8.9047
25	H	4.3546	4.1197	8.3851
26	H	4.8062	4.7743	9.9592
27	C	2.4665	11.2146	-1.1324
28	H	1.4049	10.9781	-1.0516
29	H	2.5973	11.7761	-2.0772
30	C	2.8042	12.1533	0.032
31	H	3.871	12.4203	0.0383
32	H	2.2444	13.0799	-0.1469
33	C	3.2112	11.606	2.2649
34	H	4.2399	11.9697	2.1265
35	C	2.9076	11.0885	3.6092
36	C	3.926	11.0461	4.5734
37	H	4.9053	11.4436	4.3279
38	C	3.7062	10.4735	5.8172
39	H	4.4997	10.4135	6.5512
40	C	2.4538	9.9306	6.1316
41	C	1.4195	10.0263	5.1887
42	H	0.4427	9.622	5.4316
43	C	1.6404	10.5944	3.9436
44	H	0.8511	10.6407	3.2042
45	C	2.2035	9.2251	7.3992
46	H	1.1705	8.8758	7.5414
47	C	2.7113	8.2912	9.4756
48	H	1.6482	8.0105	9.4601
49	H	2.8489	8.9744	10.3227
50	C	3.6061	7.0764	9.7504
51	H	4.6384	7.426	9.7104
52	H	3.4228	6.7423	10.7895
53	C	2.5546	8.9718	-2.0311
54	H	3.2538	8.1583	-2.2278

55	H	2.3013	9.4182	-3.0118
56	C	1.2841	8.3391	-1.4518
57	H	0.5313	9.1013	-1.2037
58	H	0.8573	7.7176	-2.249
59	C	0.9043	7.6473	0.7447
60	H	0.148	8.4418	0.8229
61	C	1.0896	6.8389	1.9611
62	C	2.0276	5.8006	2.0229
63	H	2.6006	5.5634	1.1358
64	C	2.2349	5.117	3.211
65	H	2.9806	4.3308	3.2575
66	C	1.5189	5.4515	4.3701
67	C	0.5454	6.4563	4.2955
68	H	-0.017	6.7054	5.1863
69	C	0.335	7.137	3.1058
70	H	-0.4043	7.9299	3.0616
71	C	1.8446	4.7706	5.6342
72	H	2.6109	3.9858	5.5563
73	C	1.7356	4.3173	7.9205
74	H	2.4994	3.5658	7.6738
75	H	0.8627	3.7732	8.3016
76	C	2.2019	5.2364	9.0565
77	H	1.4109	5.9698	9.2177
78	H	2.2707	4.6323	9.9814
79	N	3.2199	9.9542	-1.1651
80	N	3.4583	5.9572	8.8095
81	N	5.8367	8.9176	0.1318
82	N	5.8547	5.6331	6.8395
83	N	2.3705	11.6196	1.3128
84	N	3.1015	9.0043	8.2702
85	N	1.5692	7.4691	-0.323
86	N	1.3107	5.0648	6.7488

- Lowest energy conformation for **$BiPh_3T_2$**.

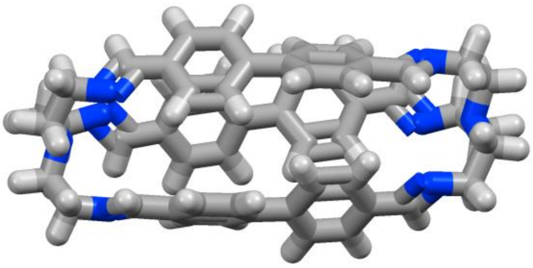

Cartesian coordinates (116 atoms), E (298.15 K) = -2527.95297 Hartree

1	C	4.8585	12.1083	-5.4195
2	H	5.0725	13.012	-4.8482
3	H	4.9385	12.3808	-6.4903
4	C	5.9748	11.106	-5.1039
5	H	5.8518	10.1721	-5.6715
6	H	6.907	11.5677	-5.4527

7	C	6.118	9.6497	-3.2896
8	H	6.0132	8.8196	-4.0042
9	C	6.2565	9.2412	-1.8831
10	C	6.2641	7.8784	-1.5619
11	H	6.2023	7.1422	-2.3571
12	C	6.344	7.4566	-0.24
13	H	6.3587	6.3957	-0.0197
14	C	6.4166	8.3855	0.8085
15	C	6.4368	9.7524	0.4764
16	H	6.4696	10.4925	1.2668
17	C	6.3572	10.1734	-0.8413
18	H	6.3448	11.2287	-1.0825
19	C	6.4302	7.9446	2.2238
20	C	5.6652	6.8381	2.6355
21	H	5.0535	6.3137	1.9117
22	C	5.6339	6.4379	3.961
23	H	5.0216	5.6002	4.2691
24	C	6.3653	7.1334	4.9336
25	C	7.134	8.2325	4.5312
26	H	7.7134	8.7784	5.2691
27	C	7.1703	8.6297	3.1988
28	H	7.7901	9.4711	2.9113
29	C	6.3334	6.7459	6.3533
30	H	6.8512	7.4357	7.037
31	C	5.8278	5.4186	8.2056
32	H	6.2688	6.2555	8.7664
33	H	6.4954	4.559	8.3472
34	C	4.4708	5.0011	8.7827
35	H	4.1354	4.1372	8.2081
36	H	4.6349	4.6509	9.8209
37	C	2.5637	12.8009	-5.1213
38	H	1.5481	12.4092	-5.0611
39	H	2.639	13.3492	-6.0808
40	C	2.717	13.8173	-3.9837
41	H	3.7149	14.2794	-3.9856
42	H	1.997	14.6187	-4.1919
43	C	3.2144	13.4152	-1.7405
44	H	4.1597	13.9564	-1.8957
45	C	3.0073	12.9056	-0.3758
46	C	3.9605	13.1762	0.6133
47	H	4.8249	13.7855	0.3691
48	C	3.8176	12.6707	1.9003
49	H	4.5644	12.9042	2.6502
50	C	2.7147	11.8743	2.2416
51	C	1.7491	11.6269	1.2493
52	H	0.9002	10.9935	1.4765
53	C	1.8908	12.1308	-0.0331

54	H	1.1569	11.9077	-0.7968
55	C	2.5861	11.2767	3.5918
56	C	3.7187	10.7982	4.2755
57	H	4.6922	10.8615	3.8046
58	C	3.6067	10.1958	5.5178
59	H	4.4815	9.8081	6.0237
60	C	2.3534	10.0449	6.1268
61	C	1.2224	10.5265	5.4565
62	H	0.2447	10.4265	5.9174
63	C	1.3358	11.1354	4.2118
64	H	0.4471	11.5169	3.7227
65	C	2.1996	9.3862	7.4332
66	H	1.1596	9.2278	7.7565
67	C	2.8836	8.3977	9.4328
68	H	1.8062	8.2199	9.563
69	H	3.1858	9.0974	10.2224
70	C	3.6994	7.12	9.6596
71	H	4.7501	7.3933	9.5588
72	H	3.5514	6.8011	10.7101
73	C	3.0584	10.6036	-6.0007
74	H	3.8625	9.8721	-6.0836
75	H	2.882	11.0014	-7.0194
76	C	1.8017	9.8385	-5.5707
77	H	0.928	10.5025	-5.4991
78	H	1.5899	9.1253	-6.3773
79	C	1.1357	9.1936	-3.4308
80	H	0.2727	9.8671	-3.5425
81	C	1.1915	8.4643	-2.1542
82	C	2.2513	7.6053	-1.8331
83	H	3.0532	7.4768	-2.5487
84	C	2.2888	6.9586	-0.6084
85	H	3.1386	6.3311	-0.3684
86	C	1.2732	7.1442	0.3469
87	C	0.2035	7.9876	0.0116
88	H	-0.6098	8.129	0.7139
89	C	0.165	8.6359	-1.2175
90	H	-0.6714	9.2857	-1.4547
91	C	1.3571	6.5007	1.6799
92	C	1.8871	5.2114	1.8375
93	H	2.1931	4.6461	0.9649
94	C	2.0117	4.6409	3.0997
95	H	2.4277	3.6431	3.1983
96	C	1.6044	5.335	4.2455
97	C	1.0594	6.6173	4.0918
98	H	0.7617	7.1649	4.9766
99	C	0.9401	7.1871	2.835
100	H	0.5622	8.1984	2.7474

101	C	1.7734	4.7187	5.5716
102	H	2.3133	3.7597	5.5762
103	C	1.5584	4.5194	7.8878
104	H	2.1949	3.6342	7.7437
105	H	0.5862	4.1545	8.2428
106	C	2.1013	5.4336	8.9919
107	H	1.384	6.2459	9.1136
108	H	2.1011	4.8617	9.9408
109	N	3.4972	11.6682	-5.0907
110	N	3.4187	6.0214	8.7295
111	N	6.1104	10.8592	-3.6782
112	N	5.7621	5.6947	6.7802
113	N	2.3836	13.2512	-2.6875
114	N	3.1798	9.0208	8.1537
115	N	2.0037	9.0745	-4.3509
116	N	1.3354	5.2372	6.6451

- Lowest energy conformation for **$(pPh)_2(BiPh)T_2$**.

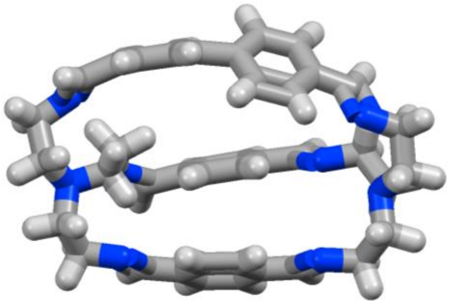

Cartesian coordinates (96 atoms), E (298.15 K) = -2065.839945 Hartree

1	N	-0.6391	5.624	0.848
2	N	-0.5469	4.1048	-1.8766
3	C	-0.2098	4.2308	0.5978
4	C	0.4148	3.9867	-0.7889
5	C	-3.1295	5.1821	0.4885
6	C	-2.0069	5.7197	1.3854
7	C	0.264	6.3699	1.732
8	C	1.6663	6.7363	1.1963
9	N	2.4872	5.5606	0.9722
10	C	3.1875	5.0942	1.9246
11	C	4.0783	-0.507	1.512
12	C	-3.8103	1.6317	-0.4798
13	C	-3.4889	0.829	0.6304
14	C	-3.4613	-0.5498	0.5191
15	C	-3.7557	-1.1719	-0.7068
16	C	-4.1208	-0.3759	-1.7978
17	C	-4.149	1.0095	-1.686
18	C	-3.6792	-2.6299	-0.8886
19	C	0.1726	-2.5501	-2.9535
20	C	2.8632	-4.5008	0.5617
21	N	1.902	-5.088	1.1483

22	C	1.4258	-6.3472	0.6047
23	C	-0.1003	-6.4706	0.6643
24	C	-2.3124	-5.7322	0.3074
25	C	-3.4089	-4.8645	-0.3136
26	N	-3.3435	-3.4491	0.0233
27	C	0.3621	-4.7507	-2.1795
28	C	-0.7243	-5.6574	-1.5697
29	N	0.1197	-3.3368	-1.9575
30	N	-0.9157	-5.5328	-0.122
31	C	3.1962	-1.2062	2.3569
32	C	2.8452	-2.5196	2.0946
33	C	3.3737	-3.1867	0.9808
34	C	4.3264	-2.5286	0.1938
35	C	4.6736	-1.2078	0.4535
36	C	3.7793	3.7476	1.8184
37	C	-0.4713	1.691	-2.6524
38	N	-3.2936	3.7338	0.5843
39	C	-3.7033	3.1003	-0.4383
40	C	-0.3592	0.8997	-1.4956
41	C	-0.1417	-0.4672	-1.5851
42	C	-0.0172	-1.092	-2.8346
43	C	-0.1055	-0.3059	-3.9903
44	C	-0.3478	1.0599	-3.899
45	C	3.9857	2.9856	2.9746
46	C	4.1806	1.6096	2.8897
47	C	4.1972	0.9642	1.645
48	C	4.1408	1.7611	0.4869
49	C	3.9253	3.1255	0.5705
50	C	-0.846	3.135	-2.6452
51	H	0.5274	3.9192	1.3462
52	H	-1.0664	3.5683	0.7199
53	H	0.934	3.0217	-0.7669
54	H	1.1718	4.7557	-0.9518
55	H	-4.0586	5.6387	0.8523
56	H	-2.9729	5.5122	-0.5464
57	H	-2.0981	5.2247	2.3696
58	H	-2.2095	6.7822	1.5457
59	H	-0.2231	7.3208	1.9659
60	H	0.4069	5.8409	2.6939
61	H	1.5594	7.2777	0.2526
62	H	2.1135	7.4167	1.9376
63	H	3.2753	5.6003	2.8986
64	H	-3.238	1.3138	1.5657
65	H	-3.1923	-1.1706	1.3649
66	H	-4.3608	-0.8457	-2.7459
67	H	-4.4065	1.6165	-2.5476
68	H	-3.9425	-2.9785	-1.8992

69	H	0.3619	-2.9192	-3.9746
70	H	3.3395	-4.9235	-0.3365
71	H	1.8053	-7.1525	1.2471
72	H	1.8165	-6.5462	-0.4037
73	H	-0.3843	-6.3385	1.7113
74	H	-0.3489	-7.5162	0.3928
75	H	-2.3384	-5.5644	1.3873
76	H	-2.6243	-6.7831	0.1432
77	H	-4.3434	-5.2496	0.1154
78	H	-3.4878	-5.0191	-1.3995
79	H	0.4357	-4.9942	-3.2529
80	H	1.3306	-4.9838	-1.7323
81	H	-0.4903	-6.6986	-1.857
82	H	-1.6596	-5.4098	-2.072
83	H	2.72	-0.6841	3.1783
84	H	2.1108	-3.0287	2.7059
85	H	4.7661	-3.0418	-0.6555
86	H	5.3868	-0.7064	-0.1904
87	H	-3.9425	3.6139	-1.3803
88	H	-0.4946	1.3452	-0.5195
89	H	-0.086	-1.0766	-0.6921
90	H	-0.0095	-0.7724	-4.9657
91	H	-0.4521	1.648	-4.805
92	H	3.9159	3.4575	3.9494
93	H	4.2748	1.0279	3.7994
94	H	4.1676	1.2879	-0.4875
95	H	3.787	3.7142	-0.3273
96	H	-1.5136	3.3904	-3.4758

- Lowest energy conformation for **(pPh)(BiPh)$_2$T$_2$**.

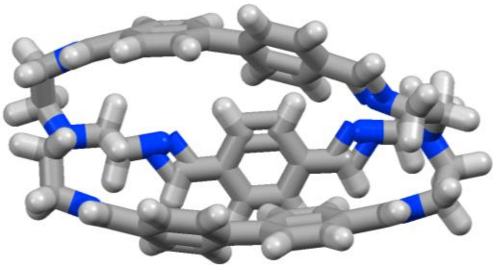

Cartesian coordinates (106 atoms), E (298.15 K) = -2296.902849 Hartree

1	N	0.1438	6.3903	1.8387
2	N	-2.8831	6.0572	1.2968
3	C	-0.9653	7.1109	2.4634
4	C	-2.266	7.3224	1.6387
5	C	0.6039	4.2597	3.0828
6	C	0.0029	4.9257	1.8322
7	C	0.6375	6.9898	0.6034
8	C	2.1633	6.8947	0.3764
9	N	2.613	5.5244	0.205
10	C	2.8088	5.0885	-0.9737

11	C	3.2412	-0.5958	-1.7629
12	C	1.0916	0.5461	3.0335
13	C	-3.485	0.2818	-1.5261
14	C	-0.1513	-0.0282	2.7108
15	C	-0.283	-1.3996	2.5752
16	C	0.8225	-2.2483	2.7627
17	C	2.0544	-1.679	3.106
18	C	2.1889	-0.2991	3.2337
19	C	-2.7578	-0.5597	-0.6637
20	C	-2.4644	-1.8644	-1.0131
21	C	-2.8999	-2.388	-2.2386
22	C	-3.6623	-1.5731	-3.0837
23	C	-3.9465	-0.254	-2.7362
24	C	0.7218	-3.7133	2.6281
25	C	-2.4932	-3.7453	-2.6458
26	C	2.5064	-4.8197	-2.0821
27	N	2.3719	-5.563	-1.0616
28	C	1.9045	-6.9266	-1.2324
29	C	0.8736	-7.2793	-0.1493
30	C	-1.0331	-6.4527	1.1337
31	C	-0.3171	-5.7792	2.3108
32	N	-0.3359	-4.3224	2.2735
33	C	-1.2417	-5.7244	-2.4072
34	C	-1.2348	-6.7612	-1.2729
35	N	-1.6877	-4.4292	-1.9421
36	N	-0.3301	-6.448	-0.156
37	C	3.4168	-1.2611	-2.986
38	C	3.207	-2.6336	-3.089
39	C	2.8384	-3.3885	-1.9689
40	C	2.6978	-2.7335	-0.7385
41	C	2.8894	-1.3677	-0.6403
42	C	3.1189	3.6717	-1.2315
43	C	-3.4373	4.4107	-0.3621
44	N	0.3112	2.8341	3.0438
45	C	1.2714	2.0078	3.1347
46	C	-3.739	3.4093	0.5736
47	C	-3.8404	2.0868	0.1793
48	C	-3.6375	1.7101	-1.1615
49	C	-3.4416	2.7252	-2.1082
50	C	-3.3429	4.0559	-1.7133
51	C	2.8484	3.1074	-2.4834
52	C	2.9346	1.732	-2.6772
53	C	3.3198	0.879	-1.6324
54	C	3.6901	1.469	-0.4089
55	C	3.5795	2.8332	-0.2065
56	C	-3.0479	5.7619	0.0706
57	H	-1.2308	6.583	3.3832

58	H	-0.612	8.1052	2.7604
59	H	-2.0635	7.94	0.7495
60	H	-2.9514	7.8848	2.2844
61	H	1.6806	4.4728	3.1427
62	H	0.1236	4.678	3.9755
63	H	0.519	4.5267	0.9602
64	H	-1.0449	4.6225	1.7613
65	H	0.4036	8.0582	0.6342
66	H	0.1394	6.5839	-0.2928
67	H	2.6691	7.3192	1.2482
68	H	2.4001	7.5154	-0.5029
69	H	2.6902	5.7311	-1.86
70	H	-0.9987	0.6291	2.5605
71	H	-1.2383	-1.8425	2.3218
72	H	2.9146	-2.3211	3.2645
73	H	3.1538	0.1271	3.4888
74	H	-2.3679	-0.1595	0.264
75	H	-1.8623	-2.4924	-0.3689
76	H	-4.0192	-1.9662	-4.0306
77	H	-4.5289	0.3631	-3.4111
78	H	1.6412	-4.2632	2.8806
79	H	-2.8958	-4.1103	-3.6041
80	H	2.3361	-5.1933	-3.1036
81	H	1.5083	-7.1111	-2.2426
82	H	2.7544	-7.6066	-1.0924
83	H	1.3746	-7.1513	0.8108
84	H	0.6261	-8.354	-0.244
85	H	-1.9796	-5.9279	0.9952
86	H	-1.2779	-7.485	1.4513
87	H	0.7009	-6.1707	2.4466
88	H	-0.8838	-6.0713	3.2046
89	H	-0.2195	-5.6027	-2.7795
90	H	-1.8546	-6.1084	-3.2402
91	H	-2.2585	-6.8535	-0.8975
92	H	-0.983	-7.7388	-1.7103
93	H	3.7126	-0.7032	-3.8669
94	H	3.3234	-3.1215	-4.0516
95	H	2.3793	-3.3103	0.1198
96	H	2.7015	-0.8821	0.3085
97	H	2.3093	2.3436	3.2801
98	H	-3.8414	3.6825	1.6163
99	H	-4.0458	1.3248	0.9219
100	H	-3.2845	2.4652	-3.1486
101	H	-3.1232	4.8187	-2.4535
102	H	2.5064	3.7413	-3.2952
103	H	2.6391	1.3143	-3.6323
104	H	4.0483	0.8435	0.3995

105	H	3.824	3.2692	0.754
106	H	-2.8034	6.4722	-0.7338
